# Unraveling candidate genes underlying biomass digestibility in elephant grass (*Cenchrus purpureus*)

**DOI:** 10.1186/s12870-019-2180-5

**Published:** 2019-12-10

**Authors:** João Romero do Amaral Santos de Carvalho Rocha, Tiago de Souza Marçal, Felipe Vicentino Salvador, Adriel Carlos da Silva, Pedro Crescencio Souza Carneiro, Marcos Deon Vilela de Resende, Jailton da Costa Carneiro, Ana Luisa Sousa Azevedo, Jorge Fernando Pereira, Juarez Campolina Machado

**Affiliations:** 1 Universidade Federal de Viçosa, Viçosa, MG 36570-900 Brazil; 2 Embrapa Florestas, Colombo, PR 83411-000 Brazil; 3 Embrapa Gado de Leite, Juiz de Fora, MG 36038-330 Brazil

**Keywords:** Gene annotation, Napier grass, *Pennisetum purpureum*, SSR marker, Trait-marker association

## Abstract

**Background:**

Elephant grass [*Cenchrus purpureus* (Schumach.) Morrone] is used for bioenergy and animal feed. In order to identify candidate genes that could be exploited for marker-assisted selection in elephant grass, this study aimed to investigate changes in predictive accuracy using genomic relationship information and simple sequence repeats for eight traits (height, green biomass, dry biomass, acid and neutral detergent fiber, lignin content, biomass digestibility, and dry matter concentration) linked to bioenergetics and animal feeding.

**Results:**

We used single-step, genome-based best linear unbiased prediction and genome association methods to investigate changes in predictive accuracy and find candidate genes using genomic relationship information. Genetic variability (*p* < 0.05) was detected for most of the traits evaluated. In general, the overall means for the traits varied widely over the cuttings, which was corroborated by a significant genotype by cutting interaction. Knowing the genomic relationships increased the predictive accuracy of the biomass quality traits. We found that one marker (M28_161) was significantly associated with high values of biomass digestibility. The marker had moderate linkage disequilibrium with another marker (M35_202) that, in general, was detected in genotypes with low values of biomass digestibility. In silico analysis revealed that both markers have orthologous regions in other C4 grasses such as *Setaria viridis*, *Panicum hallii*, and *Panicum virgatum*, and these regions are located close to candidate genes involved in the biosynthesis of cell wall molecules (xyloglucan and lignin), which support their association with biomass digestibility.

**Conclusions:**

The markers and candidate genes identified here are useful for breeding programs aimed at changing biomass digestibility in elephant grass. These markers can be used in marker-assisted selection to grow elephant grass cultivars for different uses, e.g., bioenergy production, bio-based products, co-products, bioactive compounds, and animal feed.

## Background

Elephant grass [*Pennisetum purpureum* Schumach. syn. *Cenchrus purpureus* (Schumach.) Morrone] is a perennial tropical grass with high photosynthetic efficiency (C4 photosynthetic pathway) that is naturally found in several African countries [1]. It has a range of adaptation to different levels of altitude, precipitation, and soils, and has important agronomic traits [2], particularly its high biomass production. Depending on the environment and cultivar characteristics, green biomass can reach 300 Mg ha^− 1^ year^− 1^ [3]; consequently, elephant grass is used for multiple purposes, including the production of bio-based compounds [4] and molecules with pharmaceutical and industrial applications [5–7]. It has been targeted by bioenergy programs, because its annual dry matter production is greater than that of sugarcane or eucalyptus, which are the most-used biomass energy sources in Brazil [2, 8]. However, the most common use for elephant grass in Brazil is in animal feed, particularly for dairy cattle.

Elephant grass is a tetraploid species (2n = 4x = 28) with two genomes (A′A′BB) [9]. Its genome is homologous to that of pearl millet [*Pennisetum glaucum* (L.) R. Br. syn. *Cenchrus americanus* (L.) Morrone], and cytogenetic studies have been conducted on these species in order to identify hybrids [9, 10]. Elephant grass has not been significantly studied, so its genomic information is little-known compared to that of more economically important crops. An RNAseq study [11], two studies on genome sequencing [12, 13], and one transcriptomic and metabolomic study [7] have been published for elephant grass. However, much is still needed to be elucidated for the next-generation breeding of elephant grass [14]. For example, few trait-marker association studies have been conducted because of the low availability of molecular markers, but simple sequence repeat (SSR) markers have been transferred from pearl millet to elephant grass [15] and used in genetic diversity studies to increase gene bank diversity [16]. Although SSR markers appear in the genome at a lower frequency than single nucleotide polymorphisms (SNPs), they can be used in genomic association studies. For example, SSR markers have been used to identify loci associated with yield components and fiber quality in cotton [17], resistance to *Sclerotinia sclerotiorum* in *Brassica napus* [18], and quantitative traits contributing to yield in sugarcane [19]. In general, many SSR markers are used in trait-marker association studies, but few may provide satisfactory results in species with limited sequence information, such as creeping bentgrass (*Agrostis stolonifera* L.) [20].

Genomic association studies identify candidate genes and markers linked to important traits. When considering multipurpose traits (i.e., traits with different purposes, such as animal feeding and bioenergy), the development of selection procedures based on molecular markers can radically streamline and accelerate elephant grass improvement [21]. Appropriate targets for breeding elephantgrass for forage and bioenergy use are agronomic traits (height, green biomass, and dry biomass) and quality traits (acid and neutral detergent fiber, lignin content, biomass digestibility, and dry matter concentration). Because elephant grass is vegetatively propagated and crosses are not widely used, the use of germplasm collections for association mapping is based on historical and naturally occurring recombination events.

In this context, the goals of this study were to (*i*) investigate changes in predictive accuracy using genomic relationship information in statistical models, (*ii*) investigate significant associations between SSR markers and eight traits, evaluated in different cuttings, in a germplasm collection of elephant grass genotypes, and (*iii*) identify candidate genes linked to these traits with their respective gene annotations. By achieving these goals, we aimed to increase the speed and accuracy of breeding elephant grass for different purposes.

## Results

### Genetic variation, genotype by cutting interaction, and predictive accuracy using a single-step, genome-based best linear unbiased prediction (ssGBLUP) model

**Fig. 1:**
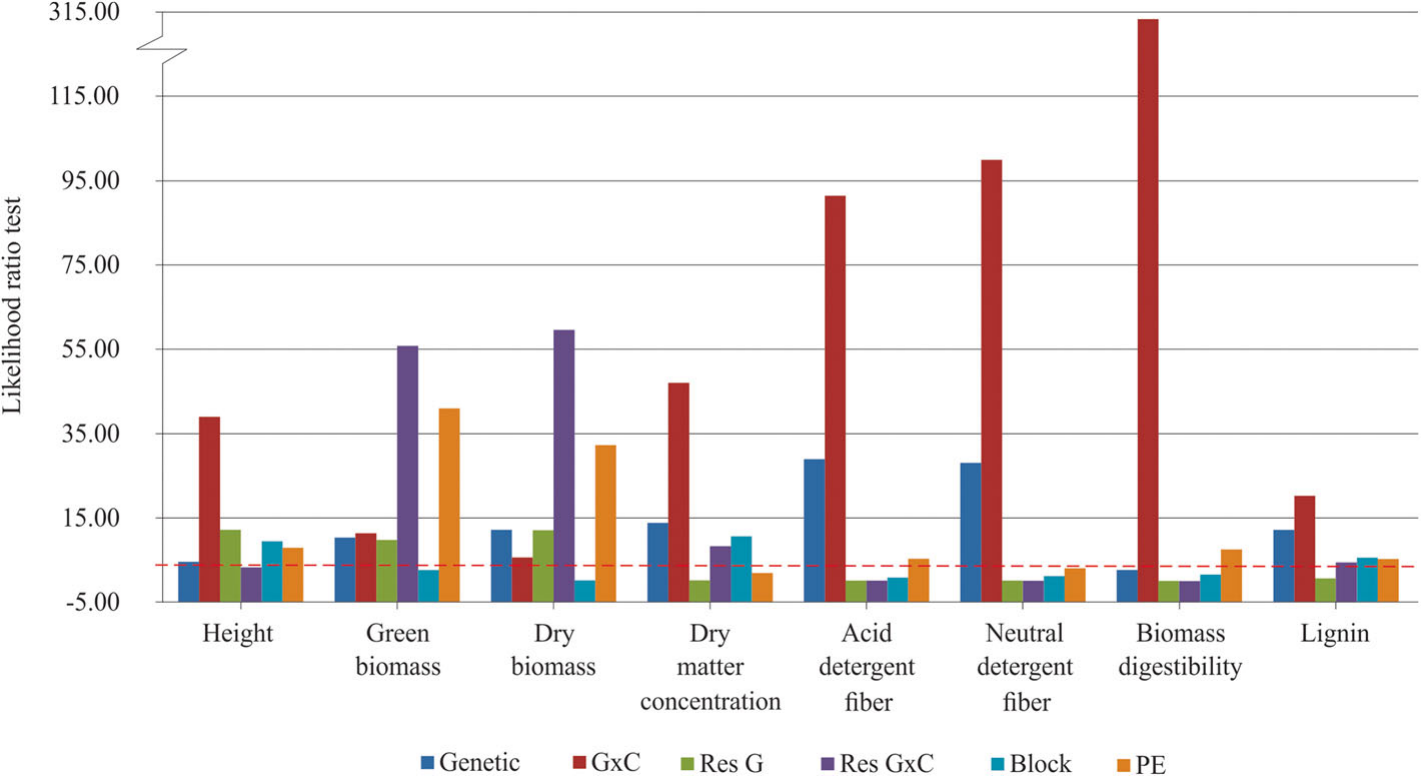
Likelihood ratio tests for genetic effects (Genetic), genotype by cutting effects (G × C), residual genetic effects (Res G), residual genotype by cutting interaction effects (Res G × C), block effects (Block) and permanent environment effects (PE) considering ssGBLUP model. All bars above the dashed red line are significant by chi-square test at 5% probability $\left({X}_{5\%}^2=3.84\right)$

Initially, a ssGBLUP model was used to fit the full dataset in order to investigate genetic variability and the genotype by cutting interaction and residual genetic variability and the genotype by cutting interaction. Genetic variability (*p* < 0.05) was detected for seven traits (height, green biomass, dry biomass, dry matter concentration, acid detergent fiber, neutral detergent fiber, and lignin content) but not biomass digestibility (Fig. 1). Regarding the genotype by cutting interaction, a significant effect (*p* < 0.05) for all traits was observed (Fig. 1). It is noteworthy that, in the individual analysis of each cutting, significant genetic variability was detected for all traits.

Log-likelihood ratio tests revealed that residual genetic effects were significant (*p* < 0.05) for height, green biomass, and dry biomass but not for biomass quality traits (i.e., dry matter concentration, acid detergent fiber, neutral detergent fiber, biomass digestibility, and lignin content) (*p* > 0.05) (Fig. 1). There was a significant residual genotype by cutting interaction (*p* < 0.05) for green biomass, dry biomass, dry matter concentration, and lignin (Fig. 1).

**Fig. 2:**
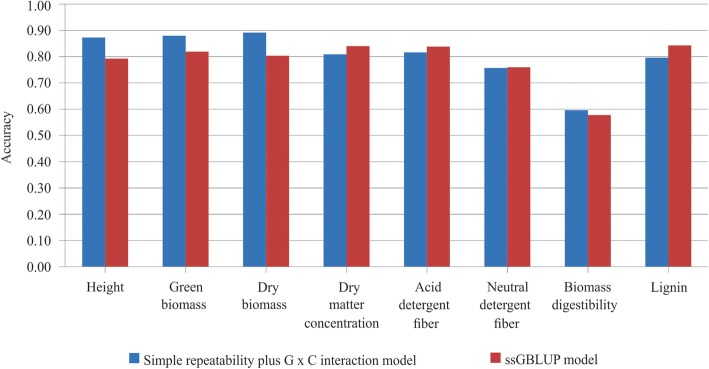
Accuracy of breeding value for the simple repeatability plus genotype by cutting interaction model and ssGBLUP model

The predictive accuracy of the ssGBLUP model (considering the *H*^*− 1*^ matrix) ranged from 0.58 (biomass digestibility) to 0.84 (acid detergent fiber, dry matter concentration, and lignin). When the model did not include the relationship between the genotypes (simple repeatability plus the genotype by cutting interaction model), the accuracy ranged from 0.59 (biomass digestibility) to 0.89 (dry biomass). Inclusion of the relationship matrix in the ssGBLUP model increased the accuracy of the biomass quality traits (Fig. 2), except for biomass digestibility.

### Overall means and accuracies for each cutting

**Fig. 3:**
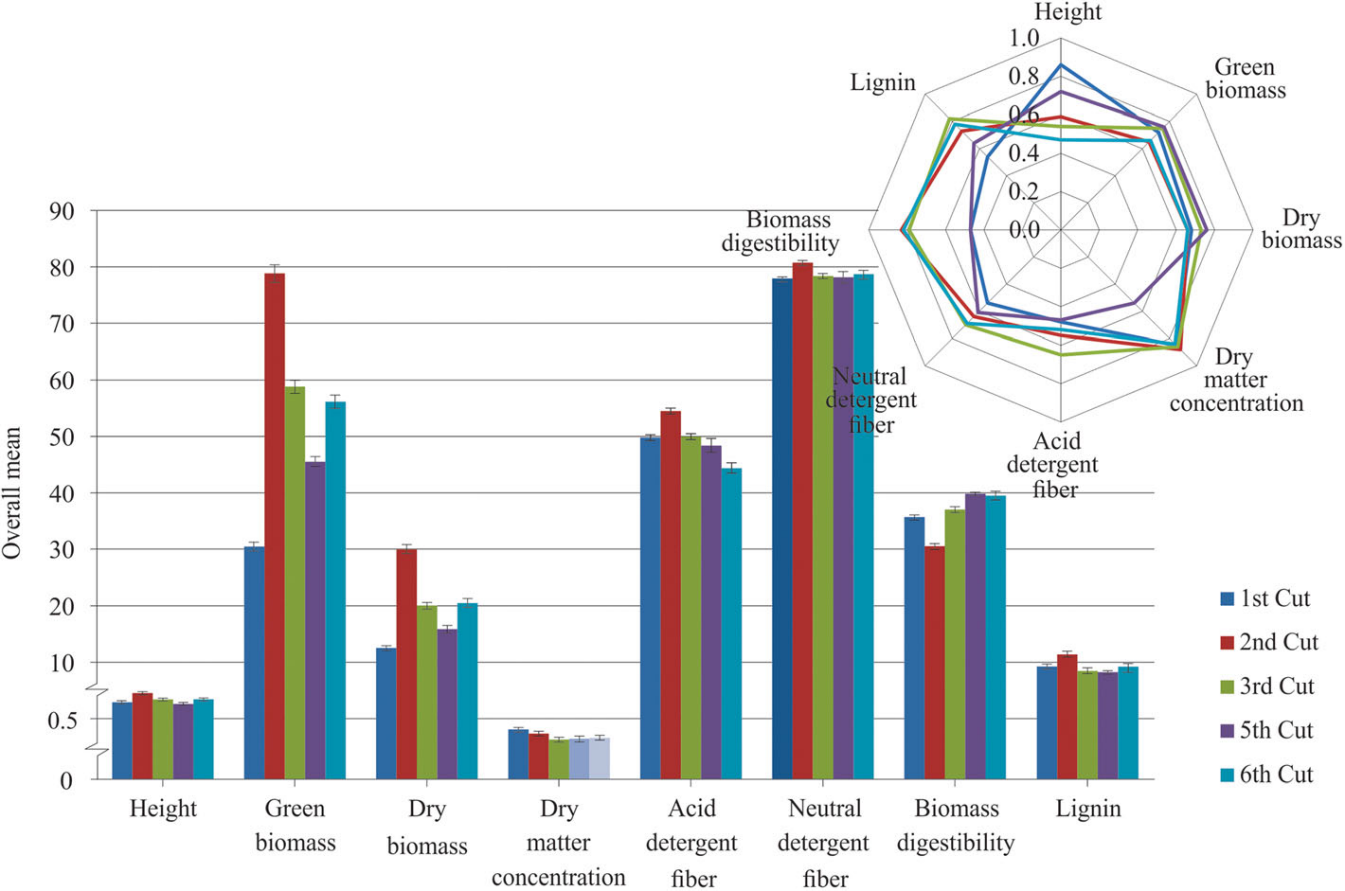
Overall means (bars plot) with the standard errors and accuracy of breeding values (radar plot) for five cuttings recorded in 90 genotypes of elephant grass

Because a significant effect of the genotype by cutting interaction was observed for all traits (ssGBLUP model, Fig. 1), trait-marker associations were investigated for each cutting. The overall means and accuracies of each cutting are presented in Fig. 3. The accuracy values ranged from 0.47 (acid detergent fiber and biomass digestibility for the fifth cutting and biomass digestibility for the first cutting) to 0.88 (dry matter concentration for the second cutting). In general, the overall means for all traits varied widely among the cuttings, corroborating the significant genotype by cutting interaction. For example, the second cutting had the highest values for height, green biomass, dry biomass, acid detergent fiber, neutral detergent fiber, and lignin, but had the lowest values for biomass digestibility.

### Genome association study

**Fig. 4:**
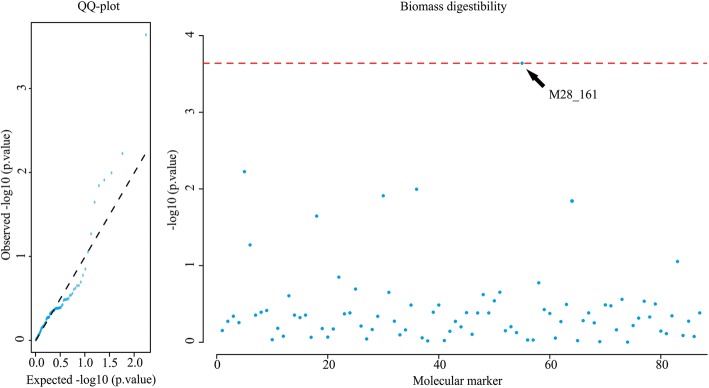
QQ-plot (on the left) and Manhattan plot (on the right) for genome association of biomass digestibility phenotype with SSR markers on the first cut of elephant grass. Dashed red lines on Manhattan plot (false discovery rate) indicate the minimum threshold to select significant markers. The arrow highlights a major trait-marker association

We analyzed 90 elephant grass genotypes that had phenotypic data and SSR alleles available, and identified one allele of the M28 marker (M28_161) that was significantly associated with biomass digestibility. This association was detected only for the first cutting. A Manhattan plot (Fig. 4, right) shows the − log(*P* − *value*) for all SSRs that affected biomass digestibility, while the quantile-quantile (QQ) plot (Fig. 4, left) displays significant deviations of the observed − log(*P* − *value*) from those expected.

**Fig. 5:**
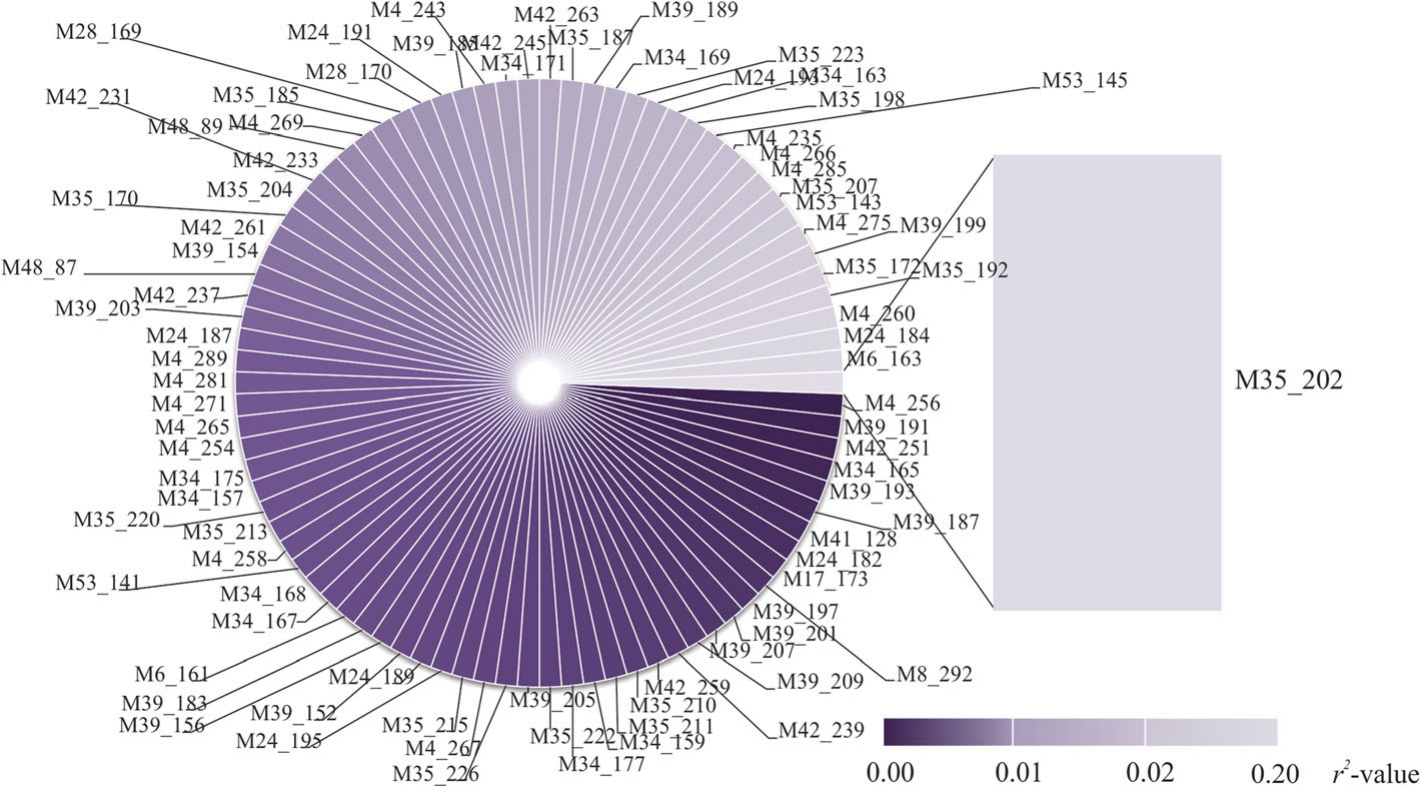
Linkage disequilibrium (*r*^2^-values) between all markers used in this study with the marker M28_161, which was found as significantly associated with biomass digestibility in elephant grass

To identify the linkage of all markers, we performed linkage disequilibrium analysis (Fig. 5). In this analysis, we were interested in identifying markers that may have been linked to M28_161, which was significantly linked to high values of biomass digestibility. One allele of the SSR marker M35 (M35_202) showed moderate linkage disequilibrium (*r*^2^ = 0.20) to M28_161, and, in general, its presence was detected in elephant grass genotypes with low values of biomass digestibility. All other *r*^2^-values were lower than 0.11, and varied from 0.00 to 0.11.

### Annotation of M28_161 and M35_202 markers, in silico pathway analysis, and allelic contribution

A BLAST search revealed that the two SSR markers (M28_161 and M35_202) are close to candidate genes that have annotated functions in other grasses (Table 1). In some cases (i.e., *Setaria viridis* chromosome 3 and *Panicum halli* chromosomes 5 and 7), M28_161 is close to candidate genes linked to pathways influenced by plant hormones (salicylic acid or abscisic acid). However, in most cases, M28_161 and M35_202 are associated with candidate genes involved in the synthesis of cell wall components. In *P. halli*, M28_161 is close to candidate genes involved in lignin biosynthetic processes and cell wall organization (chromosome 7), and in *Panicum virgatum*, it is close to a candidate gene involved in lignin catabolic processes and oxidation-reduction processes (located on chromosome 8). In *S. viridis*, both M28_161 and M35_202 were found to have orthologous regions. M28_161 is close to a candidate gene on chromosome 3 that plays a role in xyloglucan biosynthesis, while M35_202 is close to a candidate gene on chromosome 7 that functions in lignin biosynthesis. For *Setaria italica*, a successful BLAST search was conducted for the M28_161 sequence, but no candidate genes related to digestibility were found. However, for *C. americanus*, no BLAST results were obtained when using the M28 and M35 sequences.

**Table 1:** Candidate genes, in the genome of other C4 species, that are near to homologous sequences to the SSR markers M28_161 and M35_202

Marker	Candidate gene	Reference genome	Chr^a^	Biological pathway	Ortholog locus on *A. thaliana*	Locus position^b^	Marker position
M28_161	Sevir. 3G340800	*Setaria viridis*	3	Xyloglucan biosynthetic process; salicylic acid mediated	AT2G20370	40,675,315	40,676,270
M28_161	Pahal. G00901	*Panicum halli*	7	Lignin biosynthetic process; cell wall organization	AT5G48930	29,711,375	29,776,838
M28_161	Pahal. G00889	*Panicum halli*	7	Salicylic acid mediated	AT5G05190	29,776,838	29,776,838
M28_161	Pahal. E03247	*Panicum halli*	5	Response to abscisic acid	AT3G05880	49,635,160	49,627,286
M28_161	Pavir. 8KG357500	*Panicum virgatum*	8	Lignin catabolic process, oxidation-reduction process	AT3G09220	72,822,520	72,841,944
M35_202	Sevir. 7G164200	*Setaria viridis*	7	Lignin biosynthetic process; cell wall organization	AT5G48930	23,027,630	23,030,255

^a^Chromosome; ^b^Locus that is closest to the SSR marker

## Discussion

### Genetic variation and genotype by cutting interaction

We observed a significant effect of the genotype by cutting interaction for all eight traits analyzed, so the genomic association study was conducted by considering a single cutting at a time. The lack of genetic variability (i.e., for biomass digestibility) revealed by the ssGBLUP analysis does not mean that there was no genetic variability, because the interaction effect may have reduced it. Residual terms were added to the ssGBLUP model to capture nonadditive effects. For crops that exhibit clonal propagation and are not lines (such as elephant grass), nonadditive effects represent epistatic and dominance effects [22], and additive effects were not explained by the genomic relationship matrix, so these effects would not have inflated the residual estimates or overlaid the other effects. The nonsignificant residual genetic effect for the biomass quality traits indicates that part of the additive fraction that plays a role in the genetic architecture of these traits could be explained by only a few markers (87 alleles). For the morpho-agronomic traits the residual genetic effect was significant, so the genomic relationship matrix constructed from 87 markers did not explain the additive genetic fraction for height, green biomass, and dry biomass.

### Predictive accuracy

Cell component fractions that could affect forage digestibility include cellulose, lignin, hemicellulose, and cell wall proteins [23]. Therefore, biomass digestibility is dependent upon several components which makes it a complex trait that may be explained by the fact of the accuracy has been moderate magnitude considering the genomic information. Recent transcriptomic and genomic studies [7, 11–13] and the development of SNPs for elephant grass [12] have increased accuracy, and studies using molecular markers have been important for the development of SNP markers. New genotyping, sequencing, and bioinformatics tools have increased accuracy and decreased the price per sequenced base or molecular genotype [14], and selection time [24].

### Trait-marker association analysis

None of the agronomic traits (height, green biomass, and dry biomass) and four of the quality traits (acid and neutral detergent fiber, dry matter concentration, and lignin content) were associated with SSR markers. This was probably related to the low number of SSR markers used (18 SSR markers that originated in 87 alleles) when compared to the genome size of elephant grass, which has recently been estimated as 2.1 Gb [13]. However, one quality trait (biomass digestibility) was associated with the SSR marker M28_161 when the dataset from the first cutting was analyzed. This marker was in linkage disequilibrium with M35_202, so is associated with biomass digestibility. However, these markers are linked to different values of biomass digestibility, because M28_161 was significantly associated with high values of biomass digestibility while M35_202 was more frequently detected in genotypes with low values of biomass digestibility. This is an interesting result considering that digestibility is an important trait for many plant species, and understanding its impact on plant quality and the genomic regions associated with it has been the focus of many research groups [25–27].

The SSR markers used here were previously developed for pearl millet [28, 29], and were identified by our group to cross-amplify the elephant grass genome [15]. It is unknown to what extent these markers are distributed in the elephant grass genome, but our analysis shows that some markers have moderate linkage disequilibrium. The average similarity coefficient among 107 accessions of the Active Elephant Grass Germplasm Bank maintained by Embrapa Gado de Leite (where the genotypes were obtained) was 0.651, which indicates genetic variability [15]. In addition, Azevedo et al. [15] detected only one group of similarity for the genotypes used in the present study. In this context, neither the small number of markers used here nor the absence of population structure correction were obstacles to detect marker M28_161 as being significantly associated with biomass digestibility. Although trait-marker association analysis can be performed using non-specific markers (such as diversity arrays technology) or by SNP genotyping, SSR markers are important when genome sequencing and bioinformatics are not trivial tasks.

### Marker annotation and in silico pathway

We ran BLAST searches using sequences of the markers M28_161 and M35_202, and the genome of the related C4 grasses *S. italica*, *S. viridis*, *P. virgatum*, *P. halli*, and *C. americanus*. These species are closely related to elephant grass [30, 31]. No results were found for the *C. americanus* genome sequence, which was unexpected because the SSR markers used here were developed from pearl millet [15] and the A′A′ genome of elephant grass is homologous to the A genome of pearl millet [9, 10]. For the other species, six candidate genes that are associated with the biosynthesis of cell wall molecules were identified.

One candidate gene annotated on chromosome 3 of *S. viridis* (Sevir. 3G340800) is orthologous to locus AT2G20370 of *Arabidopsis thaliana*, and codes for a xyloglucan galactosyltransferase responsible for different functions, including the synthesis of cell wall materials. Xyloglucan, which is a component of the plant cell wall, is a type of hemicellulose that has the ability to bind to cellulose to form a cellulose-xyloglucan network linked through hydrogen bonds (see review by Pauly and Keegstra [23]). It is unclear whether xyloglucan decreases biomass digestibility, but it is associated with lignin, which is a cell wall component that commonly negatively affects digestibility. Xyloglucan binds to cellulose to form an aggregate that, in some types of cell, can be embedded in a matrix that contains lignin [23]. However, it is important to note that although xyloglucan is the most abundant hemicellulose in the primary cell walls of dicotyledons, in grasses such as elephant grass, its abundance is lower [23]. The effects of xyloglucan galactosyltransferase on biomass digestibility in elephant grass should be carefully evaluated.

No markers were associated with lignin content. Lignin is the second-most abundant biopolymer on Earth [32], and is a highly condensed phenylpropanoid matrix that is relatively difficult to digest by ruminal microorganisms and intestinal enzymes [33]. In many plant species, there is a negative correlation between lignin content and digestibility [34, 35] that can affect animal performance, because a small increase in dry matter digestibility (1%) can increase beef cattle daily weight gains by 3.2% [36]. In general, the quality of forage grasses decreases as they mature as a consequence of secondary cell wall deposition and the lignification of sclerenchyma cells. This does not mean that decreasing the lignin content is the only way to improve plant digestibility, but it is commonly accepted that lignin is a potential target for that purpose. In this context, the candidate genes Pahal. G00901, Pavir. 8KG357500, and Sevir. 7G164200, annotated in the *P. halli*, *P. virgatum*, and *S. viridis* genomes, respectively, are useful assets. These candidate genes are orthologous to the loci AT5G48930, AT3G09220, and AT5G48930 in *Arabidopsis*. AT5G48930 is a hydroxycinnamoyl-Coenzyme A shikimate/quinate hydroxycinnamoyltransferase that is involved in the phenylpropanoid pathway, and plays a role in the production of hydroxycinnamyl alcohols (or monolignols) that serve as the building blocks of lignin [37]. The lignin content can also affect bioenergy production [38], and can be decreased or increased depending on how the biomass is treated for energy generation. For example, for the conversion of lignocellulosic biomass to ethanol, polysaccharides from the cell wall need to be hydrolyzed to simple sugars and then fermented to ethanol [39]. In this case, reducing the lignin content can increase ethanol production through conventional biomass fermentation [38]. However, when considering biomass combustion, more lignin is needed for high energy conversion [40]. The molecular basis of lignin content in elephant grass is unknown, but the candidate genes identified here can be used as targets for gene manipulation. Either by transgenesis or gene editing, the manipulation of genes associated with lignin production can result in germplasm that is ideal for animal feeding or bioenergy [35, 38, 41, 42].

Some candidate genes are associated with plant hormones (salicylic acid or abscisic acid), the cross-talk of which plays an important role in the molecular responses of plants to stress [43], and two of them are involved in responses to biotic and abiotic stressors. The candidate gene Pahal. G00889 (annotated on chromosome 7 of *P. halli*) is orthologous to locus AT5G05190 in *Arabidopsis*, in which a hypothetical protein is involved in different functions, including responses to fungi [44]. The candidate gene Pahal. E03247 (also from *P. halli*) has an orthologous locus in *Arabidopsis* (AT3G05880) that codes for a small and highly hydrophobic protein that is involved in the hyperosmotic salinity response and response to cold. Validating these candidate genes in elephant grass would increase our knowledge of how this species responds to stressors such as spittlebug (Cercopidae) attack and drought or flooding, which are important factors for breeding programs in Brazil [14].

## Conclusions

This study showed that, even by using a few SSR markers, it is possible to identify candidate genes associated with biomass digestibility. It also showed that it was possible to increase predictive accuracy in elephant grass by incorporating genomic relationship information. Because there is little genomic information for elephant grass available, our findings may improve elephant grass breeding. For example, marker-assisted selection can be applied, and markers associated with biomass digestibility have the potential to drive elephant grass selection for different uses (e.g., bioenergy production and animal feed). Further validation of the candidate genes revealed here may lead to a better understanding of biomass digestibility variation and its genomic basis.

## Methods

### Plant materials and experimental information

One hundred elephant grass genotypes (Additional file 1: Table S1) from the Active Elephant Grass Germplasm Bank (Embrapa Gado de Leite, Brazil) were planted in 0.20-m-deep furrows with 80 kg ha^− 1^ P_2_O_5_ fertilizer applied at planting on December 23rd, 2011. The red-yellow latosol soil at the Embrapa Gado de Leite experimental station in Coronel Pacheco, MG, Brazil (latitude 21°33′18′′ S, longitude 43°15′51′′ W, 417 m.a.s.l.) had the following chemical properties: pH (5.4), H + Al (2.31 cmolc dm^− 3^), P (1.1 cmolc dm^− 3^), K (23 mg dm^− 3^), and exchangeable cations Al^3+^ (0.2 cmolc dm^− 3^), Ca^2+^ (1.4 cmolc dm^− 3^), and Mg^2+^ (0.7 cmolc dm^− 3^). The plots consisted of 4-m rows that were planted side by side, 1.5 m apart. Plots were allocated in a 10 × 10 simple lattice design, with two replications. At 30 days after planting, the plots were cut to 0.30-m stubble height (uniformly cut). The number of days to reach each of the six growing seasons (cuttings) started at this time. Maintenance fertilization was made with 300 kg ha^− 1^ of a N-P_2_O_5_-K_2_O formulation (20:05:20 blended granular fertilizer) after all cuttings. Fertilization was conducted according to the results of a soil analysis.

Six cuttings were conducted: the first cutting (September 28th, 2012) was performed at 250 regrowth days, the second (June 5th, 2013) at 250 regrowth days, the third (April 16th, 2014) at 315 regrowth days, the fourth (January 16th, 2015) at 275 regrowth days, the fifth (November 27th, 2015) at 315 regrowth days, and the sixth (June 24th, 2016) at 210 regrowth days. The fourth cutting collected propagation material, i.e., no phenotypic data were obtained at this cutting. We declare that all plant materials in this study represent voucher specimens that have been deposited in a publicly available herbarium (Active Elephant Grass Germplasm Bank) and that comply with institutional, national, and international guidelines for the collection and cultivation of any plant materials.

### Phenotypic traits

The plants were phenotyped at first, second, third, fifth, and sixth cuttings for each of the following traits: (*i*) height (m) was obtained from the arithmetic mean of the height of three randomly selected plants in each plot, measured from ground level to the curve of the last completely expanded leaf; (*ii*) green biomass (Mg ha^− 1^) was obtained from a cutting taken at 7.5 cm stubble height in a 3-m section in the middle of the rows using a gasoline-powered strimmer and that was collected by hand. The 3-m section was immediately weighed in the field to provide estimates of green biomass; (*iii*) dry biomass (Mg ha^− 1^) was quantified by multiplying the green biomass by the dry matter concentration (%); (*iv*) acid detergent fiber (g Kg^− 1^), (*v*) neutral detergent fiber (g Kg^− 1^), and (*vi*) lignin content (g Kg^− 1^) were determined following the methodology proposed by Goering [45]; (*vii*) biomass digestibility (g Kg^− 1^) was determined by the method described by Tilley and Terry [46]; and (*viii*) dry matter concentration (%) was obtained by sampling three complete plants from each plot, which were dried in a kiln after weighing (fresh weight) until weight stabilization. The samples were weighed (dry weight) again, and the dry matter concentration was determined by the ratio between dry weight and fresh weight. This trait was used as a common denominator for the estimation of biomass digestibility. For acid detergent fiber, neutral detergent fiber, lignin content, and biomass digestibility, random samples of three complete plants from each plot were collected before cutting the experimental plots. These samples were dried in a forced-air circulation oven at 56 °C for 72 h. After drying, the samples were ground to small particles (1 mm) in a Wiley type grinder and analyzed as described above. For the third, fifth, and sixth cuttings, acid and neutral detergent fiber, lignin content, biomass digestibility, and dry matter concentration were measured using near-infrared spectroscopy (NIRS). Data generated from the first and second cuttings, and from other experiments (i.e., by traditional methodologies of biomass quality analysis), were used for NIRS calibration. The phenotypic data are shown in Additional file 2: Table S2.

### Genotyping, quality control, and imputation

Eighteen SSR markers were used for genotyping, as described by Azevedo et al. [15]. The alleles for each marker are shown in Additional file 3: Table S3. Due to the multiallelic nature of SSR markers associated with the polyploidy of elephant grass, each allele was considered a marker (totaling 111 markers). For each marker, individuals were coded as 0 (absence of allele) or 1 (presence of allele) according to Viana et al. [47]. SSRs with more than 15% missing values (i.e., a call rate of at least 85%) and/or a frequency of minor alleles of above 1% were removed. The following imputation algorithm was used for the missing value data point in a marker matrix (*M*_*i*_): $$ {M}_i=\left\{\begin{array}{c} if:{p}_i\le 0.5\to {M}_i=0\ \\ {} if:0.5<{p}_i\le 1\to {M}_i=1\end{array}\right. $$, where *p*_*i*_ is the allele frequency associated with the presence of a marker at locus *i*. The algorithm was directly implemented as an R function. After quality control, 87 markers were used. According to previous studies [28, 29, 48, 49], the SSR markers used here are on chromosomes 1, 3, 4, 5, 6, and 7 of pearl millet.

### ssGBLUP

A mixed model methodology was adopted for statistical analyses using ssGBLUP [50, 51]. The statistical model was denoted by the following expression: *y* = *Xm* + *Za* + *Zg* + *Wb* + *Ti* + *Tr* + *Qp* + *ε*, where *y* is the vector of responses across the five cuttings (only using phenotypic data); *m* is the vector of the effects of the measurement-replication combination (assumed as fixed) added to the overall mean; *a* is the vector of genetic effects (assumed as random); *g* is the vector of residual genetic effects (assumed as random); *b* is the vector of block effects (assumed as random); *i* is the vector of the genotype by cutting interaction; *r* is the vector of the residual genotype by cutting interaction; *p* is the vector of permanent environment effects (random); *ε* is the vector of residues (random); and *X*, *Z*, *W*, *T*, and *Q* represent the incidence matrices for these effects.

The following distributions of random effects were considered: $a\sim N\left(G\bigotimes {\sigma}_a^2\right)$; $g\sim N\left(I\bigotimes {\sigma}_{rg}^2\right)$; $b\sim N\left(I\bigotimes {\sigma}_b^2\right)$; $i\sim N\left({G}_i\bigotimes {\sigma}_i^2\right)$; $r\sim N\left(I\bigotimes {\sigma}_{ri}^2\right)$; $p\sim N\left(I\bigotimes {\sigma}_p^2\right)$; and $\varepsilon \sim N\left(I\bigotimes {\sigma}_e^2\right)$, where *G* is a matrix of genomic additive relationships, *I* is an identity matrix of appropriate dimensions, *G*_*i*_ is a matrix of genomic interactions (genotype by cutting interaction), ${\sigma}_a^2$, ${\sigma}_{rg}^2$, ${\sigma}_b^2$, ${\sigma}_i^2$, ${\sigma}_{ri}^2$, ${\sigma}_p^2,$ and ${\sigma}_e^2$ are the additive, residual genetic, block, genotype by cutting interaction, residual genotype by cutting interaction, permanent environment, and residual variance components, respectively. The model above includes residual genetic effects and residual genotype by cutting interactions, according to Oakey et al. [22].

An additive relationship matrix structure (*G*) was used according to Resende et al. [24], and is denoted by the expression $G=\frac{Z^{\ast }{Z}^{\ast \prime }}{\sum_i^n{p}_i\left(1-{p}_i\right)}$, where *Z*^∗^ = *Z* − *P*, in which *Z* is a matrix containing marker genotypes and *P* is a matrix with *p*_*i*_ elements in column *i*.

Due to the presence of 10 genotypes that were not genotyped, the inverse of the genomic relationship matrix (*H*^−1^) was adopted, according to Legarra et al. [50]. The expression states that $$ {H}^{-1}={A}^{-1}+\left[\begin{array}{cc}0& 0\\ {}0& {G}^{\ast -1}-{A}_{22}^{-1}\end{array}\right] $$, where *A*^−1^ is the inverse of the pedigree relationship for all elephant grass genotypes and ${A}_{22}^{-1}$ is the inverse of the pedigree relationship for only genotyped elephant grass genotypes. *A* and *A*_22_ was an identity matrix for this study because there was no information about the pedigree. To compute the exact inverse of *G* to compose the *H*^−1^ matrix, we used the algorithm *G*^∗^ = 0.95*G* + 0.05*A*_22_ [52].

From the matrix *H*^−1^, the ssGBLUP procedure was run according to the specified model. For the random effects of the model, significance of the likelihood ratio test was evaluated using a chi-square test with one degree of freedom. ssGBLUP was performed using ASReml 4.1 software [53].

The accuracy (${r}_{\hat{a}a}$) of the additive effect considering the ssGLUP model was estimated as ${r}_{\hat{a}a}=\sqrt{1-\frac{PEV_a}{{\hat{\sigma}}_a^2}}$, where *PEV*_*a*_ is the predictive error variance that is obtained by diagonal elements inverse of the left-hand side of the mixed model equation for the additive effect.

### Simple repeatability plus genotype by cutting (G × C) interaction model

The model was run without considering the relationship (simple repeatability plus G x C interaction model) between the genotypes, as follows: *y* = *Xm* + *Zg* + *Wb* + *Ti* + *Qp* + *ε*, where *y* is the vector of response across the five cuttings (using only phenotypic data); *m* is the vector of the effects of the measurement-replication combination (assumed as fixed) added to the overall mean; *g* is the vector of genetic effects (assumed as random); *b* is the vector of block effects (assumed as random); *i* is the vector of the genotype by cutting interaction; *p* is the vector of the permanent environment effects (random); *ε* is the vector of residues (random); and *X*, *Z*, *W*, *T*, and *Q* represent the incidence matrices for these effects.

The following distributions of random effects were considered: $g\sim N\left(I\bigotimes {\sigma}_g^2\right)$; $b\sim N\left(I\bigotimes {\sigma}_b^2\right)$; $i\sim N\left({G}_i\bigotimes {\sigma}_i^2\right)$; $p\sim N\left(I\bigotimes {\sigma}_p^2\right)$; and $\varepsilon \sim N\left(I\bigotimes {\sigma}_e^2\right),$ where *I* is an identity matrix of appropriate dimensions and ${\sigma}_g^2$, ${\sigma}_b^2$, ${\sigma}_i^2$, ${\sigma}_p^2,$ and ${\sigma}_e^2$ are the genetic, block, genotype by cutting interaction, permanent environment, and residual variance components, respectively. The simple repeatability plus G × C interaction model was performed in ASReml 4.1 [53].

The accuracy (${r}_{\hat{g}g}$) of the genotype effect (genetic effects) considering the simple repeatability plus G x C interaction model was estimated as ${r}_{\hat{g}g}=\sqrt{1-\frac{PEV_g}{{\hat{\sigma}}_g^2}}$, where *PEV*_*g*_ is the predictive error variance that was obtained by the diagonal elements inverse of the left-hand side of the mixed model equation for the genetic effect.

### Genome association study

The 90 elephant grass genotypes that had been genotyped and phenotyped were used in the genome association study. The R package “sommer” (GWAS2 function [54]) revealed a significant association between the markers and the phenotypic traits. The genotype by cutting interaction was included in the association study for each cutting using the model *y* = *Xm* + *Mu* + *Za* + *Zg* + *Wb* + *ε*, where *y* is the vector of response for each cutting; *m* is the vector of the effects of the replication (assumed as fixed) added to the overall mean; *u* is the vector of markers (assumed as fixed); *a* is the vector of genetic effects (assumed as random); *g* is the vector of residual genetic effects (assumed as random); *b* is the vector of block effects (assumed as random); *ε* is the vector of residue (random); and *X*, *M*, *Z*, and *W* represent the incidence matrices for these effects.

The following distributions of random effects were considered: $a\sim N\left(G\bigotimes {\sigma}_a^2\right)$, $g\sim N\left(I\bigotimes {\sigma}_{rg}^2\right)$, $b\sim N\left(I\bigotimes {\sigma}_b^2\right),$ and $\varepsilon \sim N\left(I\bigotimes {\sigma}_e^2\right)$. In these expressions, *G* is a matrix of genomic additive relationships, *I* is an identity matrix of appropriate dimensions, and ${\sigma}_a^2$, ${\sigma}_{rg}^2$, ${\sigma}_{b,}^2$ and ${\sigma}_{\varepsilon}^2$ are the additive, residual genetic, block, and residual variances, respectively.

Markers with − log(*P* − *value*) up to the false discovery rate (FDR) threshold were considered candidate markers. To compute the FDR, a 0.02 threshold level and *p* < 0.05 were set.

We used the LD.Measures function in the R package LDcorSV to compute linkage disequilibrium (*r*^2^), as described by Hill and Robertson [55]: ${r}^2=\frac{{\left[p(AB)-p(A)p(B)\right]}^2}{p(A)p(B)\left[1-p(A)\right]\left[1-p(B)\right]}$, where *p*(*AB*) is the frequency of the haplotype AB and *p*(*A*) and *p*(*B*) are the frequencies of alleles A and B, respectively. Therefore, *r*^2^ ranged from 0 (when the two markers were in perfect equilibrium) to 1 (when the two markers provided identical information). Manhattan plots and QQ plots were obtained using the “sommer” package [54] in R [56].

### Alignment, candidate genes, and gene annotation

A list of candidate genes was assembled by BLAST, with default parameters set, using the plant comparative genomics portal Phytozome [57] based on related species (reference genomes; e.g., *S. italica*, *S. viridis*, *P. virgatum*, and *P. halli*). A BLAST search of the *C. americanus* genome was performed on the US National Center for Biotechnology Information website (https://blast.ncbi.nlm.nih.gov/Blast.cgi). For these searches, sequences of the primers M28R (CGAATACGTATGGAGAACTGCGCATC) and M35R (ATCCACCCGACGAAGGAAACGA) were used. For gene annotation, ortholog genes in *A. thaliana* were searched using the Arabidopsis Information Resource database [58].

## Supplementary information

**Additional file 1: Table S1.** Genotypes of elephant grass and their respective codes. The genotypes are part of the Active Elephant grass Germplasm Bank (BAGCE) maintained by Embrapa Gado de Leite (Embrapa Dairy Cattle).

**Additional file 2: Table S2.** Phenotyping data for the 100 elephant grass genotypes obtained on five cuttings (days 250, 500, 815, 1405 and 1615 after the uniformity cut). Abbreviations used: Cut (cutting day), Gen (genotype - see codes in Table S1), Cut.Rep (concatenation of cut and rep columns), Int (genotype by cutting interaction), Rep (replication), Height (in meters), GB (green biomass, in Mg ha-1), DB (dry biomass, in Mg ha-1), DM (dry matter concentration, in %), ADF (acid detergent fiber, in g Kg-1), NDF (neutral detergent fiber, in g Kg-1), DIG (biomass digestibility, in g Kg-1) and LIG (Lignin content, in g Kg-1).

**Additional file 3: Table S3.** Molecular data for the 90 elephant grass genotypes. “Code” indicates de code of the genotypes (see Additional file 1: Table S1).

## Data Availability

All data generated or analyzed during this study are included in this published article and in its supplementary information files. All plant materials used in this article belong to the Active Elephant Grass Germplasm Bank maintained by Embrapa Gado de Leite and are available from the corresponding author on reasonable request and signing of material transfer agreement.

## Abbreviations

ABA: Abscisic acid
ADF: Acid detergent fiber
BAGCE: Active Elephant Grass Germplasm Bank
BLAST: Basic local alignment search tool
Cut.Rep: Concatenation of cut and rep columns
DArT: Diversity arrays technology
DB: Dry biomass
DIG: Biomass digestibility
DM: Dry matter concentration
FDR: False discovery rate
G × C: Genotype by cutting interaction
GA: Genome association
GB: Green biomass
Gen: Genotype
Int: Genotype by cutting interaction
LIG: Lignin
NDF: Neutral detergent fiber
NIRS: Near-infrared spectroscopy
PE: Permanent environment
QQ plot: Quantile-quantile plot
Rep: Replication
Res G × C: Residual genotype by cutting interaction
Res G: Residual genetic
SA: Salicylic acid
SNP: Single nucleotide polymorphism
ssGBLUP: Single-step, genome-based best linear unbiased prediction
SSR: Simple sequence repeat

## References

[1] Negawo AT, Teshome A, Kumar A, Hanson J, Jones CS (2017). Opportunities for Napier grass (*Pennisetum purpureum*) improvement using molecular genetics. Agronomy..

[2] Fontoura CF, Brandão LE, Gomes LL (2015). Elephant grass biorefineries: towards a cleaner Brazilian energy matrix?. J Clean Prod.

[3] Pereira AV, Lédo FJS, Morenz MJF, Leite JLB, Santos AMB, Martins CE, Machado JC (2016). BRS Capiaçu: cultivar de capim-elefante de alto rendimento para produção de silagem.

[4] Chen XF, Huang C, Xiong L, Wang B, Qi GX, Lin XQ, Wang C, Chen XD (2016). Use of elephant grass (*Pennisetum purpureum*) acid hydrolysate for microbial oil production by *Trichosporon cutaneum*. Prep Biochem Biotechnol.

[5] Mambe FT, Voukeng IK, Beng VP, Kuete V (2016). Antibacterial activities of methanol extracts of *Alchornea cordifolia* and four other Cameroonian plants against MDR phenotypes. J Taibah Univ Med Sci.

[6] Ridzuan MJM, Majid MSA, Afendi M, Kanafiah SNA, Zahri JM, Gibson AG (2016). Characterisation of natural cellulosic fibre from *Pennisetum purpureum* stem as potential reinforcement of polymer composites. Mater Design.

[7] Zhou S, Chen J, Laib Y, Yinc G, Chena P, Pennermanc KK, Yand H, Wua B, Zhanga H, Yie X (2019). Integrative analysis of metabolome and transcriptome reveals anthocyanins biosynthesis regulation in grass species *Pennisetum purpureum*. Ind Crop Prod.

[8] Rocha JRASC, Machado JC, Carneiro PCS, Carneiro JC, Resende MDV, Lédo FJS, Carneiro JES (2017). Bioenergetic potential and genetic diversity of elephantgrass *via* morpho-agronomic and biomass quality traits. Ind Crop Prod.

[9] Reis GB, Mesquita AT, Torres GA, Andrade-Vieira LF, Pereira AV, Davide LC (2014). Genomic homeology between *Pennisetum purpureum* and *Pennisetum glaucum* (Poaceae). Comp Cytogenet.

[10] Anderson WF, Casler MD, Baldwin BS, Vermerris W (2008). Improvement of perennial forage species as feedstock for bioenergy. Genetic improvements in bioenergy crops.

[11] Zhou S, Wang C, Frazier TP, Yan H, Chen P, Chen Z, Huang L, Zhang X, Peng Y, Ma X (2018). The first Illumina-based de novo transcriptome analysis and molecular marker development in Napier grass (*Pennisetum purpureum*). Mol Breeding..

[12] Paudel D, Kannan B, Yang X, Harris-Shultz K, Thudi M, Varshney RK, Altpeter F, Wang J (2018). Surveying the genome and constructing a high-density genetic map of napiergrass (*Cenchrus purpureus* Schumach). Sci Rep.

[13] Wang C, Yan H, Li J, Zhou S, Liu T, Zhang X, Huang L (2018). Genome survey sequencing of purple elephant grass (*Pennisetum purpureum* Schum ‘Zise’) and identification of its SSR markers. Mol Breeding..

[14] Pereira JF, Azevedo ALS, Filho MP, Romanel EAC, Pereira AV, Vigna BBZ, Sobrinho FS, Benites FRG, Lédo FJS, Brito GG (2018). Research priorities for next-generation breeding of tropical forages in Brazil. Crop Breed Appl Biotechnol.

[15] Azevedo ALS, Costa PP, Machado JC, Machado MA, Pereira AV, Lédo JFS (2012). Cross-species amplification of *Pennisetum glaucum* microsatellite markers in *Pennisetum purpureum* and genetic diversity of Napier grass accessions. Crop Sci.

[16] Negawo AT, Jorge A, Hanson J, Teshome A, Muktar MS, Azevedo ALS, Lédo FLS, Machado JC, Jone CS (2018). Molecular markers as a tool for germplasm acquisition to enhance the genetic diversity of a Napier grass (*Cenchrus purpureus* syn. *Pennisetum purpureum*) collection. Trop Grassl - Forrajes Trop.

[17] Qin H, Chen M, Yi X, Bie S, Zhang C, Zhang Y, Lan J, Meng Y, Yuan Y, Jiao C (2015). Identification of associated SSR markers for yield component and fiber quality traits based on frame map and upland cotton collections. PLoS One.

[18] Gyawali S, Harrington M, Durkin J, Horner K, Parkin IAP, Hegedus DD, Bekkaoui D, Buchwaldt L (2016). Microsatellite markers used for genome-wide association mapping of partial resistance to *Sclerotinia sclerotiorum* in a world collection of *Brassica napus*. Mol Breeding..

[19] Siraree A, Banerjee N, Kumar S, Khan MS, Singh PK, Kumar S, Sharma S, Singh RK, Singh J (2017). Identification of marker-trait associations for morphological descriptors and yield component traits in sugarcane. Physiol Mol Biol Plants.

[20] Jespersen D, Ma X, Bonos SA, Belanger FC, Raymer P, Huang B (2018). Association of SSR and candidate gene markers with genetic variations in summer heat and drought performance for creeping bentgrass. Crop Sci.

[21] Biazzi E, Nazzicari N, Pecetti L, Brummer EC, Palmonari A, Tava A, Annicchiarico P (2017). Genome-wide association mapping and genomic selection for alfalfa (*Medicago sativa*) forage quality traits. PLoS One.

[22] Oakey H, Cullis B, Thompson R, Comadran J, Halpin C, Waugh R (2016). Genomic selection in multi-environment crop trials. G3 - Genes Genom Genet.

[23] Pauly M, Keegstra K (2016). Biosynthesis of the plant cell wall matrix polysaccharide xyloglucan. Annu Rev Plant Biol.

[24] Resende MDV, Silva FF, Azevedo CF (2014). Estatística matemática, biométrica e computacional: Modelos mistos, multivariados, categóricos e generalizados (REML/BLUP), inferência bayesiana, regressão aleatória, seleção genômica, QTL-GWAS, estatística espacial e temporal, competição, sobrevivência.

[25] Wang H, Li K, Hu X, Liu Z, Wu Y, Huang C (2016). Genome-wide association analysis of forage quality in maize mature stalk. BMC Plant Biol.

[26] Grev AM, Wells MS, Samac DA, Martinson KL, Sheaffer CC (2017). Forage accumulation and nutritive value of reduced lignin and reference alfalfa cultivars. Agron J.

[27] Leng P, Ouzunova M, Landbeck M, Wenzel G, Eder J, Darnhofer B, Lübberstedt T (2018). Quantitative trait loci mapping of forage Stover quality traits in six mapping populations derived from European elite maize germplasm. Plant Breed.

[28] Allouis S, Qi X, Lindup S, Gale M, Devos KM (2001). Construction of a BAC library of pearl millet, *Pennisetum glaucum*. Theor Appl Genet.

[29] Budak H, Pedraza F, Cregan PB, Baenzinger PS, Dweikat I (2003). Development and utilization of SSRs to estimate the degree of genetic relationships in a collection of pearl millet germplasm. Crop Sci.

[30] Bennetzen JL, Schmutz J, Wang H, Percifield R, Hawkins J, Pontaroli AC, Estep M, Feng L, Vaughn JN, Grimwood J (2012). Reference genome sequence of the model plant Setaria. Nat Biotechnol.

[31] Huang P, Shyu C, Coelho CP, Cao Y, Brutnell TP (2016). *Setaria viridis* as a model system to advance millet genetics and genomics. Front Plant Sci.

[32] Boerjan W, Ralph J, Baucher M (2003). Lignin biosynthesis. Annu Rev Plant Biol.

[33] Liu N, Yu P (2011). Molecular clustering, interrelationships and carbohydrate conformation in hull and seeds among barley cultivars. J Cereal Sci.

[34] Wu Z, Zhang M, Wang L, Tu Y, Zhang J, Xie G, Zou W, Li F, Guo K, Li Q (2013). Biomass digestibility is predominantly affected by three factors of wall polymer features distinctive in wheat accessions and rice mutants. Biotechnol Biofuels.

[35] Lam PY, Tobimatsu Y, Takeda Y, Suzuki S, Yamamura M, Umezawa T, Lo C (2017). Disrupting flavone synthase II alters lignin and improves biomass digestibility. Plant Physiol.

[36] Casler MD, Vogel KP (1999). Accomplishments and impact from breeding for increased forage nutritional value. Crop Sci.

[37] Fraser CM, Chapple C (2011). The phenylpropanoid pathway in Arabidopsis. Arabidopsis Book.

[38] Fu C, Mielenz JR, Xiao X, Ge Y, Hamilton CY, Rodriguez M, Chen F, Foston M, Ragauskas A, Bouton J (2011). Genetic manipulation of lignin reduces recalcitrance and improves ethanol production from switchgrass. Proc Natl Acad Sci.

[39] Mielenz JR (2001). Ethanol production from biomass: technology and commercialization status. Curr Opin Microbiol.

[40] Dorez G, Ferry L, Sonnier R, Taguet A, Lopez-Cuesta JM (2014). Effect of cellulose, hemicellulose and lignin contents on pyrolysis and combustion of natural fibers. J Anal Appl Pyrol.

[41] Zhou X, Jacobs TB, Xue LJ, Harding SA, Tsai CJ (2015). Exploiting SNP s for biallelic CRISPR mutations in the outcrossing woody perennial *Populus* reveals 4-coumarate:CoA ligase specificity and redundancy. New Phytol.

[42] Sato H, Sakamoto S, Mitsuda N, Ohme-Takagi M, Takamizo T (2018). Improvement of cell wall digestibility in tall fescue by *Oryza sativa* SECONDARY WALL NAC DOMAIN PROTEIN2 chimeric repressor. Mol Breeding.

[43] Nguyen D, Rieu I, Mariani C, van Dam NM (2016). How plants handle multiple stresses: hormonal interactions underlying responses to abiotic stress and insect herbivory. Plant Mol Biol.

[44] Wu G, Liu S, Zhao Y, Wang W, Kong Z, Tang D (2015). ENHANCED DISEASE RESISTANCE4 associates with CLATHRIN HEAVY CHAIN2 and modulates plant immunity by regulating relocation of EDR1 in Arabidopsis. Plant Cell.

[45] Goering HK. Forage fiber analysis: Apparatus, reagents, procedures and some applications, Agricultural handbook 379. Washington: U.S. Agricultural Research Service; 1970. p. 1–20.

[46] Tilley JMA, Terry RA (1963). A two-stage technique for the *in vitro* digestion of forage crops. Grass Forage Sci.

[47] Viana AP, Resende MDV, Riaz S, Walker MA (2016). Genome selection in fruit breeding: application to table grapes. Sci Agric.

[48] Mariac C, Luong V, Kapran I, Mamadou A, Sagnard F, Deu M, Chantereau J, Gerard B, Ndjeunga J, Bezançon G (2006). Diversity of wild and cultivated pearl millet accessions (*Pennisetum glaucum* [L.] R. Br.) in Niger assessed by microsatellite markers. Theor Appl Genet.

[49] Rajaram V, Nepolean T, Senthilvel S, Varshney RK, Vadez V, Srivastava RK, Shah TM, Supriya A, Kumar S, Kumari BR (2013). Pearl millet [*Pennisetum glaucum* (L.) R. Br.] consensus linkage map constructed using four RIL mapping populations and newly developed EST-SSRs. BMC Genomics.

[50] Legarra A, Aguilar I, Misztal I (2009). A relationship matrix including full pedigree and genomic information. J Dairy Sci.

[51] Aguilar I, Misztal I, Johnson DL, Legarra A, Tsuruta S, Lawlor TJ (2010). Hot topic: a unified approach to utilize phenotypic, full pedigree, and genomic information for genetic evaluation of Holstein final score. J Dairy Sci.

[52] Masuda Y, Misztal I, Tsuruta S, Legarra A, Aguilar I, Lourenco DAL, Fragomeri BO, Lawlor TJ (2016). Implementation of genomic recursions in single-step genomic best linear unbiased predictor for US Holsteins with a large number of genotyped animals. J Dairy Sci.

[53] Gilmour AR, Gogel BJ, Cullis BR, Welham SJ, Thompson R (2015). ASReml User Guide Release 4.1.

[54] Covarrubias-Pazaran G (2016). Genome-assisted prediction of quantitative traits using the R package *sommer*. PLoS One.

[55] Hill WG, Robertson A (1968). Linkage disequilibrium in finite populations. Theor Appl Genet.

[56] R Development Core Team R (2015). A language and environment for statistical computing.

[57] Goodstein DM, Shu S, Howson R, Neupane R, Hayes RD, Fazo J, Mitros T, Dirks W, Hellsten U, Putnam N (2011). Phytozome: a comparative platform for green plant genomics. Nucleic Acids Res.

[58] Reiser L, Subramaniam S, Li D, Huala E (2017). Using the *Arabidopsis* information resource (TAIR) to find information about *Arabidopsis* genes. Curr Protoc Bioinformatics.

