# Supplementary material for: Unraveling candidate genes underlying biomass digestibility in elephant grass (Cenchrus purpureus)
Source: BMC Plant Biol. 2019 Dec 10;19:548. doi: 10.1186/s12870-019-2180-5 (PMC6905061; doi:10.1186/s12870-019-2180-5)
Supplement: Supplementary file 1 — Additional file 1: Table S1. Genotypes of elephant grass and their respective codes. The genotypes are part of the Active Elephant grass Germplasm Bank (BAGCE) maintained by Embrapa Gado de Leite (Embrapa Dairy Cattle). [file 12870_2019_2180_MOESM1_ESM.pdf]

Table S1 Genotypes used in this study and their respective codes. The genotypes are part of the Active Elephant grass Germplasm Bank (BAGCE) maintained by Embrapa Gado de Leite (Embrapa Dairy Cattle).

Code	BAGCE registration	Code	BAGCE registration	Code	BAGCE registration	Code	BAGCE registration
1	Elefante da Colômbia	26	Mineiro	51	Guaco	76	12 AD IRI
2	BAGCE 2	27	Mole de Volta Grande	52	Cuba-115	77	07 AD IRI
3	Tres Rios	28	Porto Rico	53	Cuba-116	78	Pasto Panamá
4	Napier Volta Grande	29	Napier	54	Cuba-169	79	BAGCE 92
5	Mercker Santa Rita	30	Mercker Comum	55	King Grass	80	09 AD IRI
6	Pusa Napier Nº 2	31	Terezópolis	56	Roxo Botucatu	81	11 AD IRI
7	Gigante de Pinda	32	Taiwan A-26	57	Mineirão IPEACO	82	05 AD IRI
8	Napier Goiano	33	Duro de Volta Grande	58	Vruckwona Africano	83	06 AD IRI
9	Mercker S. E. A.	34	Mercker Comum Pinda	59	Cameroon	84	01 AD IRI
10	Taiwan A-148	35	Turrialba	60	BAGCE 69	85	04 AD IRI
11	Porto Rico 534-B	36	Taiwan A-146	61	Guaçu	86	13 AD IRI
12	Taiwan A-25	37	Cameroon - Piracicaba	62	Napierzinho	87	03 AD IRI
13	Albano	38	Taiwan A-121	63	IJ 7125	88	02 AD IRI
14	Híbrido Gigante da Colômbia	39	Vrukwona	64	IJ 7126	89	08 AD IRI
15	Pusa Gigante Napier	40	T241 Piracicaba	65	IJ 7127	90	Pioneiro
16	Elefante Híbrido 534-A	41	BAGCE 50	66	IJ 7136	91	Banhado
17	Costa Rica	42	BAGCE 51	67	IJ 7139	92	Roxo Farroupilha
18	Cubano de Pinda	43	Elefante Cachoeiro Itapemirim	68	IJ 7141	93	Roxo de Canguçu
19	Mercker Pinda	44	Sem Pelo	69	Goiano	94	Roxo do Itassú
20	Mercker 86 México	45	Capim Cana D'África	70	CAC 262	95	BRS Capiaçu
21	Taiwan A-144	46	Kizozi	71	Ibitinema	96	CNPGL 91-06-3
22	Napier S.E.A.	47	Gramafante	72	Australiano	97	CNPGL 96-25-3
23	Taiwan A-143	48	Roxo	73	BAGCE 82	98	BRS Canará
24	Pusa Napier Nº 1	49	Mott	74	13 AD	99	CNPGL 94-49-6
25	Elefante de Pinda	50	BAGCE 59	75	10 AD IRI	100	PCM 0701
