# Supplementary material for: Unraveling candidate genes underlying biomass digestibility in elephant grass (Cenchrus purpureus)
Source: BMC Plant Biol. 2019 Dec 10;19:548. doi: 10.1186/s12870-019-2180-5 (PMC6905061; doi:10.1186/s12870-019-2180-5)
Supplement: Supplementary file 2 — Additional file 2: Table S2. Phenotyping data for the 100 elephant grass genotypes obtained on five cuttings (days 250, 500, 815, 1405 and 1615 after the uniformity cut). Abbreviations used: Cut (cutting day), Gen (genotype - see codes in Table S1), Cut.Rep (concatenation of cut and rep columns), Int (genotype by cutting interaction), Rep (replication), Height (in meters), GB (green biomass, in Mg ha-1), DB (dry biomass, in Mg ha-1), DM (dry matter concentration, in %), ADF (acid detergent fiber, in g Kg-1), NDF (neutral detergent fiber, in g Kg-1), DIG (biomass digestibility, in g Kg-1) and LIG (Lignin content, in g Kg-1). [file 12870_2019_2180_MOESM2_ESM.pdf]

Table S2 Phenotyping data for the 100 elephant grass genotypes obtained on five cuttings (days 250, 500, 815, 1405 and 1615 after the uniformity cut). Abbreviations used: Cut (cutting day), Gen (genotype - see codes in Table S1), Cut.Rep (concatenation of cut and rep columns), int (genotype bycutting interaction), rep (replication), HDT (height, in meters), GB (green biomass, in Mg ha-1), DB (dry biomass, in Mg ha-1), DM (dry matter concentration, in %), ADF (acid detergent fiber, in g Kg-1), NDF (neutral detergent fiber, in g Kg-1), DIG (biomass digestibility, in g Kg-1), and LIG (Lignin content, in g Kg-1).

Cut	Gen	Cut.Rep	Plot	Int	Block	Rep	Height	GB	DB	DM	ADF	NDF	DIG	LIG
250	1	1	11	11	1	1	3.23	46.75	19.68	0.42	51.50	79.60	33.21	10.47
250	2	1	21	21	1	1	4.35	56.17	20.04	0.36	53.37	80.19	33.36	10.37
250	3	1	31	31	1	1	3.85	30.00	12.92	0.43	49.48	76.69	36.16	9.09
250	4	1	41	41	1	1	3.10	32.11	13.15	0.41	51.73	79.73	33.57	9.67
250	5	1	51	51	1	1	2.75	28.17	13.36	0.47	48.00	76.53	37.13	8.03
250	6	1	61	61	1	1	3.95	14.83	6.98	0.47	54.44	83.25	33.05	10.35
250	7	1	71	71	1	1	2.98	36.83	17.02	0.46	53.95	81.74	30.82	10.77
250	8	1	81	81	1	1	2.80	20.50	8.99	0.44	49.11	78.34	34.50	9.10
250	9	1	91	91	1	1	3.25	30.37	13.03	0.43	48.16	77.02	38.13	8.35
250	10	1	101	101	1	1	3.40	34.91	14.58	0.42	50.48	77.36	36.11	8.35
250	11	1	111	111	2	1	3.45	34.46	16.63	0.48	50.46	77.22	37.38	8.99
250	12	1	121	121	2	1	3.25	35.83	13.80	0.39	48.88	77.24	36.66	9.94
250	13	1	131	131	2	1	3.45	27.00	10.67	0.40	50.56	78.75	36.28	8.39
250	14	1	141	141	2	1	3.60	21.78	10.21	0.47	51.70	80.43	33.93	9.69
250	15	1	151	151	2	1	3.05	41.56	13.30	0.32	52.46	78.17	35.38	10.28
250	16	1	161	161	2	1	3.05	26.86	8.73	0.33	49.99	77.33	33.80	10.21
250	17	1	171	171	2	1	3.60	34.81	10.67	0.31	51.36	78.08	33.96	10.37
250	18	1	181	181	2	1	2.70	22.68	8.01	0.35	49.63	77.74	37.92	9.04
250	19	1	191	191	2	1	3.00	40.83	19.49	0.48	48.74	76.83	37.38	9.22
250	20	1	201	201	2	1	2.80	29.33	13.85	0.47	49.33	77.44	36.67	9.26
250	21	1	211	211	3	1	3.50	36.17	19.36	0.54	49.94	78.28	35.81	8.88
250	22	1	221	221	3	1	3.05	20.40	10.81	0.53	53.51	81.14	34.75	9.62
250	23	1	231	231	3	1	2.95	37.17	10.71	0.29	51.37	76.45	32.14	10.08
250	24	1	241	241	3	1	3.05	26.50	13.24	0.50	49.61	78.42	37.37	8.87
250	25	1	251	251	3	1	2.70	27.41	8.99	0.33	56.72	81.50	31.32	11.60
250	26	1	261	261	3	1	2.50	31.53	13.07	0.42	49.11	77.11	37.15	8.73
250	27	1	271	271	3	1	2.30	39.00	11.64	0.42	51.50	79.46	34.47	9.51
250	28	1	281	281	3	1	2.40	39.79	13.09	0.33	50.02	76.67	36.01	8.52

Table S2 Continued.

Cut	Gen	Cut.Rep	Plot	Int	Block	Rep	Height	GB	DB	DM	ADF	NDF	DIG	LIG
250	29	1	291	291	3	1	2.8	24.07	9.39	0.39	50.75	79.23	36.5	8.74
250	30	1	301	301	3	1	2.45	28.17	10.32	0.37	50.86	79.06	37.2	9.16
250	31	1	311	311	4	1	3.65	39.67	19.76	0.5	51.43	79.56	32.14	9.97
250	32	1	321	321	4	1	3.05	33.17	13.27	0.4	52.31	78.66	33.9	9.98
250	33	1	331	331	4	1	3.45	35.17	12.49	0.36	50.97	78.1	36.57	9.36
250	34	1	341	341	4	1	3	23.83	9.48	0.4	50.22	78.8	37.49	9.08
250	35	1	351	351	4	1	3.25	25.67	9.12	0.36	50.22	76.58	33.89	9.95
250	36	1	361	361	4	1	3.55	45.17	16.25	0.36	48.9	78.02	35	9.52
250	37	1	371	371	4	1	3.35	53.33	22.92	0.43	50.23	79.34	35.9	8.91
250	38	1	381	381	4	1	2.4	24.24	12.55	0.52	49.84	78.85	33.45	9.76
250	39	1	391	391	4	1	3.2	50.35	22.46	0.45	52.92	80.25	32.3	10.08
250	40	1	401	401	4	1	3.6	33.7	14.19	0.42	48.47	75.87	35.81	9.18
250	41	1	411	411	5	1	2.6	23.83	11.85	0.5	50.79	79.54	35.26	9.74
250	42	1	421	421	5	1	3.3	62.16	23.01	0.37	53.25	81.25	34.87	9.52
250	43	1	431	431	5	1	2.75	27.71	12.78	0.46	51.91	79.91	34.69	9.72
250	44	1	441	441	5	1	2.45	37.5	19.71	0.53	46.92	76.61	35.64	8.5
250	45	1	451	451	5	1	2.85	23.83	9.67	0.41	48.76	76.81	35.47	8.55
250	46	1	461	461	5	1	2.95	23.83	11.26	0.47	46.95	76.82	37.76	8.33
250	47	1	471	471	5	1	2.75	25.17	12.12	0.48	48.93	77.82	36.36	8.93
250	48	1	481	481	5	1	3.55	35.81	13	0.36	50.77	78.39	34.53	10.16
250	49	1	491	491	5	1	1.35	10.22	5.06	0.5	47.51	75.83	38.4	7.5
250	50	1	501	501	5	1	2.65	20.48	11.24	0.55	50.97	79.29	33.33	9.45
250	51	1	511	511	6	1	3.15	41.75	17.75	0.43	48.19	77.94	34.01	9.36
250	52	1	521	521	6	1	2.95	30.24	9.66	0.32	44.65	73.7	40.92	7.39
250	53	1	531	531	6	1	3.55	54.22	16.23	0.3	51.59	77.58	36.11	10.05
250	54	1	541	541	6	1	2.45	30	14.54	0.49	50.34	77.66	35.82	9.35
250	55	1	551	551	6	1	4.1	84.33	34.46	0.41	51.43	79.91	33.78	9.9
250	56	1	561	561	6	1	4.15	28.67	9.42	0.33	50.91	79.15	34.8	10.34
250	57	1	571	571	6	1	3.05	35.83	17.86	0.5	48.51	77.3	36.72	9.07
250	58	1	581	581	6	1	3.05	28.15	13.06	0.46	49.02	78.51	35.68	9.14
250	59	1	591	591	6	1	3.55	36.11	15.51	0.43	50.74	79.34	34.02	9.81

Table S2 Continued.

Cut	Gen	Cut.Rep	Plot	Int	Block	Rep	Height	GB	DB	DM	ADF	NDF	DIG	LIG
250	60	1	601	601	6	1	3	38.74	16.73	0.43	55.27	81.79	31.22	10.27
250	61	1	611	611	7	1	2.9	25.93	10.38	0.4	49.58	77.84	36.29	8.94
250	62	1	621	621	7	1	2.65	28.42	12.95	0.46	48.29	77.27	37.93	8.11
250	63	1	631	631	7	1	2.6	24.91	9.62	0.39	47.8	75.41	39.36	7.69
250	64	1	641	641	7	1	2.25	19.5	9.55	0.49	49.21	77.48	35.95	8.74
250	65	1	651	651	7	1	2.7	22.33	8.61	0.39	48.13	76.06	36.4	8.67
250	66	1	661	661	7	1	2.55	17.33	8.87	0.51	49.67	78.18	36.17	8.85
250	67	1	671	671	7	1	3	30.51	12.7	0.42	46.89	77.79	35.86	9.35
250	68	1	681	681	7	1	2.35	15	4.86	0.32	46.61	75.11	38.12	8.48
250	69	1	691	691	7	1	2.65	30	9.63	0.32	44.94	73.24	38.52	8.54
250	70	1	701	701	7	1	2.55	20.67	9.99	0.48	50.38	79.27	35.12	9.3
250	71	1	711	711	8	1	2.5	27.67	10.12	0.37	47.7	75.64	36.53	8.73
250	72	1	721	721	8	1	2.15	45.33	17.59	0.39	48.73	77.09	38.32	8.36
250	73	1	731	731	8	1	2	25.4	10.25	0.4	44.72	73.21	41.82	6.76
250	74	1	741	741	8	1	2.3	25.17	7.64	0.3	50.08	76.07	34.14	10.15
250	75	1	751	751	8	1	2.35	25.83	12.52	0.49	49.09	77.99	35.97	9.16
250	76	1	761	761	8	1	1.85	21.05	10.46	0.5	49.36	75.78	34.35	10.3
250	77	1	771	771	8	1	2.55	15.56	4.86	0.31	47.06	76.53	34.92	9.32
250	78	1	781	781	8	1	3.55	25.63	10.91	0.43	51.29	78.25	33.28	10.93
250	79	1	791	791	8	1	2.25	31.17	15.56	0.5	48.25	76.55	35.71	9.29
250	80	1	801	801	8	1	2.4	29.09	14.45	0.5	50.32	78.38	33.83	9.45
250	81	1	811	811	9	1	2.35	15.87	8.39	0.53	50.09	79.65	33.11	9.9
250	82	1	821	821	9	1	2.6	31.83	14.35	0.45	51.09	80	34.12	9.46
250	83	1	831	831	9	1	2.65	11.33	4.99	0.44	53.89	80.88	33.1	10.53
250	84	1	841	841	9	1	2.8	38.17	16.23	0.43	48.62	77.43	36.44	9.31
250	85	1	851	851	9	1	2.6	27.45	10.94	0.4	52.68	81.34	32.28	10.42
250	86	1	861	861	9	1	3.35	33.43	12	0.36	49.78	77.57	35.03	10.13
250	87	1	871	871	9	1	2.6	21.18	6.55	0.31	48.88	76.11	37.73	8.77
250	88	1	881	881	9	1	3.4	39.22	12.89	0.33	50.56	77.8	36.28	9.5
250	89	1	891	891	9	1	2.1	20.17	8.43	0.42	45.7	75.2	42.3	7.22
250	90	1	901	901	9	1	1.75	11.85	5.21	0.44	44.35	74.02	41.02	6.97

Table S2 Continued.

Cut	Gen	Cut.Rep	Plot	Int	Block	Rep	Height	GB	DB	DM	ADF	NDF	DIG	LIG
250	91	1	911	911	10	1	1.9	22.17	6.13	0.28	43.86	72.51	39.74	8.06
250	92	1	921	921	10	1	3.6	16.33	7.19	0.44	48.66	77.05	35.22	10.21
250	93	1	931	931	10	1	3.6	38.5	16.56	0.43	53.73	80.4	31.73	11.18
250	94	1	941	941	10	1	2.3	29.79	9.04	0.3	47.45	76.57	39.7	8.86
250	95	1	951	951	10	1	3.95	79.65	39.26	0.49	53.67	81.32	32.04	10.52
250	96	1	961	961	10	1	3.7	54.83	21.17	0.39	53.55	81.71	34.65	10.12
250	97	1	971	971	10	1	3.55	26.83	12.15	0.45	47.2	74.85	37.34	8.48
250	98	1	981	981	10	1	3	25.79	14.18	0.55	48.68	77.31	36.73	8.64
250	99	1	991	991	10	1	2.3	19.33	10.21	0.53	45.65	73.85	37.43	8.59
250	100	1	1001	1001	10	1	2.25	12.28	5.26	0.43	43.33	72.24	43.41	6.35
500	1	2	11	12	1	1	4	129.3	35.21	0.27	51.58	78.58	40.25	8.28
500	2	2	21	22	1	1	5.17	189.55	60.21	0.32	57.36	81.2	32.81	11.8
500	3	2	31	32	1	1	5.83	99.82	30.2	0.3	56.59	80.11	31.6	11.23
500	4	2	41	42	1	1	4.17	110.67	38.32	0.35	53.61	81.07	35.87	10.47
500	5	2	51	52	1	1	4.83	106.67	39.66	0.37	51.8	79.04	33.79	8.93
500	6	2	61	62	1	1	6	57.67	22.63	0.39	56.41	84.59	31.37	10.68
500	7	2	71	72	1	1	4.17	77.66	28.75	0.37	53.27	80.9	29.77	10.98
500	8	2	81	82	1	1	4.33	89	32.08	0.36	53.31	80.08	35.43	11
500	9	2	91	92	1	1	4.67	71.5	28.54	0.4	56.59	83.12	31.52	11.06
500	10	2	101	102	1	1	4.33	86.35	26.98	0.31	55.27	80.04	36.67	11.7
500	11	2	111	112	2	1	5.5	102.55	40.38	0.39	49.12	78.04	38.4	8.86
500	12	2	121	122	2	1	5.5	96.67	37.64	0.39	57.77	84.44	31.52	12.19
500	13	2	131	132	2	1	5.83	100.66	36.62	0.36	52.06	77.52	31.66	9.91
500	14	2	141	142	2	1	4.83	84.51	29.35	0.35	57.6	84.02	31.16	12.55
500	15	2	151	152	2	1	4	83.47	30.83	0.37	57.02	84.24	27.55	12.69
500	16	2	161	162	2	1	4.5	61.33	20.28	0.33	58.96	84.34	23.07	14.03
500	17	2	171	172	2	1	4.83	94.83	33.86	0.36	52.51	79.96	29.44	12.02
500	18	2	181	182	2	1	5.67	81.5	29.43	0.36	56.75	83.38	30.67	12.72
500	19	2	191	192	2	1	4.83	110	37.28	0.34	50.75	79.51	36.5	9.53
500	20	2	201	202	2	1	5.17	76.67	28.18	0.37	55.86	80.58	31.79	12.69
500	21	2	211	212	3	1	5.17	119.67	50.41	0.42	53.74	81.85	33.14	10.32

Table S2 Continued.

Cut	Gen	Cut.Rep	Plot	Int	Block	Rep	Height	GB	DB	DM	ADF	NDF	DIG	LIG
500	22	2	221	222	3	1	5.33	66.83	27.04	0.41	56	82.91	33.56	11.66
500	23	2	231	232	3	1	4.5	99.35	34	0.34	53.59	81.31	28.82	11.16
500	24	2	241	242	3	1	4.67	120.63	37.73	0.31	51.82	77.96	36.29	10.41
500	25	2	251	252	3	1	4	50.81	19.75	0.39	55.53	81.49	26.64	11.42
500	26	2	261	262	3	1	4.83	93.33	38.4	0.41	58.3	82.58	30.33	12.88
500	27	2	271	272	3	1	4.5	93.33	37.76	0.41	56.72	84.63	28.7	11.15
500	28	2	281	282	3	1	3.83	34.5	13.05	0.38	55.07	79.53	29.86	11.58
500	29	2	291	292	3	1	4.5	76.95	32.27	0.42	51.11	79.37	34.76	9.54
500	30	2	301	302	3	1	4.33	64.5	25.89	0.4	54.92	82.46	31.47	11.63
500	31	2	311	312	4	1	4.67	89	36.97	0.42	59.99	84.99	20.51	13.9
500	32	2	321	322	4	1	4.83	70.83	28.91	0.41	58.1	83.85	24	13.5
500	33	2	331	332	4	1	4.83	60.83	25.2	0.41	56.93	82.61	22.69	11.95
500	34	2	341	342	4	1	4.5	59.17	23.34	0.4	57.4	83.23	27.19	12.39
500	35	2	351	352	4	1	4.83	76	23.68	0.31	53.26	80.6	28.24	11.37
500	36	2	361	362	4	1	5.67	81.5	27.5	0.34	57.16	82.16	27.43	12.66
500	37	2	371	372	4	1	4.67	93.33	32.97	0.35	50.82	75.17	36.53	8.1
500	38	2	381	382	4	1	5.5	85.14	33.41	0.39	54.63	80.97	28.29	11.25
500	39	2	391	392	4	1	4	92.08	35.48	0.39	49.92	77.45	29.67	9.59
500	40	2	401	402	4	1	4.17	93.09	32.6	0.35	52.24	79.55	34.06	10.32
500	41	2	411	412	5	1	5	108.99	45.6	0.42	53.26	80.12	31.98	9.96
500	42	2	421	422	5	1	4.17	88.67	28.43	0.32	53.59	78.74	35.25	10.99
500	43	2	431	432	5	1	4.5	39.07	12.69	0.33	50.97	78.16	35.57	9.59
500	44	2	441	442	5	1	4.33	78.7	32.72	0.42	58.54	73.26	27.95	12.1
500	45	2	451	452	5	1	4.33	70.3	27.84	0.4	51.33	76.72	30.32	9.11
500	46	2	461	462	5	1	3.67	52.73	19.75	0.38	52.97	78.84	31.34	11.68
500	47	2	471	472	5	1	4.83	94.44	37.97	0.4	53.49	80.88	32.18	10.95
500	48	2	481	482	5	1	4.67	60.74	19.79	0.33	54.86	80.76	29.76	12.65
500	49	2	491	492	5	1	1.7	22.94	6.54	0.29	49.05	79.76	36.47	8.71
500	50	2	501	502	5	1	4	73.52	30.62	0.42	64.01	88.75	22.04	15.72
500	51	2	511	512	6	1	3.67	126.56	46.02	0.36	51.45	76.98	32.99	9.37
500	52	2	521	522	6	1	3.5	48.89	17.46	0.36	52.64	78.66	30.91	10.35

Table S2 Continued.

Cut	Gen	Cut.Rep	Plot	Int	Block	Rep	Height	GB	DB	DM	ADF	NDF	DIG	LIG
500	53	2	531	532	6	1	3.83	117.75	49.76	0.42	53.91	79.3	26.7	11.44
500	54	2	541	542	6	1	4.67	80	31.62	0.4	55.35	82.11	26.77	11.63
500	55	2	551	552	6	1	4.67	146	58.42	0.4	52.17	77.17	32.74	9.21
500	56	2	561	562	6	1	5	57.9	20.38	0.35	55.54	82.37	29	12.58
500	57	2	571	572	6	1	5.17	110.33	44.91	0.41	57.55	83.67	29.1	12.12
500	58	2	581	582	6	1	4.17	63.75	24.38	0.38	53.22	80.01	32.04	10.3
500	59	2	591	592	6	1	4.67	87.04	36.51	0.42	53.63	79.66	29.29	10.18
500	60	2	601	602	6	1	4.17	92.12	39.85	0.43	56.77	82.18	29.03	12.08
500	61	2	611	612	7	1	4.17	86	31.49	0.37	50.78	76.65	34.69	9.15
500	62	2	621	622	7	1	3.67	76.58	35.6	0.47	56.97	82.13	26.28	12.38
500	63	2	631	632	7	1	4.5	65.73	27.23	0.41	51.52	79.63	30.36	9.94
500	64	2	641	642	7	1	4.33	89.86	32.13	0.36	54.78	81.68	32.16	11.44
500	65	2	651	652	7	1	3.5	56.22	22.72	0.4	49.74	77.67	33.01	9.98
500	66	2	661	662	7	1	4.17	61	23.61	0.39	57.17	83.07	29.69	11.3
500	67	2	671	672	7	1	3.83	52.75	21.1	0.4	52.21	79	32.99	9.52
500	68	2	681	682	7	1	4.07	53.1	21.01	0.4	53.62	80.62	33.46	10.75
500	69	2	691	692	7	1	4.67	113.67	41.16	0.36	51.1	79.53	32.19	10.47
500	70	2	701	702	7	1	5.17	90.09	37.08	0.41	56.82	82.63	28.79	11.65
500	71	2	711	712	8	1	4.33	60.54	24.88	0.41	54.32	79.91	27.09	11.07
500	72	2	721	722	8	1	4.5	89.39	34.86	0.39	55.29	81.1	30.49	11.53
500	73	2	731	732	8	1	4.5	45.89	18.52	0.4	55.96	80.63	29.66	11.52
500	74	2	741	742	8	1	4.5	54.33	19.09	0.35	59.17	83.15	23.68	14.64
500	75	2	751	752	8	1	4.67	90.83	41.21	0.45	52.73	79.56	33.43	9.95
500	76	2	761	762	8	1	4.33	57.78	24.69	0.43	50.64	78.29	34.52	9.4
500	77	2	771	772	8	1	4	36.42	13.35	0.37	53.25	80.19	29.52	11.35
500	78	2	781	782	8	1	4.93	88.82	34.22	0.39	55.2	80.87	29.77	12.41
500	79	2	791	792	8	1	3.97	76	32.62	0.43	53.21	79.89	31.12	10.19
500	80	2	801	802	8	1	5.07	71.96	27.96	0.39	56.74	81.64	28.77	12.14
500	81	2	811	812	9	1	4.77	69	28.39	0.41	53.87	80.83	32.77	11.13
500	82	2	821	822	9	1	5.17	90.67	40.85	0.45	56.36	82.9	31.29	11.13
500	83	2	831	832	9	1	4.67	35.82	13.94	0.39	58.14	83.11	21.57	13.53

Table S2 Continued.

Cut	Gen	Cut.Rep	Plot	Int	Block	Rep	Height	GB	DB	DM	ADF	NDF	DIG	LIG
500	84	2	841	842	9	1	4.4	80.17	32.13	0.4	55.34	82.17	29.89	12.36
500	85	2	851	852	9	1	3.83	61.41	26.2	0.43	50.9	79.6	32.78	9.87
500	86	2	861	862	9	1	5	96	34.44	0.36	58.29	82.47	25.98	13.77
500	87	2	871	872	9	1	4.5	53.13	18.79	0.35	51.69	77.25	32.86	10.14
500	88	2	881	882	9	1	4.67	79.82	28.14	0.35	56.52	81.63	28.05	12.28
500	89	2	891	892	9	1	4.17	75.32	30.84	0.41	52.17	79.92	31.3	9.41
500	90	2	901	902	9	1	3.93	49.01	19.34	0.4	57.54	84.51	30.01	11.55
500	91	2	911	912	10	1	4.5	55.56	20.63	0.37	51.62	77.62	27.66	11.11
500	92	2	921	922	10	1	5.33	70.99	21.81	0.31	54.26	79.29	28.88	11.17
500	93	2	931	932	10	1	4.73	92.79	32.16	0.35	51.86	78.08	32.55	10.96
500	94	2	941	942	10	1	4.17	27.65	9.87	0.36	49.38	75.9	37.63	9.66
500	95	2	951	952	10	1	4.33	161.69	64.19	0.39	51.51	78.79	35.49	9.79
500	96	2	961	962	10	1	4.5	103	34.43	0.33	53.35	79.91	32.02	10.49
500	97	2	971	972	10	1	4.67	77.5	27.09	0.35	56.3	81.32	27.47	13.85
500	98	2	981	982	10	1	5	59.44	24.34	0.41	52.92	79.58	30.44	9.99
500	99	2	991	992	10	1	4.17	76.47	30.91	0.4	54.58	81.15	27.85	13.07
500	100	2	1001	1002	10	1	4.17	49.41	14.79	0.3	48.45	74.28	37.3	8.28
815	1	3	11	13	1	1	3.53	73.63	30.63	0.42	51.28	80.53	39.25	8.93
815	2	3	21	23	1	1	3.97	127.55	45.92	0.36	52.18	80.98	35.59	10.32
815	3	3	31	33	1	1	3.6	92.57	29.95	0.32	51.98	80.71	35.7	10.36
815	4	3	41	43	1	1	3.27	82.42	31.54	0.38	50.19	80.08	40.8	9.16
815	5	3	51	53	1	1	3.17	61.71	21.46	0.35	52.97	81.98	41.75	9.36
815	6	3	61	63	1	1	3.77	32.12	11.25	0.35	53.52	84.7	36.69	8.54
815	7	3	71	73	1	1	3.53	84.58	32.04	0.38	50.56	81.06	36.53	8.87
815	8	3	81	83	1	1	3.17	28.19	11.69	0.41	53.13	81.58	33.38	10.65
815	9	3	91	93	1	1	3.1	75.96	31.9	0.42	54.23	83.64	33.34	11.09
815	10	3	101	103	1	1	3.23	75.11	23.37	0.31	51.02	77.55	40.3	9.74
815	11	3	111	113	2	1	3.43	81.03	21.14	0.26	54.16	82.35	42.26	8.46
815	12	3	121	123	2	1	3.3	56.99	20.36	0.36	50.96	81.34	38.28	9.51
815	13	3	131	133	2	1	4.17	53.74	14.72	0.27	51.31	78.83	38.27	8.78
815	14	3	141	143	2	1	3.03	48.95	14.98	0.31	48.58	78.24	41.88	8.34

Table S2 Continued.

Cut	Gen	Cut.Rep	Plot	Int	Block	Rep	Height	GB	DB	DM	ADF	NDF	DIG	LIG
815	15	3	151	153	2	1	3.37	42.92	14.81	0.34	49.89	78.56	35.98	9.8
815	16	3	161	163	2	1	3.7	48.38	15.27	0.32	51.11	79.9	34.23	10.34
815	17	3	171	173	2	1	3.87	55.56	15.54	0.28	51.42	79.31	29.97	10.14
815	18	3	181	183	2	1	3.57	99.8	31.43	0.31	53.42	81.77	34.34	9.52
815	19	3	191	193	2	1	3.47	89.71	26.3	0.29	52.29	78.89	34.23	9.32
815	20	3	201	203	2	1	3.43	49.26	14.57	0.3	52.68	79.81	36.51	9.03
815	21	3	211	213	3	1	3.83	110.48	32.88	0.3	51.12	80.55	41.22	8.46
815	22	3	221	223	3	1	3.9	73.57	24.01	0.33	53.25	82.02	33.07	9.74
815	23	3	231	233	3	1	3.6	48.89	14.13	0.29	52.19	79.26	32.72	10.71
815	24	3	241	243	3	1	3.5	77.33	23.75	0.31	48.8	77.78	34.37	8.82
815	25	3	251	253	3	1	3.33	36.76	13.05	0.35	53.28	81	27.98	10.59
815	26	3	261	263	3	1	3.27	59.05	20.51	0.35	51.07	79.22	35.85	9.36
815	27	3	271	273	3	1	3.17	55.81	22.02	0.39	50.56	78.28	35.3	9.01
815	28	3	281	283	3	1	3.07	27.88	10.23	0.37	49.71	78.5	35.48	8.77
815	29	3	291	293	3	1	3.37	70.86	24.25	0.34	49.19	78.3	39.24	8.49
815	30	3	301	303	3	1	3.37	24.57	8.73	0.36	50.74	78.63	35.06	9.34
815	31	3	311	313	4	1	3.03	55	17.09	0.31	51.38	78.08	36.22	9.85
815	32	3	321	323	4	1	3.63	57.14	12.71	0.22	51.61	78.31	29.58	10.1
815	33	3	331	333	4	1	3.63	63.05	23.44	0.37	48.66	76.2	34.21	9.16
815	34	3	341	343	4	1	3.23	30.1	10.19	0.34	48.89	77.7	36.41	8.13
815	35	3	351	353	4	1	3.7	36.36	10.46	0.29	53.57	80.21	30.34	10.78
815	36	3	361	363	4	1	4.07	68.95	19.45	0.28	49.68	77.29	35.13	8.63
815	37	3	371	373	4	1	3.4	65.33	19.02	0.29	48.78	77.32	40.96	8.21
815	38	3	381	383	4	1	3.4	52.9	17.72	0.34	51.32	79.75	34.2	9.18
815	39	3	391	393	4	1	3.43	46.21	17.73	0.38	51.87	81.45	32.39	9.8
815	40	3	401	403	4	1	3.58	68.41	19.41	0.28	46.74	76.9	48.81	7.38
815	41	3	411	413	5	1	3.4	74.67	25.83	0.35	52.47	80.87	34.32	9.93
815	42	3	421	423	5	1	3.33	56.53	21.55	0.38	47.65	78.36	37.65	8.5
815	43	3	431	433	5	1	3.17	52.11	19.45	0.37	48.58	77.06	36.62	8.28
815	44	3	441	443	5	1	3.43	108.23	41.69	0.39	51.02	80.41	36.59	9.42
815	45	3	451	453	5	1	3.3	51.83	19.28	0.37	50.21	78.55	36.62	9.39

Table S2 Continued.

Cut	Gen	Cut.Rep	Plot	Int	Block	Rep	Height	GB	DB	DM	ADF	NDF	DIG	LIG
815	46	3	461	463	5	1	3.53	41.08	14.53	0.35	51.04	79.31	33.72	8.64
815	47	3	471	473	5	1	3.4	86.26	30.47	0.35	49.5	78.58	34.75	8.6
815	48	3	481	483	5	1	4.03	58.53	16.48	0.28	46.15	74.54	36.22	7.9
815	49	3	491	493	5	1	1.77	21.96	5.87	0.27	45.07	75.21	41.85	6.74
815	50	3	501	503	5	1	3.37	77.39	27.56	0.36	52.97	78.47	32.41	9.24
815	51	3	511	513	6	1	3.4	102	32.06	0.31	44.91	73.96	41.51	6.88
815	52	3	521	523	6	1	3.1	55.21	18.22	0.33	45.75	74.64	37.75	8.09
815	53	3	531	533	6	1	3.57	62.86	22.32	0.36	50.02	78.32	36.32	9.17
815	54	3	541	543	6	1	3.17	62.14	23.98	0.39	48.55	78.08	35.49	8.47
815	55	3	551	553	6	1	3.53	72	26.39	0.37	51.95	78.57	66.66	8.85
815	56	3	561	563	6	1	3.33	27.59	8.52	0.31	48.47	76.89	36.79	9.17
815	57	3	571	573	6	1	3.43	60.38	21.5	0.36	50.27	78.54	42.88	8.5
815	58	3	581	583	6	1	3.3	36.22	12.5	0.35	46.18	74.46	45.07	7.05
815	59	3	591	593	6	1	3.4	57.63	21.98	0.38	49.29	78.65	35.75	8.88
815	60	3	601	603	6	1	3.47	49.58	18.32	0.37	47.12	76.79	41.49	8.37
815	61	3	611	613	7	1	3.5	49.03	16.12	0.33	47.91	76.29	40.49	7.88
815	62	3	621	623	7	1	3.23	52.32	21.69	0.41	50.01	78.64	30.4	8.57
815	63	3	631	633	7	1	3.43	56	20.39	0.36	48.42	76.68	44.7	8.12
815	64	3	641	643	7	1	3.37	46.67	15.11	0.32	45.38	73.48	44.32	6.96
815	65	3	651	653	7	1	3.2	38.33	12.28	0.32	47.29	75.73	42.03	8.37
815	66	3	661	663	7	1	3.4	54.48	20.89	0.38	47.92	77.05	39.47	8.42
815	67	3	671	673	7	1	3.2	52.1	19.64	0.38	51.11	81.1	35.7	9.32
815	68	3	681	683	7	1	3.53	39.13	12.69	0.32	48.66	77.04	35.89	9.92
815	69	3	691	693	7	1	3.5	42.86	13.42	0.31	49.22	77.27	36.16	9.63
815	70	3	701	703	7	1	3.7	92	33.63	0.37	51.33	79.35	35.78	9.22
815	71	3	711	713	8	1	2.97	29.52	9.89	0.34	51.45	79.84	35.57	9.97
815	72	3	721	723	8	1	3.33	90.29	34.37	0.38	52.23	79.79	37.17	8.77
815	73	3	731	733	8	1	3.23	105.03	37.36	0.36	46.89	75.81	42.92	8.09
815	74	3	741	743	8	1	3.93	30.86	9.47	0.31	48.1	74.73	40.98	8.79
815	75	3	751	753	8	1	3.4	92.19	32.46	0.35	50.51	78.68	36.68	9.09
815	76	3	761	763	8	1	3.6	50.42	17.67	0.35	47.47	77.01	38.53	7.74

Table S2 Continued.

Cut	Gen	Cut.Rep	Plot	Int	Block	Rep	Height	GB	DB	DM	ADF	NDF	DIG	LIG
815	77	3	771	773	8	1	3.43	26.45	9.65	0.37	46.75	75.09	36.7	8.62
815	78	3	781	783	8	1	3.3	68	21.7	0.32	48.51	76.14	36.21	8.96
815	79	3	791	793	8	1	3.1	41.14	14.99	0.36	48.3	77.94	37.26	8.66
815	80	3	801	803	8	1	3.23	73.74	26.86	0.36	47.83	75.43	37.8	8.36
815	81	3	811	813	9	1	3.23	36.46	13.87	0.38	49.71	77.93	32.82	9.07
815	82	3	821	823	9	1	3.37	73.52	27.11	0.37	47.41	77.13	37.91	7.28
815	83	3	831	833	9	1	3.43	57.24	21.31	0.37	51.93	80.89	31.08	9.93
815	84	3	841	843	9	1	3.37	62.36	24.26	0.39	51.33	80.1	31.22	9.44
815	85	3	851	853	9	1	3.2	50.32	19.62	0.39	53.08	81.05	31.41	9.61
815	86	3	861	863	9	1	3.6	80	23.51	0.29	49.47	78.61	35.97	8.62
815	87	3	871	873	9	1	3.57	53.82	17.66	0.33	51.21	78.34	32.1	9.74
815	88	3	881	883	9	1	3.43	58.79	17.76	0.3	48.28	75.72	35.23	8.46
815	89	3	891	893	9	1	3.1	53.96	20.97	0.39	49.91	77.25	33.72	8.44
815	90	3	901	903	9	1	3	29.9	11.75	0.39	48.93	77.53	37.55	8.57
815	91	3	911	913	10	1	3.73	28.19	9.13	0.32	52.01	78.99	32.09	10.48
815	92	3	921	923	10	1	4.03	44.24	13.34	0.3	44.73	73.46	42.25	7.15
815	93	3	931	933	10	1	3.43	60.23	20.48	0.34	47.64	74.93	39.35	7.74
815	94	3	941	943	10	1	3.4	30.56	10.39	0.34	46.52	75.48	40.16	8.67
815	95	3	951	953	10	1	3.9	119.81	36.4	0.3	50.37	79.51	37.92	8.91
815	96	3	961	963	10	1	3.63	89.25	30.25	0.34	50.44	79.33	41.81	8.85
815	97	3	971	973	10	1	3.77	62.67	19.44	0.31	46.43	74.31	42.41	8.42
815	98	3	981	983	10	1	3.17	49.07	18.15	0.37	49	77.94	39.93	8.53
815	99	3	991	993	10	1	2.9	24.76	7.72	0.31	45.82	75.44	44.82	7.56
815	100	3	1001	1003	10	1	3.17	24.26	7	0.29	45.02	72.88	43.38	6.28
1405	1	4	11	14	1	1	2.6	75	23.18	0.31	49.03	77.89	41.69	8.16
1405	2	4	21	24	1	1	2.8	112	34.09	0.3	46.94	79.26	40.82	8.9
1405	3	4	31	34	1	1	2.7	84.89	27.14	0.32	52	79.99	36.89	10.55
1405	4	4	41	44	1	1	2.6	71.11	17.24	0.24	52.23	81.94	41	9.32
1405	5	4	51	54	1	1	2.7	49.33	19.04	0.39	45.99	78.96	43.79	7.46
1405	6	4	61	64	1	1	2.5	18.22	3.31	0.18	50.65	81.45	43.9	8.18
1405	7	4	71	74	1	1	2.4	56.44	21.83	0.39	52.07	81.13	33.72	9.49

Table S2 Continued.

Cut	Gen	Cut.Rep	Plot	Int	Block	Rep	Height	GB	DB	DM	ADF	NDF	DIG	LIG
1405	8	4	81	84	1	1	2.3	15.11	5.65	0.37	47.38	79.41	40.5	8.43
1405	9	4	91	94	1	1	2.4	25.78	9.52	0.37	46.78	78.26	42.04	7.68
1405	10	4	101	104	1	1	2.6	44.89	13.61	0.3	47.79	76.63	41.12	8.02
1405	11	4	111	114	2	1	2.7	67.11	25	0.37	48.29	79.23	39.3	8.82
1405	12	4	121	124	2	1	2.5	56.89	22.2	0.39	50.02	81.95	39.43	8.86
1405	13	4	131	134	2	1	2.8	39.56	16.38	0.41	52.07	81.65	33.91	10.4
1405	14	4	141	144	2	1	2.6	30.44	12.98	0.43	49	78.76	38.83	8.81
1405	15	4	151	154	2	1	2.9	35.56	9.32	0.26	48.48	77.89	44.03	8.02
1405	16	4	161	164	2	1	2.5	53.11	13.63	0.26	48.12	76.71	40.24	9.26
1405	17	4	171	174	2	1	2.6	48.44	12.67	0.26	49.74	77.51	40.55	9.24
1405	18	4	181	184	2	1	2.8	86.22	25.93	0.3	48.38	77.33	37.08	9.78
1405	19	4	191	194	2	1	2.5	58.22	19.58	0.34	47.08	76.15	41.56	8.31
1405	20	4	201	204	2	1	2.4	38.67	12.76	0.33	45.65	74.13	41.33	8.47
1405	21	4	211	214	3	1	3	87.56	33.66	0.38	51.58	79.79	37.05	10.34
1405	22	4	221	224	3	1	2.9	40.44	16.29	0.4	51.61	80.9	36.84	10.18
1405	23	4	231	234	3	1	2.2	36	10.24	0.28	51.09	76.71	37	9.6
1405	24	4	241	244	3	1	2.5	41.33	15.44	0.37	49.3	78.99	39.27	9.48
1405	25	4	251	254	3	1	2.5	31.78	13.18	0.41	52.86	81.77	34.34	11.58
1405	26	4	261	264	3	1	2.4	71.11	22.66	0.32	49.71	80.6	36.42	9.69
1405	27	4	271	274	3	1	2.5	64	26.08	0.41	47.04	78.62	39.01	8.4
1405	28	4	281	284	3	1	2.4	10.22	3.53	0.34	47.86	77.46	40.9	8.14
1405	29	4	291	294	3	1	2.9	68.44	25.69	0.38	46.82	76.04	40.15	8
1405	30	4	301	304	3	1	2.3	11.11	4.42	0.4	49.49	76.78	38.74	9.05
1405	31	4	311	314	4	1	2.6	40.44	13.64	0.34	49.03	77.11	37.15	9.08
1405	32	4	321	324	4	1	2.8	45.11	15.1	0.33	49	77.5	38.38	8.71
1405	33	4	331	334	4	1	2.6	68.89	23.28	0.34	47	74.83	39.57	8.88
1405	34	4	341	344	4	1	2.4	20.89	8.01	0.38	47.27	77.51	40.17	8.65
1405	35	4	351	354	4	1	2.3	21.11	6.02	0.28	47.65	77.28	38.78	8.86
1405	36	4	361	364	4	1	2.7	53.33	14.42	0.27	48.13	77.85	41.05	8.26
1405	37	4	371	374	4	1	2.2	36.89	13.61	0.37	44.3	77.02	43.71	6.5
1405	38	4	381	384	4	1	2.6	36	15.15	0.42	45.67	75.65	38.65	8.26

Table S2 Continued.

Cut	Gen	Cut.Rep	Plot	Int	Block	Rep	Height	GB	DB	DM	ADF	NDF	DIG	LIG
1405	39	4	391	394	4	1	2.4	23.56	8.24	0.35	50.18	80.19	38.68	9.48
1405	40	4	401	404	4	1	2.5	68	24.51	0.36	48.42	79.71	40.34	8.92
1405	41	4	411	414	5	1	2.6	58.89	24.4	0.41	49.98	79.78	38.04	9.9
1405	42	4	421	424	5	1	3	77.11	20.86	0.27	48.7	80.2	40.82	9.58
1405	43	4	431	434	5	1	2.5	70.89	28.28	0.4	49.11	77.99	39.32	9.56
1405	44	4	441	444	5	1	2.8	103.55	40.04	0.39	48.91	81.32	41.54	8.86
1405	45	4	451	454	5	1	2.7	39.78	13.64	0.34	50.12	79.63	38.8	9.28
1405	46	4	461	464	5	1	2.4	20	7.2	0.36	50.86	82.41	37.6	10.02
1405	47	4	471	474	5	1	2.7	79.11	36.59	0.37	47.73	78.87	40.79	8.82
1405	48	4	481	484	5	1	3	27.56	6.36	0.23	50.53	78.97	41.17	8.83
1405	49	4	491	494	5	1	NA	NA	NA	0.35	49.35	76.97	37.85	9.22
1405	50	4	501	504	5	1	2.6	47.33	17.1	0.36	48.6	77.35	39.35	8.9
1405	51	4	511	514	6	1	2.9	70	16.22	0.38	49.58	79.38	39.6	8.88
1405	52	4	521	524	6	1	2.4	24.44	8.24	0.34	47.41	78.31	40.88	8.83
1405	53	4	531	534	6	1	2.3	54.89	20.05	0.37	48.26	78.74	38.77	8.87
1405	54	4	541	544	6	1	3	36	15.28	0.42	46.78	78.78	40.07	8.57
1405	55	4	551	554	6	1	2.7	48.67	17.47	0.36	48.67	78.45	39.01	8.8
1405	56	4	561	564	6	1	2.3	10	3.58	0.36	46.69	76.98	39.63	8.57
1405	57	4	571	574	6	1	2.4	44.44	17.58	0.4	47.17	77.1	38.94	8.77
1405	58	4	581	584	6	1	2.7	38	13.55	0.36	45.61	76.33	42.04	7.64
1405	59	4	591	594	6	1	2.9	33.33	11.33	0.34	50.22	80.3	39.78	9.2
1405	60	4	601	604	6	1	2.7	27.78	10.5	0.38	NA	NA	NA	NA
1405	61	4	611	614	7	1	3.3	53.33	17.29	0.32	46.42	77.74	41.45	7.7
1405	62	4	621	624	7	1	2.4	42.22	11.33	0.27	46.81	77.94	42.55	8.85
1405	63	4	631	634	7	1	2.7	46.44	18.4	0.4	45.56	74.86	40.92	7.67
1405	64	4	641	644	7	1	2.3	28.89	10.86	0.38	45.81	73.8	42.18	7.22
1405	65	4	651	654	7	1	2.4	33.33	10.51	0.32	47.07	77.34	40.49	8.65
1405	66	4	661	664	7	1	2.4	37.78	14.36	0.38	45.65	79.32	41.73	8.5
1405	67	4	671	674	7	1	2.6	25.78	7.1	0.28	51.3	79.38	37.86	9.94
1405	68	4	681	684	7	1	2.8	36.67	14.26	0.39	44.1	76.28	46.87	7.19
1405	69	4	691	694	7	1	2.1	30.67	8.81	0.29	46.49	77.42	42.15	8.69

Table S2 Continued.

Cut	Gen	Cut.Rep	Plot	Int	Block	Rep	Height	GB	DB	DM	ADF	NDF	DIG	LIG
1405	70	4	701	704	7	1	2.8	76	30.81	0.41	46.67	79.4	40.74	7.83
1405	71	4	711	714	8	1	2.3	25.78	9.27	0.36	50.77	81.32	38.82	8.92
1405	72	4	721	724	8	1	3	108.89	36.87	0.34	48.06	78.2	46.12	7.33
1405	73	4	731	734	8	1	2.8	76.22	24.8	0.33	23.52	58.71	52.46	6.62
1405	74	4	741	744	8	1	2.5	6.67	1.91	0.29	50.72	77.79	37.43	9.94
1405	75	4	751	754	8	1	2.8	73.78	30.17	0.41	47.67	79.76	39.28	8.91
1405	76	4	761	764	8	1	2.2	44.44	18.35	0.41	49.15	79.45	38.95	9.2
1405	77	4	771	774	8	1	1.8	6.67	2.37	0.36	51.33	81.21	42.32	8.56
1405	78	4	781	784	8	1	2.8	63.78	20.59	0.32	49.37	78.74	38.87	9.25
1405	79	4	791	794	8	1	2.4	40	13.79	0.34	46.11	76.19	42.2	7.87
1405	80	4	801	804	8	1	2.3	38.22	15.23	0.4	47.52	77.7	38.91	8.73
1405	81	4	811	814	9	1	2.4	40	12.23	0.31	50.27	79.81	40.09	8.56
1405	82	4	821	824	9	1	2.4	64.89	24.35	0.38	49.11	80.35	42.01	8.31
1405	83	4	831	834	9	1	2.5	32	10.7	0.33	50.88	78.08	37.48	9.3
1405	84	4	841	844	9	1	2.8	37.33	14.29	0.38	48.94	80.31	39.32	8.77
1405	85	4	851	854	9	1	2.9	39.56	15.51	0.39	46.09	77.42	39.55	8.18
1405	86	4	861	864	9	1	2.8	52.89	16.37	0.31	50.15	77.37	38.03	9.17
1405	87	4	871	874	9	1	3.4	21.78	6.73	0.31	50.54	79.13	39.7	8.94
1405	88	4	881	884	9	1	3.6	28.44	9.07	0.32	54.07	80.11	36.17	10.11
1405	89	4	891	894	9	1	2.3	38.22	14.32	0.37	47.9	77.13	37.21	8.69
1405	90	4	901	904	9	1	2	30.22	12.65	0.42	45.76	74.24	38.13	7.05
1405	91	4	911	914	10	1	2.5	33.78	9.6	0.28	50.48	77.11	36.75	9.8
1405	92	4	921	924	10	1	2.8	34.67	12.24	0.35	47.25	75.01	38.21	8.32
1405	93	4	931	934	10	1	3.2	33.56	9.94	0.3	51.57	77.93	38.2	9.79
1405	94	4	941	944	10	1	3.1	16	4.99	0.31	NA	NA	NA	NA
1405	95	4	951	954	10	1	2.9	57.78	18.49	0.32	44.28	74.63	43.69	7.06
1405	96	4	961	964	10	1	2.9	47.56	19	0.4	52.79	80.97	36.38	9.54
1405	97	4	971	974	10	1	2.8	52.89	18.04	0.34	50.06	76.93	36.42	9.43
1405	98	4	981	984	10	1	2.5	10.67	4.5	0.42	47.5	74.55	41.62	8.18
1405	99	4	991	994	10	1	0	0	0	0	0	0	0	0
1405	100	4	1001	1004	10	1	0	0	0	0	43.32	75.85	45.62	6.47

Table S2 Continued.

Cut	Gen	Cut.Rep	Plot	Int	Block	Rep	Height	GB	DB	DM	ADF	NDF	DIG	LIG
1615	1	5	11	15	1	1	4	112.397	38.925	0.346	40.98	77.28	43.91	7.84
1615	2	5	21	25	1	1	4.2	113.645	40.554	0.357	44.32	78.15	37.84	9.1
1615	3	5	31	35	1	1	4.1	108.889	28.257	0.26	44.88	77.66	40.62	8.72
1615	4	5	41	45	1	1	3.8	76.531	18.951	0.248	46.42	78.74	46.98	7.63
1615	5	5	51	55	1	1	3.2	55.111	20.547	0.373	41.22	78.32	41.5	7.37
1615	6	5	61	65	1	1	4	20.408	7.172	0.351	45.68	81.16	41.52	7.63
1615	7	5	71	75	1	1	3	56.818	19.76	0.348	47.02	81.17	39.22	9.47
1615	8	5	81	85	1	1	2.8	33.778	12.426	0.368	46.92	79.11	39.87	8.24
1615	9	5	91	95	1	1	3.1	24.49	9.423	0.385	51.05	82.92	38.51	9.95
1615	10	5	101	105	1	1	3.5	27.2	9.02	0.332	46.17	78.01	40.06	9.64
1615	11	5	111	115	2	1	4.2	67.174	25.963	0.387	41.78	76.49	45.17	8.24
1615	12	5	121	125	2	1	3.4	64.497	22.465	0.348	45.24	79.88	40.18	9.21
1615	13	5	131	135	2	1	4.3	50.51	18.118	0.359	42.41	77.48	41.91	8.84
1615	14	5	141	145	2	1	3.5	35.111	11.571	0.33	46.81	79.48	38.96	8.92
1615	15	5	151	155	2	1	3.3	31.276	10.459	0.334	46.21	78.99	38.29	10.25
1615	16	5	161	165	2	1	3.4	55.556	17.934	0.323	46.32	78.12	40.83	9.64
1615	17	5	171	175	2	1	3.3	58.88	20.828	0.354	42.26	76.77	44.05	8.51
1615	18	5	181	185	2	1	4.8	81.837	29.043	0.355	41.92	77.08	42.65	8.87
1615	19	5	191	195	2	1	3.8	68	25.043	0.368	42.29	75.98	39.61	9
1615	20	5	201	205	2	1	4.6	49.778	17.173	0.345	42.01	75.51	39.65	9.64
1615	21	5	211	215	3	1	4.2	72	27.79	0.386	47.07	79.97	38.99	9.13
1615	22	5	221	225	3	1	3.8	64.518	22.657	0.351	46.68	78.77	37.79	8.94
1615	23	5	231	235	3	1	3.1	28.571	9.136	0.32	47.59	78.34	38.05	10.14
1615	24	5	241	245	3	1	3.2	60.444	21.213	0.351	42.37	75.01	43.4	7.93
1615	25	5	251	255	3	1	3	30	9.843	0.328	45.11	77.52	42.16	9.25
1615	26	5	261	265	3	1	3.8	60.889	23.081	0.379	46.58	79.81	39.4	9.08
1615	27	5	271	275	3	1	3.2	55.556	21.039	0.379	46.07	78.15	42.02	8.77
1615	28	5	281	285	3	1	3.2	25.51	NA	NA	NA	NA	NA	NA
1615	29	5	291	295	3	1	3.5	63.111	24.621	0.39	43.85	79.32	39.01	9.53
1615	30	5	301	305	3	1	3	36.222	14.879	0.411	47.34	82.53	34.18	10.54
1615	31	5	311	315	4	1	3.8	50.48	20.246	0.401	45.31	79.06	37.93	9.34

Table S2 Continued.

Cut	Gen	Cut.Rep	Plot	Int	Block	Rep	Height	GB	DB	DM	ADF	NDF	DIG	LIG
1615	32	5	321	325	4	1	3.6	45.778	19.575	0.428	48.17	82.08	32.49	11.06
1615	33	5	331	335	4	1	3.6	47.333	20.412	0.431	46.21	80.64	35.11	10.75
1615	34	5	341	345	4	1	3.2	26.667	11.817	0.443	47.98	81.71	34.68	11.53
1615	35	5	351	355	4	1	3.3	39.286	14.702	0.374	42.14	78.99	39.32	9.94
1615	36	5	361	365	4	1	3.5	69.778	26.515	0.38	44.86	78.47	41.5	8.6
1615	37	5	371	375	4	1	3.1	54.4	19.118	0.351	42.71	76.28	43.38	7.93
1615	38	5	381	385	4	1	3.6	80.799	32.532	0.403	44.46	78.89	40.28	10.03
1615	39	5	391	395	4	1	3	61.806	24.152	0.391	41.55	78.48	41.74	8.65
1615	40	5	401	405	4	1	3.3	106.173	35.318	0.333	42.35	76.47	42.94	7.99
1615	41	5	411	415	5	1	3.6	60	24.214	0.404	43.96	78.37	40.18	10.28
1615	42	5	421	425	5	1	3.3	113	44.877	0.397	39.43	75.85	43.14	8.43
1615	43	5	431	435	5	1	3.6	84.444	31.027	0.367	40.7	75.68	42.21	8.96
1615	44	5	441	445	5	1	4	70.222	27.095	0.386	49.07	82.44	33.01	11.88
1615	45	5	451	455	5	1	4	65.089	23.746	0.365	45.69	77.41	37.75	9.83
1615	46	5	461	465	5	1	NA	NA	NA	NA	NA	NA	NA	NA
1615	47	5	471	475	5	1	3.6	92.755	40.332	0.435	47.63	80.84	39.96	10.07
1615	48	5	481	485	5	1	4	84.298	28.609	0.339	47.05	78.87	37.82	10.43
1615	49	5	491	495	5	1	NA	NA	NA	NA	NA	NA	NA	NA
1615	50	5	501	505	5	1	3.4	112.963	42.844	0.379	46.55	82.26	35.31	11.7
1615	51	5	511	515	6	1	3.2	92.4	32.358	0.35	44.1	78.97	40.9	8.58
1615	52	5	521	525	6	1	2.8	46.365	14.574	0.314	45.48	78.58	37.59	10.15
1615	53	5	531	535	6	1	3.5	61.778	24.015	0.389	40.65	78.28	35.44	10.3
1615	54	5	541	545	6	1	3.8	81.248	30.081	0.37	46.07	81.04	38.04	10.11
1615	55	5	551	555	6	1	3.2	68.444	24.146	0.353	40.83	77.21	40.05	8.44
1615	56	5	561	565	6	1	3.2	45.833	14.614	0.319	46.43	80.59	38.35	10.13
1615	57	5	571	575	6	1	3.6	59.556	24.919	0.418	45.18	80.56	38.48	9.76
1615	58	5	581	585	6	1	3	NA	NA	0.316	37.91	75	45.88	7.23
1615	59	5	591	595	6	1	3.2	36.651	12.918	0.352	46.14	78.9	38.06	9.76
1615	60	5	601	605	6	1	2.9	28.532	10.819	0.379	41.05	78.3	41.15	8.6
1615	61	5	611	615	7	1	3.5	59.172	19.791	0.334	41.99	76.94	40.99	8.26
1615	62	5	621	625	7	1	3	35.714	13.694	0.383	46.47	81.74	36.93	9.5

Table S2 Continued.

Cut	Gen	Cut.Rep	Plot	Int	Block	Rep	Height	GB	DB	DM	ADF	NDF	DIG	LIG
1615	63	5	631	635	7	1	3.3	36	14.066	0.391	46.89	79.59	39.53	9.59
1615	64	5	641	645	7	1	3.5	66.327	23.236	0.35	46.62	78.36	42.41	8.77
1615	65	5	651	655	7	1	2.8	38.409	12.884	0.335	43.38	77.7	38.88	9.52
1615	66	5	661	665	7	1	3.4	62.444	24.702	0.396	41.28	79.29	41.83	8.65
1615	67	5	671	675	7	1	2.7	51.405	19.514	0.38	42.83	79.83	40.62	8.54
1615	68	5	681	685	7	1	3.3	108.025	35	0.324	42.88	79.4	40.14	9.85
1615	69	5	691	695	7	1	3.2	38.222	13.853	0.362	47.81	80.54	36.5	11
1615	70	5	701	705	7	1	3.7	62	21.288	0.343	43.31	77.4	40.95	9.72
1615	71	5	711	715	8	1	3	20	7.763	0.388	48.29	80.63	39.31	10.27
1615	72	5	721	725	8	1	3.4	60.889	22.271	0.366	42.23	77.1	39.6	9.55
1615	73	5	731	735	8	1	3.4	76.889	29.477	0.383	43.98	77.84	40.03	9.72
1615	74	5	741	745	8	1	NA	NA	NA	NA	NA	NA	NA	NA
1615	75	5	751	755	8	1	3.4	80.889	34.585	0.428	48.38	80.78	37.24	10.35
1615	76	5	761	765	8	1	3.2	64.472	24.684	0.383	43.75	78.33	40.47	8.51
1615	77	5	771	775	8	1	NA	NA	NA	NA	NA	NA	NA	NA
1615	78	5	781	785	8	1	3.6	63.556	21.858	0.344	43.72	78.3	38.66	10.34
1615	79	5	791	795	8	1	2.3	35.111	14.439	0.411	46.03	78.84	35.81	10.2
1615	80	5	801	805	8	1	3.2	48.469	15.822	0.326	45.45	78.75	41.62	9.61
1615	81	5	811	815	9	1	3.3	37.037	12.882	0.348	42.02	76.79	43.84	7.71
1615	82	5	821	825	9	1	3.4	59.556	25.495	0.428	43.5	79.76	36.78	10.17
1615	83	5	831	835	9	1	3.6	41.25	15.365	0.372	42.85	77.62	33.91	10.27
1615	84	5	841	845	9	1	3.4	39.669	17.354	0.437	43.18	79.65	35.9	10.19
1615	85	5	851	855	9	1	3.5	49.704	20.94	0.421	43.18	79.25	36.85	10.06
1615	86	5	861	865	9	1	3.8	32.653	11.904	0.365	44.2	79.34	36.86	10
1615	87	5	871	875	9	1	3.8	44.247	15.06	0.34	40.55	76.03	41.73	9.34
1615	88	5	881	885	9	1	3.5	33.163	10.701	0.323	41.91	77.13	37.19	9.32
1615	89	5	891	895	9	1	3.3	33.045	12.657	0.383	41.39	78.72	40.7	9.06
1615	90	5	901	905	9	1	3	57.857	20.135	0.348	41.41	77.95	38.27	9.5
1615	91	5	911	915	10	1	3.3	29.778	10.299	0.346	44.89	81.29	35.19	10.82
1615	92	5	921	925	10	1	3.8	50	16.158	0.323	42.07	75.04	40.48	9.52
1615	93	5	931	935	10	1	3.1	56.25	20.802	0.37	42.55	75.86	41.79	8.75

Table S2 Continued.

Cut	Gen	Cut.Rep	Plot	Int	Block	Rep	Height	GB	DB	DM	ADF	NDF	DIG	LIG
1615	94	5	941	945	10	1	2.8	54.238	19.306	0.356	39.34	72.97	43.89	8.03
1615	95	5	951	955	10	1	3.6	80	30.435	0.38	45.82	80.1	33.72	11.57
1615	96	5	961	965	10	1	3.6	56.213	19.502	0.347	44.64	79.57	41.34	9.12
1615	97	5	971	975	10	1	3.8	78.667	28.309	0.36	42.86	76.96	40.12	9.81
1615	98	5	981	985	10	1	3.2	41	16.557	0.404	45.5	79.95	38.07	10.43
1615	99	5	991	995	10	1	0	0	0	0	0	0	0	0
1615	100	5	1001	1005	10	1	0	0	0	0	0	0	0	0
250	1	6	12	11	11	2	2.95	36.33	17.18	0.45	50.96	80.07	36.42	9.16
250	2	6	22	21	12	2	3.65	58.33	21.87	0.38	50.73	77.99	35.79	9.19
250	3	6	32	31	13	2	3.05	34.67	13.49	0.39	54.16	81.07	32.87	10.31
250	4	6	42	41	14	2	2.5	21.69	7.32	0.34	47.05	75.4	37.56	8.89
250	5	6	52	51	15	2	2.95	29	12.33	0.43	49.76	77.86	37.66	8.35
250	6	6	62	61	16	2	3.65	16.97	7.99	0.47	58.47	84.88	29.68	10.95
250	7	6	72	71	17	2	2.6	25.33	11.29	0.45	54.39	81.33	31.78	11.08
250	8	6	82	81	18	2	2.65	16.83	7.33	0.44	51.73	79.44	35.8	8.99
250	9	6	92	91	19	2	3.1	27.37	11.37	0.42	50.26	78.02	38.29	8.32
250	10	6	102	101	20	2	2.65	25.08	9.69	0.39	54.24	80.49	33.49	10.31
250	11	6	112	111	11	2	2.75	18.67	9.32	0.5	47.05	76.32	39.52	8.1
250	12	6	122	121	12	2	2.2	22.92	10.86	0.47	49.34	77.06	38.42	8.07
250	13	6	132	131	13	2	2.75	17.3	8.12	0.47	52.44	79.83	35.21	9.67
250	14	6	142	141	14	2	2.7	20.56	9.89	0.48	48.89	78.22	37.18	9.05
250	15	6	152	151	15	2	2.85	30.83	11.96	0.39	51.44	77.32	35.11	10.02
250	16	6	162	161	16	2	2.65	15.17	4.65	0.31	45.63	72.69	40.27	8.18
250	17	6	172	171	17	2	2.75	20.5	6.5	0.32	51.01	77.54	34.68	9.2
250	18	6	182	181	18	2	3.3	37	13.4	0.36	52.62	78.74	36.55	9.45
250	19	6	192	191	19	2	2.55	30	14.57	0.49	48.07	76.89	36.02	8.89
250	20	6	202	201	20	2	2.25	19.83	9.4	0.47	50.96	79.87	34.77	8.88
250	21	6	212	211	11	2	2.85	32.9	17.04	0.52	51.6	79.45	34.69	10.07
250	22	6	222	221	12	2	2.25	15.07	6.78	0.45	50.67	77.27	35.41	10.05
250	23	6	232	231	13	2	2.65	29.17	8.26	0.28	51.08	80.08	35.73	9.71
250	24	6	242	241	14	2	2.35	25.95	9.91	0.38	53.51	81.09	32.12	10.9

Table S2 Continued.

Cut	Gen	Cut.Rep	Plot	Int	Block	Rep	Height	GB	DB	DM	ADF	NDF	DIG	LIG
250	25	6	252	251	15	2	2.5	25.33	11.67	0.46	48.87	76.5	37.16	8.51
250	26	6	262	261	16	2	2.3	27.03	12.15	0.45	51.14	80.86	35.81	8.66
250	27	6	272	271	17	2	2.35	27.83	11.64	0.42	51.5	79.46	34.47	9.51
250	28	6	282	281	18	2	2.65	28.83	11.5	0.4	52.53	79.04	35.65	9.51
250	29	6	292	291	19	2	2.4	19	8.37	0.44	53.13	79.37	36.35	9.55
250	30	6	302	301	20	2	2.15	19.5	7.97	0.41	49.24	78.93	38.91	9.46
250	31	6	312	311	11	2	2.05	38.74	15.75	0.41	48.24	77.2	34.9	9.22
250	32	6	322	321	12	2	2.55	32.63	16.33	0.5	50.01	78.96	34.94	9.57
250	33	6	332	331	13	2	2.8	21.17	8.37	0.4	51.47	81.06	33.49	9.83
250	34	6	342	341	14	2	2.25	20.28	9.46	0.47	50.78	79	34.18	10.36
250	35	6	352	351	15	2	2.65	27.03	8.82	0.33	51.27	80.06	32.95	10.14
250	36	6	362	361	16	2	3.6	48.15	21.9	0.46	48.01	76.07	40.63	7.73
250	37	6	372	371	17	2	3.7	48.17	20.13	0.42	51.36	79.09	34.54	10.44
250	38	6	382	381	18	2	2.15	14.67	6.13	0.42	49.41	77.97	35.9	9.5
250	39	6	392	391	19	2	3.55	52.17	21.99	0.42	54.36	81.77	32.25	10.71
250	40	6	402	401	20	2	3.2	38.24	17.68	0.46	50.23	79.51	35.96	9.05
250	41	6	412	411	11	2	2.65	27.33	13.65	0.5	53.01	83.4	35.24	9.77
250	42	6	422	421	12	2	2.35	54.95	22.01	0.4	51.01	80.03	36.19	9.09
250	43	6	432	431	13	2	3.05	31.17	14.59	0.47	52.97	80.35	33.58	9.89
250	44	6	442	441	14	2	2.65	30.93	16.64	0.54	49.37	79.45	34.57	9.32
250	45	6	452	451	15	2	2.85	23.83	9.67	0.41	48.76	76.81	35.47	8.55
250	46	6	462	461	16	2	2.7	10	4.44	0.44	49.51	77.23	36.95	9.18
250	47	6	472	471	17	2	2.4	21.83	10.88	0.5	51.24	80.04	36.23	9.46
250	48	6	482	481	18	2	3.6	41.67	15.67	0.38	47.42	75.73	37.76	8.88
250	49	6	492	491	19	2	1.65	17.17	7.21	0.42	47.83	75.7	37.17	9.14
250	50	6	502	501	20	2	3.2	31.36	14.82	0.47	52.23	81.22	30.38	10.44
250	51	6	512	511	11	2	3.05	45.79	17.32	0.38	48.51	78.83	36.8	8.82
250	52	6	522	521	12	2	3.3	36.14	12.33	0.34	52.6	78.73	36.5	10.06
250	53	6	532	531	13	2	3.85	43.08	14.52	0.34	55.04	82.41	33.31	10.8
250	54	6	542	541	14	2	3.8	43.69	18.12	0.42	53.71	79.95	34.53	10.54
250	55	6	552	551	15	2	3.7	69.5	28.33	0.41	50.5	78.96	36.72	8.68

Table S2 Continued.

Cut	Gen	Cut.Rep	Plot	Int	Block	Rep	Height	GB	DB	DM	ADF	NDF	DIG	LIG
250	56	6	562	561	16	2	3.8	33.33	13.78	0.41	50.25	77.98	32.66	9.55
250	57	6	572	571	17	2	2.45	26	12.36	0.48	50.34	78.22	36.42	8.54
250	58	6	582	581	18	2	3.3	30.17	11.77	0.39	46.71	76.27	40.43	6.82
250	59	6	592	591	19	2	3.5	35.68	16.81	0.47	51.82	78.58	34.54	9.5
250	60	6	602	601	20	2	3.35	41.33	13.89	0.34	52.55	79.72	37.46	9.05
250	61	6	612	611	11	2	3.05	32.17	15.88	0.49	47.02	76.73	37.96	8.29
250	62	6	622	621	12	2	2	17.33	9.37	0.54	50.72	78.93	35.23	8.51
250	63	6	632	631	13	2	2.45	22.83	10.81	0.47	49.01	77.01	38.76	7.91
250	64	6	642	641	14	2	2.45	28.5	13.91	0.49	48.43	77.83	37.15	9
250	65	6	652	651	15	2	2.25	19.83	7.46	0.38	47.2	75.91	39.04	7.92
250	66	6	662	661	16	2	2.25	9.17	4.11	0.45	54.52	81.07	32.31	9.49
250	67	6	672	671	17	2	3.15	45.05	19.13	0.43	49.01	77.97	37.85	8.56
250	68	6	682	681	18	2	2.75	15	5.52	0.37	50.04	76.93	33.62	8.89
250	69	6	692	691	19	2	2.6	23.17	9.92	0.43	48.57	77.39	36.96	8.49
250	70	6	702	701	20	2	2.75	30.5	15.99	0.52	47.83	75.63	38.81	8.1
250	71	6	712	711	11	2	2.25	32.43	15.84	0.49	46.64	76.3	40.48	7.77
250	72	6	722	721	12	2	2.25	31.83	15.46	0.49	51.72	80.66	34.81	9.28
250	73	6	732	731	13	2	2.2	35.24	15.53	0.44	49.83	78.98	37.43	9.45
250	74	6	742	741	14	2	2.95	34.5	11.42	0.33	53.28	79.13	31.07	11.4
250	75	6	752	751	15	2	2.35	27.86	12.65	0.45	47.64	76.17	39.45	8.38
250	76	6	762	761	16	2	2.1	28.5	15.09	0.53	49.19	77.74	37.07	8.9
250	77	6	772	771	17	2	2.9	21.33	7.57	0.36	53.2	78.32	34.54	10.69
250	78	6	782	781	18	2	3.75	42.33	15.75	0.37	50.74	76.8	34.71	9.9
250	79	6	792	791	19	2	1.95	24.67	11.33	0.46	50.14	77.53	35.87	8.86
250	80	6	802	801	20	2	2.2	25.67	12.89	0.5	48.08	77.55	38.52	8.41
250	81	6	812	811	11	2	2.05	24.17	11.34	0.47	49.94	76.97	37.56	8.28
250	82	6	822	821	12	2	2.35	22.42	10	0.45	49.5	78.72	37.79	9.04
250	83	6	832	831	13	2	2.8	24	7.84	0.33	47.67	74.41	38.37	8.71
250	84	6	842	841	14	2	2.4	31.67	15.48	0.49	54.33	81.89	32.32	10.86
250	85	6	852	851	15	2	2.45	18.43	7.64	0.41	52	80.67	35.55	10.3
250	86	6	862	861	16	2	2.2	21.33	9.69	0.45	50.49	79.13	36.03	9.13

Table S2 Continued.

Cut	Gen	Cut.Rep	Plot	Int	Block	Rep	Height	GB	DB	DM	ADF	NDF	DIG	LIG
250	87	6	872	871	17	2	3.85	28.33	9.15	0.32	50.5	79.35	35.71	9.26
250	88	6	882	881	18	2	3.6	37.17	12.98	0.35	53.51	80.55	34.27	10.8
250	89	6	892	891	19	2	2.4	23.83	10.15	0.43	50.14	78.54	37.68	8.92
250	90	6	902	901	20	2	1.9	16.75	8.31	0.5	44.35	74.02	41.02	6.97
250	91	6	912	911	11	2	2.25	14.1	4.16	0.3	46.8	75.12	37.62	8.46
250	92	6	922	921	12	2	3.65	28.11	12.07	0.43	48.66	77.05	35.22	10.21
250	93	6	932	931	13	2	3.5	31.11	10.86	0.35	52.19	79.53	35.85	9.93
250	94	6	942	941	14	2	3.65	29.79	11.28	0.38	52.06	77.68	34.2	9.82
250	95	6	952	951	15	2	4.05	67.25	34.36	0.51	53.54	80.52	34.01	10.61
250	96	6	962	961	16	2	3.85	40.67	16.01	0.39	54.1	81.35	36.24	9.66
250	97	6	972	971	17	2	3.5	41.67	17.43	0.42	47.2	74.85	37.34	8.48
250	98	6	982	981	18	2	3.6	33.51	15.01	0.45	54.34	80.59	31.28	10.73
250	99	6	992	991	19	2	2.4	27	14.22	0.53	51.15	79.27	32.88	10.48
250	100	6	1002	1001	20	2	2.35	21.33	9.84	0.46	45.2	75.52	39.86	7.71
500	1	7	12	12	11	2	4.77	91.06	31.05	0.34	51.16	79.42	32.14	9.88
500	2	7	22	22	12	2	5.07	125.5	52.93	0.42	55.57	81.38	30.9	12.02
500	3	7	32	32	13	2	4.7	87.93	32.79	0.37	53.26	80.86	28.47	11.72
500	4	7	42	42	14	2	4.17	59.58	27.09	0.46	56.94	85.15	33.45	10.94
500	5	7	52	52	15	2	4.27	65.5	25.56	0.39	56.42	82.15	29.63	11.21
500	6	7	62	62	16	2	5	41.85	16.46	0.39	55.37	83.05	30.69	11.4
500	7	7	72	72	17	2	3.93	62.7	27.94	0.45	54.18	81.96	25.28	11.59
500	8	7	82	82	18	2	4	44.5	18.36	0.41	55.96	82.02	31.93	11.18
500	9	7	92	92	19	2	4.1	59.67	25.49	0.43	54.29	82.18	32.61	10.62
500	10	7	102	102	20	2	3.13	78.48	23.04	0.29	54.88	79.93	34.26	11.09
500	11	7	112	112	11	2	4.7	64.84	26.14	0.4	52.13	80.25	33.87	10.08
500	12	7	122	122	12	2	4.4	54.59	23.14	0.42	57.59	84.55	31.6	12.39
500	13	7	132	132	13	2	4.97	77.41	28.45	0.37	51.86	79.28	33.81	10.2
500	14	7	142	142	14	2	4.7	72.53	25.18	0.35	55.59	83.18	32.48	12.44
500	15	7	152	152	15	2	3.67	58.02	20.79	0.36	56.51	82.01	30.13	12.13
500	16	7	162	162	16	2	4.07	79.33	26.4	0.33	52.14	78.41	32.59	11.54
500	17	7	172	172	17	2	4.03	42.74	17.16	0.4	56.36	83.52	24.06	13.2

Table S2 Continued.

Cut	Gen	Cut.Rep	Plot	Int	Block	Rep	Height	GB	DB	DM	ADF	NDF	DIG	LIG
500	18	7	182	182	18	2	4.2	97.5	31.69	0.33	53.54	79.88	32.8	11
500	19	7	192	192	19	2	4.27	72.83	26.24	0.36	57.41	83.37	30.07	12.16
500	20	7	202	202	20	2	4.23	107.5	39.05	0.36	55.15	81.42	32.32	11.88
500	21	7	212	212	11	2	5.07	92.07	41.16	0.45	53.51	81.47	31.88	10.49
500	22	7	222	222	12	2	3.47	38.69	21.87	0.57	60.41	86.86	24.9	14.86
500	23	7	232	232	13	2	4.1	59.44	20.36	0.34	56.04	81.8	24.38	13.12
500	24	7	242	242	14	2	3.87	68.33	23.86	0.35	53.97	81.93	32.91	11.77
500	25	7	252	252	15	2	3.9	53.88	23.3	0.43	55.76	81.79	24.88	13.37
500	26	7	262	262	16	2	4.07	120.56	46.44	0.39	57.4	83.93	29.44	12
500	27	7	272	272	17	2	4.03	58.17	27.72	0.48	54.66	82.06	24.83	11.63
500	28	7	282	282	18	2	3.8	45.17	18	0.4	56.16	83.74	28.24	13.16
500	29	7	292	292	19	2	4.17	54.77	24.02	0.44	56.86	83.09	26.38	12.6
500	30	7	302	302	20	2	3.1	57.12	22.92	0.4	54.92	82.46	31.47	11.63
500	31	7	312	312	11	2	4.07	49.26	20.35	0.41	55.2	81.87	25.7	12.31
500	32	7	322	322	12	2	4.73	98.38	39.89	0.41	53.24	78.81	29.65	10.39
500	33	7	332	332	13	2	4.37	55.71	23.77	0.43	54.57	81.96	32.42	10.26
500	34	7	342	342	14	2	3.77	38.45	17.07	0.44	55.05	82.11	27.19	11.69
500	35	7	352	352	15	2	4.37	55.56	20.62	0.37	54.02	80.96	28.82	11.5
500	36	7	362	362	16	2	5.07	104	28.66	0.28	53.15	79.25	32.78	10.42
500	37	7	372	372	17	2	4.07	103.06	39.3	0.38	51.41	78.71	34.84	8.41
500	38	7	382	382	18	2	3.9	40.33	16.63	0.41	53.28	77.69	29.24	10.29
500	39	7	392	392	19	2	4.1	86.17	37.56	0.44	54.35	80.17	29.74	10.77
500	40	7	402	402	20	2	4.6	90.97	34.53	0.38	49.19	73.84	37.13	8.44
500	41	7	412	412	11	2	4.17	94.41	41.65	0.44	56.03	80.88	29.24	12.52
500	42	7	422	422	12	2	4.73	94.01	42.31	0.45	52.05	77.98	31.87	9.45
500	43	7	432	432	13	2	4.13	73.82	32.31	0.44	54.31	80.16	28.2	11.76
500	44	7	442	442	14	2	4.27	82.04	37.06	0.45	54.27	80.29	30.95	10.37
500	45	7	452	452	15	2	4.07	44.72	15.01	0.34	54.78	79.01	34.29	10.29
500	46	7	462	462	16	2	3.93	28.6	11.46	0.4	53.32	78.58	30.84	10.03
500	47	7	472	472	17	2	4.17	70.99	30.32	0.43	53.92	78.79	33.8	10.68
500	48	7	482	482	18	2	4.43	98	34.52	0.35	53.75	80.22	28.81	11.35

Table S2 Continued.

Cut	Gen	Cut.Rep	Plot	Int	Block	Rep	Height	GB	DB	DM	ADF	NDF	DIG	LIG
500	49	7	492	492	19	2	1.6	27.67	8.54	0.31	50.05	76.36	35.18	9.5
500	50	7	502	502	20	2	4.4	72.69	31.94	0.44	55.64	82.08	25.71	12.05
500	51	7	512	512	11	2	4.47	128.7	51.28	0.4	55.3	80.08	33.54	10.54
500	52	7	522	522	12	2	4.5	94.67	36.95	0.39	50.19	75.5	31.98	9.29
500	53	7	532	532	13	2	3.77	84.13	33.79	0.4	54.59	79.63	29.93	11.42
500	54	7	542	542	14	2	4.07	103.33	44.58	0.43	52.72	79.84	31.39	10.2
500	55	7	552	552	15	2	4.37	114.59	40.24	0.35	56.84	81.32	29.19	11.82
500	56	7	562	562	16	2	4.67	77.48	30.63	0.4	52.27	80.33	28.18	11.12
500	57	7	572	572	17	2	4.03	81	36.39	0.45	53.49	79.48	31.84	10.82
500	58	7	582	582	18	2	3.8	67.39	24.97	0.37	47.89	74.61	37.23	7.73
500	59	7	592	592	19	2	3.9	72.25	32.44	0.45	50.34	76.41	35.51	9.7
500	60	7	602	602	20	2	3.77	68.82	29.19	0.42	53.71	79.46	31.36	10.37
500	61	7	612	612	11	2	5.5	110	42.8	0.39	59.1	83.99	31.66	12.16
500	62	7	622	622	12	2	4.37	41.08	19.76	0.48	54.76	79.99	29.31	9.87
500	63	7	632	632	13	2	4.4	57.59	24.87	0.43	49.16	74.79	36.73	8.58
500	64	7	642	642	14	2	4.93	115.32	41.63	0.36	52.85	79.54	32.86	10.62
500	65	7	652	652	15	2	3.73	46.29	19.05	0.41	52.56	77.28	32.49	11
500	66	7	662	662	16	2	3.87	37.12	16.49	0.44	53.61	78.99	32.34	9.91
500	67	7	672	672	17	2	3.77	78.96	32.87	0.42	54.69	79.53	32.35	11.26
500	68	7	682	682	18	2	4.83	51.92	18.11	0.35	51.12	77.66	32.91	10.38
500	69	7	692	692	19	2	4.6	69.37	27.83	0.4	56.81	81.31	30.51	11.68
500	70	7	702	702	20	2	4.5	117.48	42.19	0.36	56.96	79.68	31.5	11.73
500	71	7	712	712	11	2	3.9	91.34	40.52	0.44	53.66	77.82	32.12	11.6
500	72	7	722	722	12	2	4.43	81.1	36.27	0.45	57.33	82.96	27.47	11.94
500	73	7	732	732	13	2	4.73	61.97	28.33	0.46	54.29	79.3	31.43	11.49
500	74	7	742	742	14	2	4.17	65.95	23.28	0.35	59.47	81.75	20.16	15.39
500	75	7	752	752	15	2	4.3	68.36	30.22	0.44	56.45	82.52	29.63	12.81
500	76	7	762	762	16	2	4.33	78.83	33.02	0.42	50.27	75.54	32.91	8.87
500	77	7	772	772	17	2	3.67	44.5	17.04	0.38	58.13	83.86	26.38	14.54
500	78	7	782	782	18	2	4.77	115.14	43.64	0.38	56.68	81.09	28.23	12.8
500	79	7	792	792	19	2	3.5	75.32	31.91	0.42	52.07	79.17	33.05	10.32

Table S2 Continued.

Cut	Gen	Cut.Rep	Plot	Int	Block	Rep	Height	GB	DB	DM	ADF	NDF	DIG	LIG
500	80	7	802	802	20	2	4.67	104	41.45	0.4	54	79.95	32.62	10.98
500	81	7	812	812	11	2	4.77	97	44.79	0.46	58.79	84.42	29.43	12.66
500	82	7	822	822	12	2	4.4	52.61	24.26	0.46	57.42	82.38	28.9	11.92
500	83	7	832	832	13	2	3.83	67.83	25.53	0.38	57.5	83.25	29.69	12.01
500	84	7	842	842	14	2	4.03	85.23	37.89	0.45	55.44	81.8	27.58	12.35
500	85	7	852	852	15	2	4	45.6	20.24	0.44	60.11	85.87	25.17	13.74
500	86	7	862	862	16	2	3.67	73	32.39	0.44	53.79	81.02	33.26	10.92
500	87	7	872	872	17	2	4.17	63.06	22.14	0.35	58.97	82.86	24.7	13.87
500	88	7	882	882	18	2	4.53	101.14	37.16	0.37	52.29	78.62	31.56	11.31
500	89	7	892	892	19	2	3.77	73	28.99	0.4	57.49	82.39	29.94	12.82
500	90	7	902	902	20	2	3.6	63.74	22.1	0.35	52.45	79.41	33.95	11.55
500	91	7	912	912	11	2	4.27	20.9	8.28	0.4	51.97	79.01	28.25	11.86
500	92	7	922	922	12	2	4.73	77.51	29.71	0.38	48.36	76.88	32.93	10.28
500	93	7	932	932	13	2	4.43	95.35	36.75	0.39	53.85	77.95	31	12.46
500	94	7	942	942	14	2	4.43	50.98	18.82	0.37	49.97	76.45	34.09	10.22
500	95	7	952	952	15	2	4.8	152.97	61.41	0.4	52.2	77.44	30.96	10.77
500	96	7	962	962	16	2	4.77	77.9	29.89	0.38	53.8	78.63	33.73	10.23
500	97	7	972	972	17	2	4.5	94.93	35.74	0.38	52.35	75.85	29.38	10.77
500	98	7	982	982	18	2	4.6	59.46	23.78	0.4	52.86	79.86	33.71	11.02
500	99	7	992	992	19	2	3.23	48.11	19.14	0.4	51.56	77.81	34.21	10.31
500	100	7	1002	1002	20	2	4.5	52.61	15.75	0.3	50.06	76.75	35.29	9.25
815	1	8	12	13	11	2	3.57	67.38	26.95	0.4	52.76	80.63	43.8	8.4
815	2	8	22	23	12	2	3.57	92.47	35.14	0.38	49.25	76.68	35.74	8.92
815	3	8	32	33	13	2	3.5	56.67	18.66	0.33	46.41	75.21	36.51	8.23
815	4	8	42	43	14	2	3.6	59.31	21.98	0.37	51.81	80.91	35.26	9.26
815	5	8	52	53	15	2	3.37	51.24	18.75	0.37	53.53	81.61	33.57	9.4
815	6	8	62	63	16	2	3.87	39.51	14.05	0.36	52.94	82.1	31.44	8.68
815	7	8	72	73	17	2	3.5	54.29	20.42	0.38	52.44	79.15	30.19	10.13
815	8	8	82	83	18	2	3.43	36.04	13.24	0.37	51.52	79.17	36.86	9.19
815	9	8	92	93	19	2	3.47	52.57	21.03	0.4	51.47	79.6	37.21	9.03
815	10	8	102	103	20	2	3.63	70.97	20.01	0.28	49.9	76.06	36	8.48

Table S2 Continued.

Cut	Gen	Cut.Rep	Plot	Int	Block	Rep	Height	GB	DB	DM	ADF	NDF	DIG	LIG
815	11	8	112	113	11	2	3.7	50.86	17.76	0.35	49.77	77.77	34.39	8.34
815	12	8	122	123	12	2	3.57	41.33	15.38	0.37	49.39	77.34	37.02	8.35
815	13	8	132	133	13	2	3.37	45.21	14.63	0.32	53.1	81.68	35.51	9.37
815	14	8	142	143	14	2	3.43	46.44	14.79	0.32	48.97	76.83	38.83	7.95
815	15	8	152	153	15	2	3.43	32.76	11.19	0.34	51.85	79.17	30.68	10.15
815	16	8	162	163	16	2	3.63	59.43	18.76	0.32	53.15	79.42	29.56	10.7
815	17	8	172	173	17	2	3.6	29.79	10.13	0.34	52.49	80.66	31.89	9.03
815	18	8	182	183	18	2	3.4	70.86	21.08	0.3	49.29	77.39	37.22	8.25
815	19	8	192	193	19	2	3.47	47.62	15.8	0.33	49.1	76.9	39.15	8.03
815	20	8	202	203	20	2	3.57	67.62	18.88	0.28	51.66	79.57	39.32	8.71
815	21	8	212	213	11	2	3.6	85.9	27.71	0.32	51.39	79.44	34.99	8.9
815	22	8	222	223	12	2	3.1	42.42	16.91	0.4	55.83	82.54	25.5	11.36
815	23	8	232	233	13	2	3.57	43.22	13.46	0.31	52.88	80.47	28.9	10.86
815	24	8	242	243	14	2	3.2	49.33	14.59	0.3	49.57	78.64	32.71	8.44
815	25	8	252	253	15	2	3.03	48.13	18.42	0.38	54.64	80.93	27.81	10.99
815	26	8	262	263	16	2	3.03	41.6	13.93	0.33	50.42	78.22	33.48	8.96
815	27	8	272	273	17	2	3.6	40.19	17.33	0.43	49.88	77.48	32.38	7.96
815	28	8	282	283	18	2	3.23	40.38	14.91	0.37	50.89	77.87	35.88	9.21
815	29	8	292	293	19	2	3.7	79.18	29.7	0.38	53.09	79.81	33.06	9.5
815	30	8	302	303	20	2	2.93	32	10.56	0.33	53.56	81.75	31.57	9.71
815	31	8	312	313	11	2	3.67	31.67	11.85	0.37	52.05	79.27	32.43	9.97
815	32	8	322	323	12	2	3.73	62.29	23.55	0.38	50.71	78.02	24.69	9.66
815	33	8	332	333	13	2	3.43	56.77	20.13	0.35	49.96	78.29	30.91	9.57
815	34	8	342	343	14	2	2.97	28.85	10.09	0.35	50.15	77.73	33.48	8.86
815	35	8	352	353	15	2	3.7	29.58	10.27	0.35	50.3	77.73	34.11	10.02
815	36	8	362	363	16	2	3.9	88.95	23.46	0.26	51.09	78.94	37.14	9.43
815	37	8	372	373	17	2	3.53	65	21.44	0.33	52.35	81	32.48	9.37
815	38	8	382	383	18	2	3.17	36.25	12.89	0.36	54.25	82.25	31.11	10.15
815	39	8	392	393	19	2	3.47	57.5	20.18	0.35	50.91	80.85	37.32	9.05
815	40	8	402	403	20	2	3.67	72.82	20.56	0.28	47.27	78.16	41.8	7.87
815	41	8	412	413	11	2	3.73	84	31.69	0.38	49.41	78.59	33.39	8.98

Table S2 Continued.

Cut	Gen	Cut.Rep	Plot	Int	Block	Rep	Height	GB	DB	DM	ADF	NDF	DIG	LIG
815	42	8	422	423	12	2	3.5	57.14	20.8	0.36	47.57	75.64	36.1	7.54
815	43	8	432	433	13	2	3.2	39.62	16.3	0.41	51.86	81.85	29.88	9.5
815	44	8	442	443	14	2	3.37	76.88	31.81	0.41	50.84	79.54	28.72	9.14
815	45	8	452	453	15	2	3.6	45.13	13.49	0.3	48.52	75.66	36.03	8.56
815	46	8	462	463	16	2	3.33	20.65	6.68	0.32	45.7	74.45	34.24	8.24
815	47	8	472	473	17	2	3.77	93.75	34.18	0.36	52.47	80.76	31.51	9.36
815	48	8	482	483	18	2	3.97	76.19	23.08	0.3	49.32	78.07	37.27	9.23
815	49	8	492	493	19	2	2.1	16.32	4.41	0.27	44.75	74.54	47.24	6.54
815	50	8	502	503	20	2	3.7	54.81	19.85	0.36	53.78	81.13	31.87	9.98
815	51	8	512	513	11	2	3.57	72.12	24.73	0.34	47.09	76.41	43.1	7.62
815	52	8	522	523	12	2	3.57	72.95	24.77	0.34	48.99	78.89	38.17	8.7
815	53	8	532	533	13	2	3.53	90.46	33.65	0.37	48.82	78.12	35.32	8.91
815	54	8	542	543	14	2	3.73	91.58	31.21	0.34	50.55	78.47	32.63	8.3
815	55	8	552	553	15	2	3.73	97.14	36.07	0.37	50.84	78.59	27.34	9.25
815	56	8	562	563	16	2	3.8	46.06	13.51	0.29	52.32	80.74	42.16	9.8
815	57	8	572	573	17	2	3.57	54.34	19.91	0.37	51.52	79.72	32.6	9.52
815	58	8	582	583	18	2	3.4	64.9	18.94	0.29	47.73	78.24	39.04	7.24
815	59	8	592	593	19	2	3.5	60.53	23.46	0.39	51.37	80.51	32.95	9.1
815	60	8	602	603	20	2	3.57	47.36	15.86	0.33	49.6	80.07	34.22	9.63
815	61	8	612	613	11	2	3.13	39.56	12.51	0.32	45.6	74.16	46.6	6.59
815	62	8	622	623	12	2	2.77	27.3	10.35	0.38	52.03	78.64	30	8.88
815	63	8	632	633	13	2	3.57	51.2	18.02	0.35	49.71	77.92	39.72	7.99
815	64	8	642	643	14	2	3.43	56.38	17.77	0.32	50.74	79.04	30.38	9.01
815	65	8	652	653	15	2	3.1	33.13	11.78	0.36	50.54	78.74	26.78	10.08
815	66	8	662	663	16	2	3.4	29.88	10.72	0.36	52.12	80.64	31.96	8.98
815	67	8	672	673	17	2	3.5	48.33	19.72	0.41	49.62	77.83	33.72	9.97
815	68	8	682	683	18	2	3.7	44.15	12.65	0.29	54.1	80.67	30.15	9.63
815	69	8	692	693	19	2	3.6	66.67	24.75	0.37	53.51	80.64	32.56	10.25
815	70	8	702	703	20	2	3.77	88.57	25.65	0.29	50.79	81.06	42.58	8
815	71	8	712	713	11	2	2.87	42.63	15.28	0.36	50.31	77.28	30.3	8.95
815	72	8	722	723	12	2	3.43	75	28.5	0.38	NA	NA	NA	NA

Table S2 Continued.

Cut	Gen	Cut.Rep	Plot	Int	Block	Rep	Height	GB	DB	DM	ADF	NDF	DIG	LIG
815	73	8	732	733	13	2	3.5	71.18	25.63	0.36	53.28	81.47	33.56	9.83
815	74	8	742	743	14	2	3.73	39.6	12.6	0.32	53.27	78.86	25.83	11.2
815	75	8	752	753	15	2	3.47	55.24	21.47	0.39	53.12	82.03	28.75	10.11
815	76	8	762	763	16	2	3.53	58.1	23.4	0.4	46.29	74.42	30.28	7.93
815	77	8	772	773	17	2	3	49.79	18.6	0.37	51.55	79.74	37.71	9.87
815	78	8	782	783	18	2	3.9	88.57	24.35	0.27	52.04	81.25	30.7	9.61
815	79	8	792	793	19	2	3.43	69.9	25.3	0.36	50.97	80.84	37.34	8.7
815	80	8	802	803	20	2	3.53	63.66	22.4	0.35	51.68	80.11	37.65	9.78
815	81	8	812	813	11	2	3.13	54.48	20.55	0.38	49.18	77.51	34.6	8.89
815	82	8	822	823	12	2	3.5	52.38	20.03	0.38	48.41	77.26	35.01	8.22
815	83	8	832	833	13	2	3.6	85.14	26.26	0.31	49.8	77.65	37.59	9.41
815	84	8	842	843	14	2	3.37	64.76	26.58	0.41	52.55	80.58	32.47	9.41
815	85	8	852	853	15	2	3.53	35.8	14.13	0.39	51.96	80.34	25.44	9.38
815	86	8	862	863	16	2	3.23	45.11	17.43	0.39	49.37	77.83	36.08	8.04
815	87	8	872	873	17	2	3.67	28.19	9.35	0.33	50.97	77.13	33.02	10.11
815	88	8	882	883	18	2	3.87	80.38	25.99	0.32	52.78	80.7	31.31	10.21
815	89	8	892	893	19	2	3.5	77.33	30.33	0.39	51.01	79.2	34.22	9.34
815	90	8	902	903	20	2	3.5	53.71	19.01	0.35	49.75	78.31	38.7	8.12
815	91	8	912	913	11	2	1.7	32.35	10.64	0.33	48.22	74.64	35.66	9.38
815	92	8	922	923	12	2	3.63	76.19	21.66	0.28	48.07	76.77	36.56	8.67
815	93	8	932	933	13	2	4.17	93.07	31.33	0.34	50.29	77.55	38.88	9.49
815	94	8	942	943	14	2	3.87	42.08	15.25	0.36	49.77	77.49	37.85	9.13
815	95	8	952	953	15	2	4.13	96.76	26.97	0.28	51.48	79.77	33.47	8.72
815	96	8	962	963	16	2	3.67	61.04	20.94	0.34	51.31	79.42	36.9	9.16
815	97	8	972	973	17	2	3.8	57.52	21.47	0.37	51.04	80.96	32.07	9.22
815	98	8	982	983	18	2	3.63	57.87	21.47	0.37	52.85	81.78	30.44	10.16
815	99	8	992	993	19	2	2.57	20.31	7	0.34	49.99	78.07	39.6	8.41
815	100	8	1002	1003	20	2	2.63	20.65	5.84	0.28	0	73.42	45.27	5.75
1405	1	9	12	14	11	2	2.2	49.33	18.09	0.37	48.77	80.03	38.76	8.77
1405	2	9	22	24	12	2	3	92.22	32.06	0.35	51.86	80.81	36.58	10.19
1405	3	9	32	34	13	2	2.5	66.67	24.88	0.37	48.2	79.05	39.04	9.25

Table S2 Continued.

Cut	Gen	Cut.Rep	Plot	Int	Block	Rep	Height	GB	DB	DM	ADF	NDF	DIG	LIG
1405	4	9	42	44	14	2	2.4	53.33	13.6	0.26	NA	NA	NA	NA
1405	5	9	52	54	15	2	2.4	36.44	15.78	0.43	47.17	79.3	41.49	8.32
1405	6	9	62	64	16	2	2.4	13.78	3.54	0.26	48.39	78.89	40.32	7.9
1405	7	9	72	74	17	2	2.5	50.67	22.15	0.44	47.95	73.83	40.2	9.25
1405	8	9	82	84	18	2	2.2	19.56	7.43	0.38	45.05	74.41	42.67	7.13
1405	9	9	92	94	19	2	2.1	32.44	14.93	0.46	46.47	75.57	40.29	7.46
1405	10	9	102	104	20	2	3.2	57.78	23.92	0.41	42.5	73.43	42.29	6.14
1405	11	9	112	114	11	2	2.5	74	27.26	0.37	50.69	79.4	38.34	9.04
1405	12	9	122	124	12	2	2.7	38.22	14.84	0.39	49.63	79.75	38.37	9.44
1405	13	9	132	134	13	2	2.3	38.22	16.02	0.42	55.05	84.6	32.31	11.08
1405	14	9	142	144	14	2	1.6	31.56	14.21	0.45	46.44	78.94	41.83	7.73
1405	15	9	152	154	15	2	1.6	26	8.02	0.31	48.43	78.33	41.38	8.68
1405	16	9	162	164	16	2	3	66	17.62	0.27	NA	NA	NA	NA
1405	17	9	172	174	17	2	2.4	39.33	13.43	0.34	47.36	75.68	40.49	8.79
1405	18	9	182	184	18	2	2.3	55.56	17.04	0.31	47.7	76.16	43.61	7.2
1405	19	9	192	194	19	2	1.7	48.89	18.8	0.38	46.94	75.76	38.7	7.87
1405	20	9	202	204	20	2	2.3	36.44	15.27	0.42	51.01	77.86	36.75	8.99
1405	21	9	212	214	11	2	1.9	80	32.95	0.41	50.07	79.24	38.3	9.36
1405	22	9	222	224	12	2	2.1	33.78	11.1	0.33	50.63	79.98	37.06	9.51
1405	23	9	232	234	13	2	2.3	33.33	9.21	0.28	53.66	77.07	33.69	10.84
1405	24	9	242	244	14	2	2.1	19.56	5.64	0.29	48.38	76.35	40.11	8.87
1405	25	9	252	254	15	2	2.1	34.67	15.04	0.43	48.92	78.72	37.27	9.67
1405	26	9	262	264	16	2	2.4	94	25.5	0.27	52.1	79.9	38.73	8.49
1405	27	9	272	274	17	2	2.6	59.56	26.23	0.44	48.31	77.54	40.53	7.72
1405	28	9	282	284	18	2	2.1	22.67	8.08	0.36	47.81	76.73	41.14	8.19
1405	29	9	292	294	19	2	2.4	48.89	25.44	0.52	49.34	76.93	40.48	7.71
1405	30	9	302	304	20	2	2.4	22.22	9.34	0.42	46.2	73.87	41.16	7.73
1405	31	9	312	314	11	2	2.3	29.33	8.04	0.27	46.71	75.51	45.07	6.15
1405	32	9	322	324	12	2	2.5	35.11	11.93	0.34	49.07	76.28	37.8	9.29
1405	33	9	332	334	13	2	3	76	27.27	0.36	51.61	81.95	33.17	10.46
1405	34	9	342	344	14	2	2.4	15.11	6.88	0.46	45.99	75.96	40.86	8.14

Table S2 Continued.

Cut	Gen	Cut.Rep	Plot	Int	Block	Rep	Height	GB	DB	DM	ADF	NDF	DIG	LIG
1405	35	9	352	354	15	2	2.3	16.44	5.11	0.31	45.67	75.58	42.6	7.83
1405	36	9	362	364	16	2	3	57.78	17.94	0.31	45.48	74.01	42.65	6.94
1405	37	9	372	374	17	2	2.4	40	17.13	0.43	50.46	79.36	37.31	9.17
1405	38	9	382	384	18	2	2.1	37.56	14.87	0.4	48.93	79.15	42.43	7.39
1405	39	9	392	394	19	2	3	46.67	19.23	0.41	50.08	79.19	38.81	9.51
1405	40	9	402	404	20	2	3	65.56	27.52	0.42	50.79	78.07	39.35	9.39
1405	41	9	412	414	11	2	2.4	45.56	15.59	0.39	48.49	79.47	38.92	9.07
1405	42	9	422	424	12	2	2.5	42.67	14.03	0.33	51.35	81.39	38.3	9.67
1405	43	9	432	434	13	2	2.45	45.78	17.89	0.39	47.2	79.91	41.1	8.1
1405	44	9	442	444	14	2	2.3	65.56	32.02	0.49	42.14	72.19	42.47	6.66
1405	45	9	452	454	15	2	2.5	27.56	7.85	0.29	47.42	77.51	44.17	7.34
1405	46	9	462	464	16	2	2.5	25.78	10.93	0.42	50.41	82.76	37.32	9.08
1405	47	9	472	474	17	2	2.2	66.44	25.55	0.38	49.9	80.64	41.38	8.3
1405	48	9	482	484	18	2	2.9	28.44	7.01	0.25	43.46	72.62	45.44	6.03
1405	49	9	492	494	19	2	0.9	6.22	2.18	0.35	40.23	68.9	50.64	4.74
1405	50	9	502	504	20	2	3	33.33	7.72	0.23	48.65	76.92	46.72	6.26
1405	51	9	512	514	11	2	2.6	60.22	13.19	0.26	53.74	82.39	37	10.4
1405	52	9	522	524	12	2	3	36.44	13.73	0.38	52.11	81.87	35.14	10.7
1405	53	9	532	534	13	2	3.1	60	22.32	0.37	NA	NA	NA	NA
1405	54	9	542	544	14	2	3	37.78	14.45	0.38	48.28	77.78	38.3	8.52
1405	55	9	552	554	15	2	2.8	59.11	21.96	0.37	47.68	76.46	38.7	8.74
1405	56	9	562	564	16	2	2.4	32	11.27	0.35	51.06	78.93	36.09	9.5
1405	57	9	572	574	17	2	2.1	32.67	9.8	0.3	48.2	77.84	41.72	7.11
1405	58	9	582	584	18	2	3.2	60.22	20.13	0.33	47.57	77.6	46.83	6.18
1405	59	9	592	594	19	2	2.8	38.67	15.52	0.4	47.96	78.99	40.38	8.12
1405	60	9	602	604	20	2	1.9	20.44	7.92	0.39	48.22	78.04	39.74	8.86
1405	61	9	612	614	11	2	2.9	64	19.75	0.31	52.04	72.09	39.15	8.57
1405	62	9	622	624	12	2	2	26.22	10.04	0.38	47.5	78.16	40.27	7.73
1405	63	9	632	634	13	2	2.5	52.89	20.38	0.39	50.2	81.89	36.09	10.07
1405	64	9	642	644	14	2	2.1	48.89	18.81	0.38	45.34	73.6	39.3	7.22
1405	65	9	652	654	15	2	NA	26.22	8.99	0.34	48.13	77.49	40.97	8.66

Table S2 Continued.

Cut	Gen	Cut.Rep	Plot	Int	Block	Rep	Height	GB	DB	DM	ADF	NDF	DIG	LIG
1405	66	9	662	664	16	2	2.1	28	10.96	0.39	45.23	76.87	42.95	7.09
1405	67	9	672	674	17	2	2.7	44	16.73	0.38	49.13	79.36	39.65	8.55
1405	68	9	682	684	18	2	2.6	36	14.33	0.4	49.92	77.97	37.56	8.8
1405	69	9	692	694	19	2	2.6	36.89	18.08	0.49	49.14	77.92	38.59	8.59
1405	70	9	702	704	20	2	2.5	69.33	29.73	0.43	48.14	77.65	40.46	7.95
1405	71	9	712	714	11	2	1.8	33.33	9.63	0.29	52.58	71.19	37.58	9.62
1405	72	9	722	724	12	2	2.2	67.33	25.15	0.37	49.59	79.28	37.4	8.8
1405	73	9	732	734	13	2	2.3	77.33	27.34	0.35	47.03	76.33	43.42	7.74
1405	74	9	742	744	14	2	1.8	16	4.93	0.31	50.35	76.44	35.22	9.42
1405	75	9	752	754	15	2	2.3	58.22	20.63	0.35	48.96	79.21	40.07	8.04
1405	76	9	762	764	16	2	2.3	24.44	7.58	0.31	45.59	78.28	44.37	6.97
1405	77	9	772	774	17	2	2.7	18.67	6.12	0.33	50.59	79.9	37.02	9.57
1405	78	9	782	784	18	2	2.3	40.22	14.53	0.36	50.3	77.48	36.97	9.27
1405	79	9	792	794	19	2	1.8	52	15.48	0.3	46.47	74.52	44.09	6.5
1405	80	9	802	804	20	2	2.7	64.44	18.05	0.28	48.08	78.15	42.82	8.48
1405	81	9	812	814	11	2	2.3	64.67	20.25	0.31	50.39	79.91	36.71	9.45
1405	82	9	822	824	12	2	2.3	61.78	24.52	0.4	47.76	78.29	39.54	8.69
1405	83	9	832	834	13	2	2.4	45.67	15.62	0.34	47.88	77.87	40.53	7.73
1405	84	9	842	844	14	2	1.65	43.11	17.99	0.42	45	76.74	38.57	8.9
1405	85	9	852	854	15	2	2.5	23.56	10.4	0.44	46.97	79.16	39.52	8.2
1405	86	9	862	864	16	2	2.4	16.67	4.56	0.27	46.08	76.94	43.77	6.65
1405	87	9	872	874	17	2	2.8	44.44	14.7	0.33	51.32	79.3	37.98	9.69
1405	88	9	882	884	18	2	2.6	56	18.85	0.34	53.45	80.35	35.8	10.09
1405	89	9	892	894	19	2	1.6	50.44	22.7	0.45	47.35	74.94	40.28	8.5
1405	90	9	902	904	20	2	2.8	36.89	13.5	0.37	43.9	71.73	41.38	5.75
1405	91	9	912	914	11	2	2.2	3.33	1.09	0.33	51.36	79.72	36.53	10.27
1405	92	9	922	924	12	2	1.6	8.44	2.66	0.31	47.06	77.34	40.13	8.28
1405	93	9	932	934	13	2	3.3	59.56	22.14	0.37	50.74	76.08	38.16	9.61
1405	94	9	942	944	14	2	3.4	22.22	7.32	0.33	52.39	79.45	36.45	9.87
1405	95	9	952	954	15	2	3.7	63.33	23.24	0.37	49.24	80.19	39.34	8.37
1405	96	9	962	964	16	2	2.8	48.89	17.78	0.36	51.86	79.38	40.41	8.61

Table S2 Continued.

Cut	Gen	Cut.Rep	Plot	Int	Block	Rep	Height	GB	DB	DM	ADF	NDF	DIG	LIG
1405	97	9	972	974	17	2	2.6	38.44	13.55	0.35	47.18	76.53	39.05	8.14
1405	98	9	982	984	18	2	NA	NA	NA	NA	NA	NA	NA	NA
1405	99	9	992	994	19	2	2.7	6.67	2.01	0.3	NA	NA	NA	NA
1405	100	9	1002	1004	20	2	0.3	5	1.55	0.31	NA	NA	NA	NA
1615	1	10	12	15	11	2	3.4	90.933	30.919	0.34	42.93	77.33	42.17	9.55
1615	2	10	22	25	12	2	3.3	103.491	36.464	0.352	44.33	77.97	39.27	9.28
1615	3	10	32	35	13	2	3.5	60.8	20.523	0.338	43.2	75.88	40.25	8.8
1615	4	10	42	45	14	2	3.1	60.417	22.981	0.38	45.49	79.97	39.27	9.24
1615	5	10	52	55	15	2	3.3	31.111	11.432	0.367	45.63	78.9	37.24	8.85
1615	6	10	62	65	16	2	3.3	25.62	8.386	0.327	49.46	80.48	36.35	8.71
1615	7	10	72	75	17	2	3.2	41.111	15.415	0.375	48.62	80.78	36.26	9.81
1615	8	10	82	85	18	2	3.2	61.454	23.799	0.387	46.24	80.64	37.84	9.03
1615	9	10	92	95	19	2	3.3	34.444	13.237	0.384	44.76	78.88	40.43	9
1615	10	10	102	105	20	2	3.2	41.785	12.096	0.289	42.41	76.93	40.65	8.69
1615	11	10	112	115	11	2	3.2	52	17.408	0.335	44.27	79.46	44.41	8.75
1615	12	10	122	125	12	2	3.4	52.444	19.878	0.379	48.84	80.45	40.51	8.72
1615	13	10	132	135	13	2	3.6	68.217	25.416	0.373	46.86	81.08	39.32	9.29
1615	14	10	142	145	14	2	3.3	46.528	17.089	0.367	41.31	76.6	44.86	8.31
1615	15	10	152	155	15	2	3.2	22.444	6.951	0.31	52.53	80.25	32.96	10.43
1615	16	10	162	165	16	2	3.5	58.667	19.244	0.328	46.1	78.4	37.66	9.36
1615	17	10	172	175	17	2	3	26.337	9.87	0.375	47.73	80.56	36.11	9.83
1615	18	10	182	185	18	2	2.8	57.889	20.707	0.358	46.37	79.34	39.49	9.24
1615	19	10	192	195	19	2	3.8	44.889	14.98	0.334	46.88	79.46	40.54	8.8
1615	20	10	202	205	20	2	3.4	44.444	14.528	0.327	45.67	79.15	39.16	9.3
1615	21	10	212	215	11	2	3.4	57.556	21.365	0.371	44.63	80.08	39.38	9.21
1615	22	10	222	225	12	2	2.8	34.796	14.096	0.405	40.2	75.58	41.07	8.93
1615	23	10	232	235	13	2	2.4	8.681	3.081	0.355	45.06	78.23	34.16	11.74
1615	24	10	242	245	14	2	3.6	28.444	9.7	0.341	42.23	77.06	40.95	9.23
1615	25	10	252	255	15	2	3.5	47.188	15.842	0.336	50.16	81.03	36.61	9.75
1615	26	10	262	265	16	2	3.6	55.556	18.286	0.329	46.41	78.35	37.22	9.07
1615	27	10	272	275	17	2	3.2	31.111	12.636	0.406	47.77	80.46	38.34	9.3

Table S2 Continued.

Cut	Gen	Cut.Rep	Plot	Int	Block	Rep	Height	GB	DB	DM	ADF	NDF	DIG	LIG
1615	28	10	282	285	18	2	2.8	32.889	11.995	0.365	43.27	76.65	43.16	8.81
1615	29	10	292	295	19	2	3.6	66.645	27.341	0.41	48.96	81.19	37.42	10.27
1615	30	10	302	305	20	2	3.3	45.778	15.699	0.343	47.95	78.91	37.26	9.51
1615	31	10	312	315	11	2	3.2	49.905	18.889	0.379	46.03	81.24	35.83	10.86
1615	32	10	322	325	12	2	3.3	36.214	13.331	0.368	42.24	78.25	36.24	9.94
1615	33	10	332	335	13	2	3.3	31.429	12.38	0.394	47.98	80.85	35.01	10.47
1615	34	10	342	345	14	2	3.2	24.494	10.799	0.441	46.87	80.52	34.92	11.23
1615	35	10	352	355	15	2	3.6	17.01	5.878	0.346	44.58	78.89	39.35	9.46
1615	36	10	362	365	16	2	3.8	68.889	20.48	0.297	47.02	79.2	36.78	9.58
1615	37	10	372	375	17	2	3	76.818	29.797	0.388	49.14	81.83	37.74	9.8
1615	38	10	382	385	18	2	3.4	54.931	19.935	0.363	43.52	76.43	38.44	9.77
1615	39	10	392	395	19	2	3.2	76.269	27.6	0.362	41.09	74.66	43.19	8.21
1615	40	10	402	405	20	2	4	98.909	32.266	0.326	43.65	74.16	39.59	7.69
1615	41	10	412	415	11	2	4.5	87.556	32.977	0.377	44.26	77.96	39.25	9.47
1615	42	10	422	425	12	2	3.4	99.395	40.902	0.412	46.35	78.76	38.31	8.77
1615	43	10	432	435	13	2	3.2	68.667	26.841	0.391	41.47	77.7	43.32	7.99
1615	44	10	442	445	14	2	3.5	85.048	38.727	0.455	47.44	80.69	37.73	9.82
1615	45	10	452	455	15	2	3.6	68.027	21.231	0.312	48.52	78.86	36.69	9.73
1615	46	10	462	465	16	2	3.2	38.462	13.329	0.347	47.29	79.71	35.25	10.17
1615	47	10	472	475	17	2	2	77.215	30.886	0.4	NA	NA	NA	NA
1615	48	10	482	485	18	2	4	54.667	17.59	0.322	43.99	75.64	39.81	9.06
1615	49	10	492	495	19	2	1.4	27.778	7.789	0.28	34.32	69.08	48.34	6.99
1615	50	10	502	505	20	2	3.2	89.463	32.648	0.365	48.59	81.19	35.38	10.83
1615	51	10	512	515	11	2	3.5	72.245	24.102	0.334	42.38	78.52	41.71	8.92
1615	52	10	522	525	12	2	3.2	59.556	20.896	0.351	47.05	80.03	32.23	10.98
1615	53	10	532	535	13	2	3.4	62.413	21.628	0.347	43.01	77.9	37.78	9.07
1615	54	10	542	545	14	2	3.5	104.082	38.851	0.373	47.11	80.08	37.14	9.85
1615	55	10	552	555	15	2	3.3	72	24.41	0.339	43.78	77.46	39.42	8.4
1615	56	10	562	565	16	2	3.8	46.429	14.062	0.303	44.75	75.05	40.58	8.23
1615	57	10	572	575	17	2	3.4	33.163	11.313	0.341	47.9	80.96	39.03	9.07
1615	58	10	582	585	18	2	3.2	76.331	23.004	0.301	48.61	77.49	41.58	8.36

Table S2 Continued.

Cut	Gen	Cut.Rep	Plot	Int	Block	Rep	Height	GB	DB	DM	ADF	NDF	DIG	LIG
1615	59	10	592	595	19	2	3	50.694	18.965	0.374	46.64	76.09	41.98	8.13
1615	60	10	602	605	20	2	3.4	31.25	11.167	0.357	43.96	76.52	40.72	8.7
1615	61	10	612	615	11	2	3.7	86.4	28.526	0.33	45.7	79.97	34.95	11.01
1615	62	10	622	625	12	2	2.5	31.328	12.022	0.384	46.43	78.84	38.56	8.7
1615	63	10	632	635	13	2	2.7	23.111	9.355	0.405	39.1	75.06	44.3	8.19
1615	64	10	642	645	14	2	3.4	81.333	NA	NA	NA	NA	NA	NA
1615	65	10	652	655	15	2	3.8	30.612	10.563	0.345	45.85	79.11	34.46	10.5
1615	66	10	662	665	16	2	3.3	75.868	28.375	0.374	46.33	79.75	38.94	8.9
1615	67	10	672	675	17	2	2.9	74.518	27.888	0.374	45.16	78.84	39.45	9.15
1615	68	10	682	685	18	2	3.5	83.884	26.148	0.312	48.99	80.4	32.57	10.9
1615	69	10	692	695	19	2	3.6	33.333	11.196	0.336	50.69	81.85	36.22	9.52
1615	70	10	702	705	20	2	3	30.444	10.619	0.349	50	81.33	36.94	9.75
1615	71	10	712	715	11	2	3.2	33.673	13.324	0.396	43.68	78.49	39.63	10.03
1615	72	10	722	725	12	2	3	62.551	27.244	0.436	45.73	79.63	38.31	9.28
1615	73	10	732	735	13	2	2.5	72.781	23.571	0.324	47.35	80.94	30.84	12.04
1615	74	10	742	745	14	2	3	13.776	5	0.333	NA	NA	NA	NA
1615	75	10	752	755	15	2	3.6	100.567	37.738	0.375	48.06	79.07	37.33	10.41
1615	76	10	762	765	16	2	3	37.778	14.308	0.379	46.57	78.26	38.64	8.61
1615	77	10	772	775	17	2	3.3	19.204	6.342	0.33	46.87	78.98	35.49	9.88
1615	78	10	782	785	18	2	3.8	54.444	19.273	0.354	NA	NA	NA	NA
1615	79	10	792	795	19	2	3	37.778	14.723	0.39	45.83	78.61	39.44	9.24
1615	80	10	802	805	20	2	3.6	24.444	7.685	0.314	49.77	81.12	37.96	9.22
1615	81	10	812	815	11	2	3.4	69.778	24.23	0.347	45.19	79.21	39.88	9.55
1615	82	10	822	825	12	2	3.4	62.222	27.593	0.443	43.61	78.35	39.64	10.06
1615	83	10	832	835	13	2	3.4	59.778	22.499	0.376	41.97	76.98	41.7	8.93
1615	84	10	842	845	14	2	3.6	68.444	25.649	0.375	49.39	81.71	30.77	11.33
1615	85	10	852	855	15	2	3.6	49.587	19.635	0.396	48.47	81.91	35.09	9.99
1615	86	10	862	865	16	2	2.8	32.64	12.859	0.394	49.16	80.33	38.35	9.08
1615	87	10	872	875	17	2	3.5	62.667	20.438	0.326	44.87	78.84	35.09	10.25
1615	88	10	882	885	18	2	3.8	72.667	20.905	0.288	43.46	74.29	37.03	9.63
1615	89	10	892	895	19	2	3.4	50	19.002	0.38	50.41	80.56	37.63	8.98

Table S2 Continued.

Cut	Gen	Cut.Rep	Plot	Int	Block	Rep	Height	GB	DB	DM	ADF	NDF	DIG	LIG
1615	90	10	902	905	20	2	3	34.444	12.823	0.372	47.58	80.51	39.48	8.97
1615	91	10	912	915	11	2	NA	NA	NA	NA	NA	NA	NA	NA
1615	92	10	922	925	12	2	3.4	27.977	9.668	0.346	41.72	76.41	41.54	8.95
1615	93	10	932	935	13	2	3.6	84	30.545	0.364	44.43	76.7	39.27	9.56
1615	94	10	942	945	14	2	3.6	23.717	8.333	0.351	47.99	79.34	36.62	9.36
1615	95	10	952	955	15	2	4.5	64.889	21.501	0.331	45.74	78.82	38.79	9.08
1615	96	10	962	965	16	2	3.6	67.215	22.887	0.34	47.99	80.22	38.32	9.24
1615	97	10	972	975	17	2	3.6	52.222	18.359	0.352	44.81	75	42.29	8.37
1615	98	10	982	985	18	2	3.8	65	22.126	0.34	46.98	78.84	38.03	8.71
1615	99	10	992	995	19	2	0	0	0	0	0	0	0	0
1615	100	10	1002	1005	20	2	0	0	0	0	0	0	0	0
