# Supplementary material for: Unraveling candidate genes underlying biomass digestibility in elephant grass (Cenchrus purpureus)
Source: BMC Plant Biol. 2019 Dec 10;19:548. doi: 10.1186/s12870-019-2180-5 (PMC6905061; doi:10.1186/s12870-019-2180-5)
Supplement: Supplementary file 3 — Additional file 3: Table S3. Molecular data for the 90 elephant grass genotypes. “Code” indicates de code of the genotypes (see Additional file 1: Table S1). [file 12870_2019_2180_MOESM3_ESM.pdf]

Table S3 Molecular data for the 90 elephant grass genotypes. "Code" indicates de code of the accessions (see Table S1).

Code	M41_120	M41_128	M41_130	M12_294	M4_235	M4_243	M4_254	M4_256	M4_258	M4_260	M4_265	M4_266	M4_267	M4_269	M4_271	M4_275	M4_281	M4_285	M4_289
1	1	0	1	1	0	1	0	0	0	0	0	0	0	0	0	0	0	0	0
3	1	0	1	1	0	0	0	0	0	0	0	0	0	1	0	0	0	0	0
4	1	0	1	1	0	0	1	0	0	1	0	1	0	0	0	0	0	0	0
5	1	0	1	1	0	0	0	0	0	0	0	1	0	1	0	0	0	0	0
6	1	0	1	1	0	0	0	1	0	0	0	1	0	0	0	0	0	0	0
7	1	0	1	1	0	0	0	1	0	0	0	0	1	0	0	0	0	0	0
9	1	0	1	1	0	0	1	0	0	1	0	1	0	1	0	0	0	0	0
10	1	0	1	1	0	0	0	0	0	0	1	0	0	1	0	0	0	0	0
11	1	0	1	1	0	1	0	0	0	0	0	0	0	0	0	0	0	0	0
12	1	0	1	1	0	1	0	0	0	0	0	0	0	0	0	0	0	0	0
13	1	0	1	1	0	0	0	0	1	0	0	0	0	0	0	0	0	0	0
14	1	0	1	1	0	1	0	0	1	0	0	0	0	0	0	0	0	0	0
15	1	0	1	1	1	0	0	1	0	0	0	0	1	0	0	0	0	0	0
16	1	0	1	1	0	0	0	1	0	0	0	0	1	0	0	0	0	0	0
17	1	0	1	1	0	0	0	1	0	0	0	0	1	0	0	0	0	0	0
18	1	0	1	1	0	0	0	0	0	1	0	0	0	0	0	0	0	1	0
19	1	0	1	1	0	1	0	0	1	0	0	0	0	0	0	0	0	0	0
20	1	0	1	1	0	1	0	0	1	0	0	0	0	0	0	0	0	0	0
21	1	0	1	1	0	0	0	0	1	0	0	0	0	0	0	0	0	0	0
22	1	0	1	1	0	0	0	1	1	0	0	0	1	0	0	0	0	0	0
23	1	0	1	1	1	0	0	0	1	0	0	0	1	0	0	0	0	0	0
24	1	0	1	1	0	1	0	1	1	0	0	0	1	0	0	0	0	0	0
25	1	0	1	1	0	0	0	1	0	0	0	0	1	0	0	0	0	0	0
26	1	0	1	1	0	0	0	0	1	0	0	0	0	0	0	0	0	0	0
27	1	0	1	1	0	0	0	0	1	0	0	0	0	0	0	0	0	0	0
28	1	0	1	1	1	0	0	0	1	0	0	0	1	0	0	0	0	0	0
29	1	0	1	1	0	0	0	0	1	0	0	0	0	0	0	0	0	0	0
30	1	0	1	1	0	0	0	0	1	0	0	0	0	0	0	0	0	0	0
31	1	0	1	1	0	0	0	0	1	0	0	0	0	0	0	0	0	0	0
32	1	0	1	1	1	0	0	1	0	0	0	0	0	0	0	0	0	0	0
33	1	0	1	1	1	0	0	1	0	0	0	0	0	0	0	0	0	0	0
34	1	0	1	1	0	0	0	0	1	0	0	0	0	0	0	0	0	0	0
35	1	0	1	1	0	0	0	1	0	0	0	0	1	0	0	0	0	0	0
36	1	0	1	1	0	0	0	0	0	0	1	0	0	1	0	0	0	0	0
37	1	0	1	1	0	0	0	0	0	0	0	0	0	0	0	0	1	0	1
38	1	0	1	1	0	1	0	0	0	0	0	0	0	0	0	0	0	0	0
39	1	0	1	1	0	0	0	0	0	1	0	0	0	0	0	1	0	1	0
40	1	0	1	1	1	0	0	0	1	0	0	0	0	0	0	0	0	0	0
41	1	0	1	1	0	0	0	0	1	0	0	0	0	0	0	0	0	0	0
42	1	0	1	1	0	0	0	0	0	1	0	0	0	0	0	1	0	1	0
43	1	0	1	1	0	0	0	0	0	1	0	1	0	1	0	0	0	0	0
44	1	0	1	1	0	0	0	0	1	0	0	0	0	0	0	0	0	0	0
45	1	0	1	1	0	0	0	0	0	1	0	0	0	0	0	1	0	1	0
46	1	0	1	1	0	0	0	0	1	0	0	0	0	0	1	0	0	0	0
47	1	0	1	1	0	0	0	0	1	0	0	0	0	0	0	0	0	0	0

Table S3 Continued.

Code	M41_120	M41_128	M41_130	M12_294	M4_235	M4_243	M4_254	M4_256	M4_258	M4_260	M4_265	M4_266	M4_267	M4_269	M4_271	M4_275	M4_281	M4_285	M4_289
48	1	0	1	1	0	0	0	0	0	0	0	0	0	0	1	0	0	0	0
49	1	0	1	1	0	1	0	0	1	0	0	0	0	0	0	0	0	0	0
50	1	0	1	1	0	0	0	0	1	0	0	0	0	0	0	0	0	0	0
51	1	0	1	1	0	0	0	0	0	0	0	0	0	0	0	0	1	0	1
52	1	0	1	1	0	0	0	0	0	0	1	0	0	0	0	0	0	0	0
53	1	0	1	1	0	0	0	0	0	0	1	0	0	0	0	0	0	0	0
54	1	0	1	1	0	0	0	0	0	0	0	0	0	1	0	0	0	0	0
55	1	0	1	1	0	0	0	0	0	0	0	0	0	1	0	0	0	0	0
56	1	0	1	1	0	0	0	0	0	0	0	0	0	0	1	0	0	0	0
57	1	0	1	1	0	0	0	0	1	0	0	0	0	0	0	0	0	0	0
58	1	0	1	1	0	0	0	0	0	0	0	0	0	0	0	0	1	0	1
59	1	0	1	1	0	0	0	0	0	1	0	0	0	0	0	1	0	1	0
60	1	0	1	1	0	0	0	0	1	0	0	0	1	0	0	0	0	0	0
61	1	0	1	1	0	0	1	0	0	0	0	0	0	0	0	0	1	0	1
62	1	0	1	1	0	0	0	0	1	0	0	0	0	0	0	0	0	0	0
63	1	0	1	1	0	0	0	0	1	0	0	0	0	0	0	0	0	0	0
64	1	0	1	1	0	1	0	0	1	0	0	0	0	0	0	0	0	0	0
65	1	0	1	1	1	0	0	0	1	0	0	0	1	0	0	0	0	0	0
66	1	0	1	1	0	0	1	0	0	1	0	1	0	1	0	0	0	0	0
67	1	0	1	1	0	0	0	0	0	1	0	0	0	0	0	1	0	1	0
68	1	0	1	1	1	0	0	1	0	0	0	0	1	0	0	0	0	0	0
69	1	0	1	1	0	0	0	1	0	0	0	0	1	0	0	0	0	0	0
70	1	0	1	1	0	0	0	0	1	0	0	0	0	0	0	0	0	0	0
71	1	0	1	1	1	0	0	0	1	0	0	0	1	0	0	0	0	0	0
72	1	1	1	1	0	0	0	0	1	0	0	0	0	0	0	0	0	0	0
73	1	0	1	1	1	0	0	0	1	0	0	0	1	0	0	0	0	0	0
74	1	0	1	1	1	0	0	0	1	0	0	0	1	0	0	0	0	0	0
75	1	0	1	1	0	0	0	0	1	0	0	0	0	0	0	0	0	0	0
76	1	0	1	1	0	0	0	0	1	0	0	0	0	0	0	0	0	0	0
77	1	0	1	1	1	0	0	1	0	0	0	0	1	0	0	0	0	0	0
78	1	0	1	1	0	0	0	1	0	0	0	0	0	1	0	0	0	0	0
79	1	0	1	1	0	0	0	0	1	0	0	0	0	0	0	0	0	0	0
80	1	0	1	1	0	0	0	0	1	0	0	0	0	0	0	0	0	0	0
81	1	0	1	1	0	0	0	0	1	0	0	0	0	0	0	0	0	0	0
82	1	0	1	1	0	0	0	0	1	0	0	0	0	0	0	0	0	0	0
83	1	0	1	1	1	0	0	0	1	0	0	0	1	0	0	0	0	0	0
84	1	0	1	1	0	0	0	1	0	0	0	0	0	0	0	0	0	0	0
85	1	0	1	1	0	0	0	1	0	0	0	0	0	0	0	0	0	0	0
86	1	0	1	1	0	0	0	0	0	1	0	0	0	0	0	0	0	0	0
87	1	0	1	1	1	0	0	0	1	0	0	0	1	0	0	0	0	0	0
88	1	0	1	1	1	0	0	0	1	0	0	0	1	0	0	0	0	0	0
89	1	0	1	1	1	0	0	0	1	0	0	0	1	0	0	0	0	0	0
90	1	0	1	1	0	0	0	0	1	0	0	0	0	0	0	0	0	0	0
91	1	0	1	1	0	0	0	1	0	0	0	0	1	0	0	0	0	0	0
92	1	0	1	1	0	0	0	0	0	0	0	0	0	0	1	0	0	0	0

Table S3 Continued.

Code	M24_182	M24_184	M24_187	M24_189	M24_191	M24_193	M24_195	M17_171	M17_173	M8_292	M8_294	M39_152	M39_154	M39_156	M39_158	M39_183	M39_185	M39_187	M39_189
1	0	0	0	0	0	0	1	1	0	0	1	0	0	0	1	0	0	0	0
3	0	0	0	0	0	0	1	1	0	0	1	0	0	0	1	0	0	0	0
4	0	0	0	0	0	0	1	1	0	0	1	0	0	0	1	NA	NA	NA	NA
5	0	0	0	0	1	0	1	1	0	0	1	0	0	1	1	NA	NA	NA	NA
6	0	0	0	0	0	0	1	1	0	0	1	0	0	0	1	0	1	0	0
7	0	1	0	0	0	1	1	1	0	0	1	0	0	0	1	0	0	0	0
9	0	0	0	0	0	0	1	1	0	0	1	0	0	0	1	NA	NA	NA	NA
10	0	0	0	0	0	0	1	1	0	0	1	0	1	0	1	0	0	0	0
11	0	0	0	0	0	0	1	1	0	0	1	0	0	0	1	0	0	0	1
12	0	0	0	0	0	1	1	1	0	0	1	0	0	0	1	0	0	0	0
13	0	0	1	1	0	0	1	1	0	0	1	0	1	0	1	NA	NA	NA	NA
14	0	0	0	0	0	0	1	1	0	0	1	0	0	0	1	0	0	0	0
15	0	0	0	0	0	0	1	1	0	0	1	0	0	0	1	0	0	0	1
16	0	0	0	0	1	1	1	1	0	0	1	0	0	0	1	0	0	1	0
17	0	0	0	0	0	1	1	1	0	0	1	0	0	0	1	0	1	1	0
18	0	0	0	0	0	0	1	1	0	0	1	0	0	0	1	0	0	0	0
19	0	0	0	0	0	1	1	1	0	0	1	0	0	0	1	0	0	0	1
20	0	0	0	0	0	0	1	1	0	0	1	0	0	0	1	0	0	0	0
21	0	0	1	0	0	0	1	1	0	0	1	0	0	0	1	0	1	0	0
22	0	0	0	0	0	1	1	1	0	0	1	0	0	0	1	0	0	1	0
23	0	0	0	0	0	1	1	1	0	0	1	0	0	0	1	0	1	0	0
24	0	0	0	0	1	1	1	1	0	0	1	0	0	0	1	0	1	1	0
25	0	0	0	0	0	1	1	1	0	0	1	0	0	0	1	0	0	1	0
26	NA	NA	NA	NA	NA	NA	NA	1	0	0	1	0	0	0	1	0	0	0	0
27	0	0	0	0	0	0	1	1	0	0	1	0	0	0	1	0	0	0	1
28	0	0	0	0	0	1	1	1	0	0	1	NA	NA	NA	NA	0	0	0	1
29	0	0	0	0	0	1	1	1	0	0	1	0	0	0	1	0	0	0	1
30	0	0	0	0	0	1	1	1	0	0	1	0	0	0	1	0	0	0	0
31	0	0	0	0	0	0	1	1	0	0	1	0	0	0	1	0	0	0	0
32	0	1	0	0	0	1	0	1	0	0	1	0	0	0	1	0	0	0	1
33	0	0	1	0	0	1	0	1	0	0	1	1	0	0	1	0	1	1	0
34	0	0	0	0	0	0	1	1	0	0	1	0	0	0	1	0	0	0	0
35	0	0	0	0	1	1	1	1	0	0	1	0	0	0	1	0	0	1	0
36	0	0	0	0	0	0	1	1	0	0	1	0	0	0	1	0	0	0	0
37	0	0	0	0	0	0	1	1	0	0	1	0	0	0	1	0	0	0	0
38	0	0	0	0	0	0	1	1	0	0	1	0	0	0	1	0	0	0	0
39	0	0	0	0	0	1	1	1	0	0	1	0	0	0	1	0	0	0	0
40	0	0	0	0	1	0	1	1	0	0	1	0	0	0	1	0	0	0	0
41	0	0	0	0	0	0	1	1	0	0	1	0	0	0	1	0	0	1	0
42	0	0	0	0	0	0	1	1	0	0	1	0	0	0	1	0	0	0	0
43	0	0	0	0	0	0	1	1	0	0	1	0	0	0	1	NA	NA	NA	NA
44	0	0	0	0	0	1	1	1	0	0	1	0	0	0	1	0	0	1	0
45	0	0	1	0	1	0	1	1	0	0	1	0	0	0	1	0	0	0	0
46	0	0	0	0	0	0	1	1	1	0	1	0	0	0	1	0	0	0	1
47	0	0	0	0	0	0	1	1	0	0	1	0	0	0	1	0	0	0	1

Table S3 Continued.

Code	M24_182	M24_184	M24_187	M24_189	M24_191	M24_193	M24_195	M17_171	M17_173	M8_292	M8_294	M39_152	M39_154	M39_156	M39_158	M39_183	M39_185	M39_187	M39_189
48	0	0	0	0	0	0	1	1	0	0	1	0	1	0	1	0	0	0	0
49	0	0	0	0	0	0	1	1	0	0	1	0	0	0	1	0	0	0	0
50	0	0	0	0	0	0	1	1	0	0	1	0	0	0	1	0	0	0	0
51	0	0	0	1	0	0	1	1	0	0	1	0	0	0	1	0	0	0	0
52	0	0	0	0	0	0	1	1	0	0	1	0	0	0	1	0	0	0	0
53	0	0	0	0	0	1	1	1	0	0	1	0	0	0	1	0	0	0	0
54	0	0	0	0	0	0	1	1	0	0	1	1	0	1	1	0	0	0	0
55	0	0	0	0	0	0	1	1	0	0	1	0	0	0	1	0	0	0	0
56	0	0	0	0	0	1	1	1	0	0	1	0	0	0	1	NA	NA	NA	NA
57	0	0	0	0	0	0	1	1	0	0	1	0	0	0	1	NA	NA	NA	NA
58	0	0	0	0	0	1	1	1	0	1	1	0	0	0	1	0	0	0	0
59	0	0	0	0	0	0	1	1	0	0	1	0	0	0	1	0	0	0	0
60	0	0	0	0	0	1	1	1	0	0	1	0	0	0	1	0	0	0	1
61	0	0	0	0	0	0	1	1	0	0	1	0	0	0	1	0	0	0	0
62	0	0	0	0	0	1	1	1	0	0	1	0	0	0	1	0	0	0	1
63	0	0	0	0	0	0	1	1	0	0	1	0	0	0	1	0	0	0	0
64	0	0	0	0	0	0	1	1	0	0	1	0	0	0	1	0	0	0	0
65	0	0	0	0	0	1	1	1	0	0	1	0	0	0	1	0	0	0	1
66	0	0	0	0	0	0	1	1	0	0	1	0	0	0	1	0	0	0	0
67	0	0	0	0	0	1	1	1	0	0	1	0	0	0	1	0	0	0	0
68	0	0	0	0	0	1	1	1	0	0	1	0	0	0	1	0	0	0	0
69	0	0	0	0	1	0	1	1	0	0	1	0	1	0	1	1	0	1	0
70	0	0	0	0	0	0	1	1	0	0	1	0	0	0	1	0	0	0	0
71	0	0	0	0	0	0	1	1	0	0	1	0	0	0	1	0	0	0	0
72	0	0	0	0	1	0	1	1	0	0	1	0	0	0	1	0	0	1	0
73	0	0	0	0	0	0	1	1	0	0	1	0	0	0	1	0	0	0	0
74	0	0	0	0	0	1	1	1	0	0	1	0	0	0	1	0	0	1	0
75	0	0	0	0	0	0	1	1	0	0	1	0	0	0	1	0	0	0	0
76	0	0	0	0	0	0	1	1	0	0	1	0	0	0	1	0	0	0	0
77	0	0	0	0	0	0	1	1	0	0	1	0	0	0	1	0	0	1	0
78	0	0	0	0	0	1	1	1	0	0	1	0	1	0	1	0	0	0	0
79	0	0	0	0	0	0	1	1	0	0	1	0	0	0	1	NA	NA	NA	NA
80	0	0	0	0	0	0	1	1	0	0	1	0	0	0	1	0	0	0	0
81	0	0	0	0	0	1	1	1	0	0	1	0	0	0	1	0	0	0	0
82	0	0	0	0	0	1	1	1	0	0	1	0	0	0	1	0	0	0	0
83	0	0	0	0	0	1	1	1	0	0	1	0	0	0	1	1	0	0	0
84	0	0	0	0	0	1	1	1	0	0	1	0	0	0	1	0	0	1	0
85	0	0	0	0	0	1	1	1	0	0	1	0	0	0	1	0	1	0	0
86	1	0	0	0	0	0	1	1	0	0	1	0	0	0	1	0	1	0	0
87	0	0	0	0	0	1	1	1	0	0	1	NA	NA	NA	NA	0	0	0	0
88	0	0	0	0	0	0	1	1	0	0	1	0	0	0	1	0	0	0	0
89	0	0	0	0	0	1	1	1	0	0	1	0	0	0	1	0	0	0	0
90	NA	NA	NA	NA	NA	NA	NA	1	0	0	1	0	0	0	1	NA	NA	NA	NA
91	0	0	0	0	0	1	1	1	0	0	1	0	0	0	1	0	0	1	0
92	0	0	0	0	0	0	1	1	0	0	1	NA	NA	NA	NA	0	0	0	0

Table S3 Continued.

Code	M39_191	M39_193	M39_197	M39_199	M39_201	M39_203	M39_205	M39_207	M39_209	M48_87	M48_89	M48_93	M45_160	M45_161	M45_163	M6_161	M6_163	M6_165	M42_231
1	1	0	0	0	0	0	0	0	0	0	1	1	NA	NA	NA	0	0	1	1
3	0	1	0	0	0	0	0	0	0	0	1	1	1	0	0	0	0	1	1
4	NA	NA	NA	NA	NA	NA	NA	NA	NA	0	1	1	1	1	0	0	0	1	1
5	NA	NA	NA	NA	NA	NA	NA	NA	NA	0	1	1	1	0	0	0	0	1	1
6	1	0	0	0	0	0	0	0	0	0	1	1	1	1	0	0	0	1	1
7	0	0	0	1	0	0	0	0	0	1	1	1	NA	NA	NA	0	1	1	1
9	NA	NA	NA	NA	NA	NA	NA	NA	NA	0	1	1	NA	NA	NA	0	0	1	1
10	0	0	0	0	0	0	1	0	0	0	1	1	1	0	0	0	0	1	1
11	1	0	0	0	0	0	0	0	0	0	1	1	1	0	0	0	0	1	1
12	0	0	1	0	0	0	0	1	0	0	1	1	1	1	0	0	0	1	1
13	NA	NA	NA	NA	NA	NA	NA	NA	NA	0	1	1	1	1	0	0	0	1	1
14	0	1	0	0	0	0	0	0	0	0	1	1	1	0	0	0	0	1	1
15	1	0	0	0	0	0	0	0	0	1	1	1	0	1	0	0	0	1	0
16	0	0	0	0	0	0	0	0	0	1	1	1	0	1	0	0	0	1	1
17	0	0	0	0	0	0	0	0	0	1	1	1	0	1	0	0	0	1	NA
18	0	0	0	0	0	0	1	0	0	0	1	1	1	0	0	0	0	1	1
19	1	0	0	0	0	0	0	0	0	0	1	1	1	0	0	0	0	1	1
20	1	0	0	0	0	0	0	0	0	0	1	1	1	1	0	0	0	1	1
21	0	1	0	0	0	0	0	0	0	0	1	1	1	1	0	0	0	1	0
22	0	0	0	0	0	0	0	0	0	0	1	1	0	1	0	0	0	1	1
23	1	0	0	0	0	0	0	0	0	1	0	1	NA	NA	NA	0	0	1	1
24	0	0	0	0	0	0	0	0	0	1	1	1	1	1	0	0	0	1	1
25	0	0	0	1	0	0	0	0	0	1	1	1	NA	NA	NA	0	0	1	1
26	0	1	0	0	0	0	0	0	0	0	1	1	1	1	0	0	0	1	1
27	1	0	0	0	0	0	0	0	0	0	1	1	1	1	0	0	0	1	1
28	1	0	0	0	0	0	0	0	0	1	1	1	NA	NA	NA	0	0	1	1
29	0	1	0	0	0	0	0	0	0	0	1	1	NA	NA	NA	0	0	1	1
30	1	1	0	0	0	0	0	0	0	1	1	1	0	1	0	0	0	1	0
31	1	1	0	0	0	0	0	0	0	1	0	1	NA	NA	NA	0	0	1	0
32	1	0	0	0	0	0	0	0	0	1	1	1	1	1	0	0	0	1	1
33	1	1	0	0	0	0	0	0	0	1	1	1	NA	NA	NA	0	0	1	1
34	1	1	0	0	0	0	0	0	0	1	1	1	0	1	0	0	0	1	0
35	0	0	0	1	0	0	0	0	0	1	1	1	0	1	0	0	0	1	0
36	1	0	0	0	0	0	0	0	0	0	1	1	1	0	0	0	0	1	1
37	1	0	0	0	0	1	0	0	0	0	1	1	NA	NA	NA	0	0	1	1
38	1	0	0	0	0	0	0	0	0	0	1	1	0	1	0	0	0	1	0
39	0	0	0	0	0	1	1	0	0	0	1	1	NA	NA	NA	0	1	1	1
40	0	0	0	0	0	1	0	0	0	0	1	1	1	0	0	0	0	1	1
41	0	1	0	0	0	0	0	0	0	0	1	1	1	1	0	0	0	1	1
42	0	0	0	0	0	1	1	0	0	0	1	1	NA	NA	NA	0	0	1	1
43	NA	NA	NA	NA	NA	NA	NA	NA	NA	0	1	1	1	1	0	0	0	1	0
44	1	0	0	0	0	0	0	0	0	0	1	1	NA	NA	NA	0	0	1	1
45	0	0	0	0	1	0	1	0	0	0	1	1	1	0	0	0	0	1	1
46	1	0	0	0	0	0	0	0	0	0	1	1	0	1	0	0	0	1	0
47	0	1	0	0	0	0	0	0	0	0	1	1	1	1	0	0	0	1	1

Table S3 Continued.

Code	M39_191	M39_193	M39_197	M39_199	M39_201	M39_203	M39_205	M39_207	M39_209	M48_87	M48_89	M48_93	M45_160	M45_161	M45_163	M6_161	M6_163	M6_165	M42_231
48	0	0	0	0	0	0	1	0	0	0	1	1	NA	NA	NA	0	0	1	0
49	1	0	0	0	0	0	0	0	0	0	1	1	0	1	0	0	0	1	1
50	1	1	0	0	0	0	0	0	0	0	1	1	NA	NA	NA	0	0	1	1
51	0	0	0	0	0	1	0	0	0	0	1	1	1	0	0	0	0	1	1
52	0	0	0	0	0	1	1	0	0	0	1	1	1	0	0	0	1	1	1
53	0	0	0	0	0	1	1	0	0	0	1	1	NA	NA	NA	0	1	1	0
54	0	0	0	0	0	0	1	0	0	0	1	1	1	1	0	0	0	1	1
55	0	0	0	0	0	0	1	0	0	0	1	1	1	0	0	0	0	1	1
56	NA	NA	NA	NA	NA	NA	NA	NA	NA	0	1	1	1	0	0	0	0	1	0
57	NA	NA	NA	NA	NA	NA	NA	NA	NA	0	1	1	1	1	0	0	0	1	1
58	0	0	0	0	0	1	0	0	0	0	1	1	NA	NA	NA	0	0	1	1
59	0	0	0	0	0	0	1	0	0	0	1	1	1	0	0	0	0	1	1
60	1	0	0	0	0	0	0	0	0	0	1	1	1	0	0	0	0	1	1
61	0	0	0	0	0	1	0	0	0	0	1	1	1	0	1	0	1	1	1
62	1	0	0	0	0	0	0	0	0	0	1	1	1	1	0	0	0	1	0
63	0	1	0	0	0	0	0	0	0	0	1	1	1	1	0	0	0	1	1
64	1	0	0	0	0	0	0	0	0	0	1	1	1	1	0	0	0	1	1
65	1	0	0	0	0	0	0	0	0	1	1	1	0	1	0	0	0	1	0
66	0	0	0	0	0	0	1	0	0	0	1	1	1	0	0	0	0	1	1
67	0	0	0	0	0	1	1	0	0	0	1	1	1	0	0	0	1	1	1
68	0	0	0	1	0	0	0	0	1	1	1	1	0	1	0	0	0	1	0
69	0	0	0	0	0	0	0	0	0	1	1	1	0	1	0	1	0	1	0
70	0	1	0	0	0	0	0	0	0	0	1	1	1	1	0	0	0	1	0
71	1	0	0	0	0	0	0	0	0	1	1	1	0	1	0	0	0	1	0
72	1	0	0	0	0	0	0	0	0	0	1	1	1	1	0	0	0	1	1
73	0	1	0	0	0	0	0	0	0	1	1	1	1	1	0	0	0	1	0
74	1	0	0	0	0	0	0	0	0	1	0	1	0	1	0	1	0	1	0
75	1	1	0	0	0	0	0	0	0	0	1	1	1	1	0	0	0	1	1
76	0	1	0	0	0	0	0	0	0	1	1	1	1	1	0	0	0	1	1
77	1	0	0	0	0	0	0	0	0	1	1	1	0	1	0	0	0	1	0
78	0	0	0	1	0	1	0	0	0	1	1	1	1	1	0	0	0	1	0
79	NA	NA	NA	NA	NA	NA	NA	NA	NA	0	1	1	1	1	0	0	0	1	0
80	0	1	0	0	0	0	0	0	0	0	1	1	1	1	0	0	0	1	1
81	0	1	0	0	0	0	0	0	0	0	1	1	1	1	0	0	0	1	1
82	1	1	0	0	0	0	0	0	0	0	1	1	1	1	0	0	0	1	1
83	1	0	0	0	0	0	0	0	0	1	0	1	0	1	0	0	0	1	1
84	0	0	0	0	0	0	0	0	0	0	1	1	1	1	0	0	0	1	0
85	0	0	0	0	0	0	0	0	0	0	1	1	1	1	0	0	0	1	0
86	1	0	0	0	0	0	0	0	0	0	1	1	0	1	0	0	0	1	1
87	1	0	0	0	0	0	0	0	0	1	0	1	1	1	0	0	0	1	0
88	0	1	0	0	0	0	0	0	0	1	1	1	1	1	0	0	0	1	0
89	1	0	0	0	0	0	0	0	0	1	0	1	0	1	0	0	0	1	1
90	NA	NA	NA	NA	NA	NA	NA	NA	NA	NA	NA	NA	1	1	0	0	0	1	1
91	0	0	0	0	0	0	0	0	0	1	1	1	0	1	0	0	0	1	0
92	0	0	0	0	0	0	1	0	0	0	1	1	1	0	0	0	0	1	0

Table S3 Continued.

Code	M42_233	M42_237	M42_239	M42_245	M42_251	M42_259	M42_261	M42_263	M44_209	M44_215	M44_221	M44_223	M54_128	M54_130	M28_161	M28_162	M28_169	M28_170	M35_170
1	1	0	0	0	1	0	0	0	NA	NA	NA	NA	NA	NA	NA	NA	NA	NA	0
3	1	0	0	0	0	0	1	0	1	1	0	0	1	1	1	1	0	0	0
4	1	0	0	0	1	0	1	0	1	1	0	0	1	1	1	1	0	0	0
5	1	0	0	0	0	0	0	0	1	1	0	0	1	1	1	1	0	0	0
6	1	1	0	0	0	0	0	0	1	1	0	0	1	1	1	1	0	0	1
7	1	0	0	0	0	0	0	0	NA	NA	NA	NA	NA	NA	0	1	0	0	0
9	1	0	0	0	1	0	0	0	1	1	0	0	NA	NA	1	1	0	0	0
10	1	0	0	0	0	0	1	0	1	1	0	0	1	1	1	1	0	0	0
11	1	0	0	0	0	0	1	0	1	1	0	1	1	1	1	1	0	0	0
12	1	0	0	0	0	0	1	0	1	1	0	0	1	1	1	1	0	0	0
13	1	0	0	0	0	0	0	0	1	1	0	1	1	1	1	1	0	0	1
14	0	0	0	0	1	0	0	0	1	1	0	1	1	1	1	1	0	0	0
15	0	1	0	0	1	0	0	0	NA	NA	NA	NA	NA	NA	NA	NA	NA	NA	0
16	0	0	0	0	1	0	0	0	NA	NA	NA	NA	NA	NA	NA	NA	NA	NA	0
17	NA	NA	NA	NA	NA	NA	NA	NA	NA	NA	NA	NA	NA	NA	NA	NA	NA	NA	0
18	1	0	0	0	1	0	0	0	1	1	0	1	1	1	1	1	0	0	0
19	0	0	0	0	1	0	0	0	NA	NA	NA	NA	NA	NA	NA	NA	NA	NA	0
20	0	0	0	0	1	0	0	0	1	1	0	1	1	1	1	1	0	0	0
21	1	0	0	0	1	0	0	0	1	1	0	0	1	1	NA	NA	NA	NA	0
22	0	0	0	1	0	0	0	0	NA	NA	NA	NA	NA	NA	NA	NA	NA	NA	NA
23	0	0	0	1	0	0	0	0	NA	NA	NA	NA	NA	NA	1	1	0	0	0
24	0	0	0	0	1	0	0	0	NA	NA	NA	NA	1	1	1	1	0	0	0
25	0	0	0	0	1	0	0	0	1	1	1	0	1	1	1	1	0	0	0
26	0	0	0	0	1	0	0	0	1	1	0	0	1	1	1	1	0	0	0
27	0	0	0	0	1	0	0	0	1	1	0	0	1	1	1	1	0	0	0
28	0	0	0	0	0	0	0	0	NA	NA	NA	NA	NA	NA	1	1	0	0	0
29	0	0	0	0	1	0	0	0	NA	NA	NA	NA	NA	NA	NA	NA	NA	NA	0
30	0	0	0	0	1	0	0	0	1	1	0	0	1	1	1	1	0	0	0
31	0	0	0	0	1	0	0	0	NA	NA	NA	NA	1	1	1	1	0	0	0
32	0	0	0	1	0	0	0	0	1	1	0	0	1	1	1	1	0	0	0
33	0	0	0	0	0	0	0	0	NA	NA	NA	NA	NA	NA	NA	NA	NA	NA	NA
34	0	0	0	0	1	0	0	0	1	1	0	0	1	1	1	1	0	0	0
35	0	1	0	0	1	0	0	0	1	1	0	0	1	1	1	1	0	0	0
36	1	0	0	0	0	0	0	0	1	0	0	1	1	1	1	1	0	0	0
37	0	1	0	0	0	0	0	0	NA	NA	NA	NA	1	1	1	1	0	0	0
38	0	0	0	0	1	0	0	0	1	1	0	0	1	1	1	1	0	0	0
39	0	0	0	0	0	0	0	0	NA	NA	NA	NA	NA	NA	0	1	0	0	0
40	0	1	0	0	0	0	0	0	1	0	1	1	1	1	1	1	0	0	0
41	0	0	0	0	1	0	0	0	1	1	0	0	1	1	1	1	0	0	0
42	0	0	0	0	0	0	0	1	NA	NA	NA	NA	1	1	1	1	1	1	0
43	0	0	0	0	1	0	0	0	NA	NA	NA	NA	NA	NA	0	1	0	0	0
44	0	0	0	0	1	0	0	0	NA	NA	NA	NA	1	1	0	1	0	0	0
45	0	0	0	0	0	0	0	1	1	1	0	0	1	1	1	1	1	1	0
46	0	0	1	0	1	0	0	0	1	1	0	0	1	1	1	1	0	0	0
47	0	0	0	0	1	0	0	0	1	1	0	0	1	1	1	1	0	0	0

Table S3 Continued.

Code	M42_233	M42_237	M42_239	M42_245	M42_251	M42_259	M42_261	M42_263	M44_209	M44_215	M44_221	M44_223	M54_128	M54_130	M28_161	M28_162	M28_169	M28_170	M35_170
48	1	0	0	0	0	0	0	0	NA	NA	NA	NA	1	1	1	1	0	0	0
49	0	0	0	0	1	0	0	0	1	1	0	1	1	1	1	1	0	0	0
50	0	1	0	0	1	0	0	0	NA	NA	NA	NA	NA	NA	0	1	0	0	0
51	0	1	0	0	0	0	0	0	1	1	0	1	1	1	1	1	0	0	0
52	1	0	0	0	0	0	0	1	1	1	0	0	1	1	1	1	0	0	0
53	1	0	0	0	0	0	0	1	1	1	1	0	1	1	1	1	0	0	0
54	1	1	0	0	1	0	0	0	1	1	1	0	NA	NA	1	1	0	0	0
55	1	0	0	0	0	0	0	0	1	1	0	0	1	1	1	1	0	0	0
56	1	0	0	0	1	0	0	0	1	1	0	1	1	1	1	1	0	0	0
57	0	0	0	0	1	0	0	0	1	1	0	0	1	1	1	1	0	0	0
58	1	0	0	0	0	0	0	0	NA	NA	NA	NA	NA	NA	NA	NA	NA	NA	0
59	0	0	0	0	0	0	0	1	1	1	0	0	1	1	1	1	1	1	0
60	0	0	0	0	0	0	0	1	1	1	0	0	1	1	1	1	1	1	0
61	0	1	0	0	0	0	0	0	1	1	0	1	1	1	1	1	0	0	0
62	1	0	0	0	1	0	0	0	1	1	0	0	1	1	1	1	0	0	0
63	0	0	0	0	1	0	0	0	1	1	0	0	1	1	1	1	0	0	0
64	0	0	0	0	1	0	0	0	1	1	0	1	1	1	1	1	0	0	0
65	0	0	0	1	1	0	0	0	1	1	0	0	1	1	1	1	0	0	1
66	1	0	0	0	0	0	0	0	1	1	0	0	1	1	1	1	0	0	0
67	0	0	0	0	0	0	0	1	1	1	0	0	1	1	1	1	1	1	0
68	0	0	0	1	1	0	0	0	1	1	0	0	1	1	1	1	0	0	0
69	0	1	0	1	1	0	0	0	1	1	0	0	1	1	1	1	0	0	0
70	1	0	0	0	1	0	0	0	1	1	0	0	1	1	1	1	0	0	0
71	0	0	0	1	1	0	0	0	1	1	0	0	1	1	1	1	0	0	0
72	0	0	0	0	1	0	0	0	1	1	0	0	1	1	1	1	0	0	0
73	0	0	0	0	1	0	0	1	1	1	0	0	1	1	1	1	1	1	0
74	1	0	0	0	1	0	0	0	0	1	0	0	1	1	1	1	0	0	1
75	0	0	0	0	1	0	0	0	1	1	0	0	1	1	1	1	0	0	0
76	0	0	0	0	1	0	0	0	1	1	0	0	1	1	1	1	0	0	0
77	0	0	0	0	1	0	0	0	1	1	0	0	1	1	1	1	0	0	0
78	0	0	0	0	1	0	0	1	1	1	0	0	1	1	1	1	1	1	0
79	1	0	0	0	1	0	0	0	1	1	0	0	1	1	1	1	0	0	0
80	0	0	0	0	1	0	0	0	1	1	0	0	1	1	1	1	0	0	1
81	0	0	0	0	1	0	0	0	1	1	0	0	1	1	1	1	0	0	0
82	0	0	0	0	1	0	0	0	1	1	0	0	1	1	1	1	0	0	0
83	0	0	0	1	0	0	0	0	1	1	0	0	1	1	1	1	0	0	0
84	1	0	0	0	0	0	0	0	1	1	0	0	1	1	1	1	0	0	0
85	1	0	0	1	0	0	0	0	1	1	0	0	1	1	1	1	0	0	0
86	0	0	0	0	1	0	0	0	1	1	0	0	1	1	1	1	0	0	0
87	0	0	0	1	1	0	0	1	1	1	0	0	1	1	1	1	0	0	0
88	0	0	0	0	1	1	0	1	1	1	0	0	1	1	1	1	0	0	0
89	0	0	0	1	0	0	0	0	1	1	0	0	1	1	1	1	0	0	0
90	0	0	0	0	1	0	0	0	1	1	0	0	1	1	1	1	0	0	0
91	0	1	0	0	1	0	0	0	1	1	0	0	1	1	1	1	0	0	0
92	1	0	0	0	1	0	0	0	1	1	0	1	1	1	1	1	0	0	0

Table S3 Continued.

Code	M35_172	M35_185	M35_187	M35_192	M35_198	M35_202	M35_204	M35_207	M35_210	M35_211	M35_213	M35_215	M35_220	M35_222	M35_223	M35_226	M53_141	M53_143	M53_145
1	1	0	0	1	0	0	0	1	0	0	0	0	0	0	1	0	0	1	1
3	0	0	0	1	0	0	0	1	0	0	0	1	0	0	0	0	1	1	0
4	0	0	0	1	0	0	0	1	0	0	0	0	0	0	0	0	1	1	0
5	1	0	0	1	0	0	0	1	0	0	0	0	0	0	0	0	1	1	0
6	0	0	0	1	0	0	0	1	0	0	0	0	0	1	0	0	1	1	1
7	1	0	0	0	0	1	0	1	0	0	0	0	0	0	0	0	0	1	1
9	1	0	0	1	0	0	0	1	0	0	0	0	0	0	0	0	1	1	1
10	0	0	0	1	0	0	0	0	0	0	1	1	0	0	0	0	0	1	1
11	1	1	0	1	0	0	0	1	0	0	0	0	0	0	1	0	0	1	1
12	0	0	0	1	0	0	0	1	0	0	0	0	0	0	1	0	0	1	1
13	0	1	0	1	0	0	0	1	0	0	0	0	0	0	1	0	1	0	1
14	1	1	0	1	0	0	0	1	0	0	0	0	0	0	1	0	1	0	1
15	1	0	0	0	0	0	0	0	0	0	0	0	0	0	0	0	0	1	1
16	1	0	0	0	0	0	0	1	0	0	0	0	0	0	0	0	0	1	1
17	1	0	0	0	0	0	0	0	0	0	0	0	0	0	0	0	0	0	1
18	1	0	0	1	0	0	0	0	0	0	0	1	0	0	1	0	0	1	1
19	0	1	0	1	0	0	0	0	0	0	0	1	0	0	1	0	0	1	1
20	1	1	0	1	0	0	0	0	0	0	0	1	0	0	1	0	0	1	1
21	0	0	0	0	0	0	0	1	0	0	0	0	0	1	0	0	1	0	1
22	NA	NA	NA	NA	NA	NA	NA	NA	NA	NA	NA	NA	NA	NA	NA	NA	0	1	1
23	1	0	0	1	0	0	0	0	0	0	0	1	0	1	0	0	0	0	1
24	1	0	0	1	0	0	0	0	0	0	0	0	0	1	1	0	0	0	1
25	1	0	0	1	0	0	0	0	0	0	0	0	0	1	0	0	0	1	1
26	1	0	0	1	0	0	0	1	0	0	0	0	0	1	0	0	1	0	1
27	1	0	0	1	0	0	0	1	0	0	0	0	0	1	0	0	0	1	1
28	0	0	0	0	0	0	0	0	0	0	0	1	0	0	1	0	0	1	1
29	1	0	0	1	0	0	0	0	0	0	0	1	0	1	1	0	0	1	1
30	1	0	0	1	0	0	0	0	0	0	0	0	0	1	0	0	0	1	1
31	1	1	0	1	0	0	0	0	0	0	0	0	0	1	0	0	0	0	1
32	1	0	0	1	0	0	0	1	0	0	0	1	0	0	0	0	0	0	1
33	NA	NA	NA	NA	NA	NA	NA	NA	NA	NA	NA	NA	NA	NA	NA	NA	0	1	1
34	1	0	0	1	0	0	0	0	0	0	0	0	0	1	0	0	0	1	1
35	1	0	0	1	0	0	0	0	0	0	0	0	0	1	0	0	0	1	1
36	0	0	0	1	0	0	0	0	0	0	1	1	0	0	0	0	0	1	1
37	0	0	1	1	0	0	1	0	0	0	0	0	0	0	0	0	0	1	0
38	1	0	0	1	0	0	0	1	0	0	0	0	0	1	0	0	1	0	1
39	1	0	0	0	1	0	0	0	0	0	0	0	0	0	0	1	0	1	1
40	0	0	1	1	0	0	1	0	0	0	0	0	0	0	0	0	0	1	0
41	1	0	0	1	0	0	0	1	0	0	0	0	0	1	0	0	1	0	1
42	0	0	0	0	1	0	0	0	0	0	0	0	0	0	0	1	0	1	1
43	1	0	0	1	0	0	0	1	0	0	0	0	0	1	0	0	0	0	1
44	1	0	0	1	0	0	0	1	0	0	0	0	0	1	0	0	1	0	1
45	0	0	1	1	1	0	0	0	0	0	0	0	0	0	0	1	0	1	0
46	1	0	0	0	1	0	0	0	1	0	0	1	0	0	0	0	0	1	1
47	1	0	0	1	0	0	0	1	0	0	0	0	0	1	0	0	1	0	1

Table S3 Continued.

Code	M35_172	M35_185	M35_187	M35_192	M35_198	M35_202	M35_204	M35_207	M35_210	M35_211	M35_213	M35_215	M35_220	M35_222	M35_223	M35_226	M53_141	M53_143	M53_145
48	0	0	0	1	0	0	0	1	0	0	0	0	0	0	0	0	0	1	0
49	1	1	0	0	0	0	0	0	0	0	0	1	0	0	1	0	0	1	1
50	1	1	0	1	0	0	0	1	0	0	0	1	0	0	0	0	1	0	1
51	0	0	1	1	0	0	1	0	0	0	0	0	1	0	0	0	0	1	0
52	0	0	0	0	0	0	0	0	0	0	0	1	0	0	0	1	0	1	1
53	0	0	1	1	0	0	0	0	0	0	1	0	0	0	0	1	0	1	1
54	1	0	0	1	0	0	1	1	0	0	0	0	0	1	0	0	1	1	1
55	0	0	0	0	0	0	0	0	0	0	0	1	0	0	0	1	0	1	1
56	0	0	0	1	0	0	0	1	0	0	0	0	0	0	0	0	0	1	0
57	1	0	0	1	0	0	0	1	0	0	0	0	0	1	0	0	1	0	1
58	1	0	0	1	0	0	0	1	0	0	0	0	0	1	0	1	1	1	1
59	0	0	1	1	1	0	0	0	0	0	0	0	0	0	0	1	0	1	0
60	0	0	1	1	1	0	0	0	0	0	0	0	0	0	0	1	0	1	0
61	0	0	1	1	0	0	1	0	0	0	0	0	1	0	0	0	0	1	0
62	1	0	0	1	0	0	0	1	0	0	0	0	0	1	0	0	1	0	1
63	1	0	0	1	0	0	0	1	0	0	0	0	0	1	0	0	1	0	1
64	1	1	0	1	0	0	0	0	0	0	0	1	0	0	1	0	0	1	1
65	0	0	0	1	0	0	0	0	0	0	0	0	0	1	0	0	0	0	1
66	1	0	0	1	0	0	0	1	0	0	0	0	0	0	0	0	1	1	0
67	0	0	1	1	1	0	0	0	0	0	0	0	0	0	0	1	0	1	0
68	1	0	0	1	0	0	0	0	0	0	0	0	0	1	0	0	0	0	1
69	1	0	0	1	0	0	0	0	0	0	0	0	1	0	0	0	0	1	1
70	1	0	0	1	0	0	0	1	0	0	0	0	0	1	0	0	1	0	1
71	1	0	0	1	0	0	0	0	0	0	0	0	0	1	0	0	0	0	1
72	1	0	0	1	0	0	0	1	0	0	0	0	0	1	0	0	0	1	1
73	1	0	1	1	0	0	0	0	0	0	0	1	0	0	0	1	0	1	1
74	0	1	0	1	0	0	0	1	0	0	0	0	0	1	0	0	0	1	1
75	1	0	0	1	0	0	0	1	0	0	0	0	0	1	0	0	1	0	1
76	1	0	0	1	0	0	0	1	0	0	0	0	0	1	0	0	1	0	1
77	1	0	0	1	0	0	0	0	0	1	0	0	0	1	0	0	0	0	1
78	1	0	1	1	0	0	0	0	0	0	0	1	0	0	0	1	0	1	1
79	1	0	0	1	0	0	0	1	0	0	0	0	0	1	0	0	0	1	1
80	0	0	0	1	0	0	0	1	0	0	0	0	0	1	0	0	1	0	1
81	1	0	0	1	0	0	0	1	0	0	0	0	0	1	0	0	1	0	1
82	1	0	0	1	0	0	0	1	0	0	0	0	0	1	0	0	1	0	1
83	1	0	0	1	0	0	0	1	0	0	0	1	0	1	0	0	1	0	1
84	1	0	0	1	0	0	0	1	0	0	0	0	0	1	0	0	1	1	0
85	1	0	0	1	0	0	0	1	0	0	0	0	0	1	0	0	1	1	1
86	1	0	0	1	0	0	0	1	0	0	0	1	0	0	1	0	0	1	1
87	1	0	0	1	0	0	0	0	0	0	0	1	0	0	0	1	0	1	1
88	1	0	0	1	0	0	0	0	0	0	0	1	0	0	0	1	0	1	1
89	1	0	0	1	0	0	0	1	0	0	0	1	0	1	0	0	0	1	1
90	1	0	0	1	0	0	0	1	0	0	0	0	0	1	0	0	0	1	1
91	1	0	0	1	0	0	0	0	0	0	0	0	0	1	0	0	0	1	1
92	0	0	0	1	0	0	0	1	0	0	0	0	0	0	0	0	0	1	0

Table S3 Continued.

Code	M31_212	M31_220	M31_223	M31_224	M31_225	M31_236	M34_157	M34_159	M34_163	M34_165	M34_167	M34_168	M34_169	M34_171	M34_175	M34_177
1	0	0	1	0	0	0	0	0	0	1	0	0	1	0	0	0
3	0	1	0	1	0	0	0	0	0	1	0	0	1	0	0	0
4	0	1	0	1	0	0	0	0	0	1	0	0	0	0	0	0
5	0	1	0	1	0	0	0	0	0	1	0	0	0	0	0	0
6	0	1	1	1	0	0	0	0	0	1	0	0	1	0	0	0
7	NA	NA	NA	NA	NA	NA	NA	NA	NA	NA	NA	NA	NA	NA	NA	NA
9	0	1	1	1	0	0	0	0	0	1	0	0	0	0	0	0
10	0	1	0	0	0	0	0	0	0	1	0	0	1	0	0	0
11	0	0	1	1	0	0	0	0	0	1	0	0	1	0	0	0
12	0	1	0	1	0	0	0	0	0	1	0	0	1	0	0	0
13	0	1	1	0	0	0	0	0	0	1	0	0	1	0	0	0
14	0	1	0	0	0	0	0	0	0	0	0	0	1	0	0	0
15	0	0	1	0	0	0	NA	NA	NA	NA	NA	NA	NA	NA	NA	NA
16	NA	NA	NA	NA	NA	NA	NA	NA	NA	NA	NA	NA	NA	NA	NA	NA
17	NA	NA	NA	NA	NA	NA	NA	NA	NA	NA	NA	NA	NA	NA	NA	NA
18	0	1	1	0	0	0	0	0	0	1	0	0	1	0	0	0
19	NA	NA	NA	NA	NA	NA	0	0	0	0	0	0	1	0	0	0
20	0	1	1	0	0	0	0	0	0	0	0	0	1	0	0	0
21	0	1	1	0	0	0	0	0	0	1	0	0	1	0	0	0
22	NA	NA	NA	NA	NA	NA	NA	NA	NA	NA	NA	NA	NA	NA	NA	NA
23	NA	NA	NA	NA	NA	NA	0	0	0	0	0	1	1	0	0	0
24	NA	NA	NA	NA	NA	NA	0	0	0	0	0	0	1	1	0	0
25	0	0	1	0	0	0	0	0	0	0	0	0	0	1	1	0
26	0	0	1	1	0	0	0	0	0	1	0	0	1	0	0	0
27	0	0	1	1	0	0	0	0	0	1	0	0	1	0	0	0
28	NA	NA	NA	NA	NA	NA	NA	NA	NA	NA	NA	NA	NA	NA	NA	NA
29	NA	NA	NA	NA	NA	NA	0	0	0	1	0	0	1	0	0	0
30	0	0	1	0	0	0	0	0	0	0	0	0	1	0	0	0
31	NA	NA	NA	NA	NA	NA	0	0	0	0	0	1	1	0	0	0
32	0	0	1	0	0	0	0	0	0	0	0	0	1	0	0	0
33	NA	NA	NA	NA	NA	NA	NA	NA	NA	NA	NA	NA	NA	NA	NA	NA
34	0	0	1	0	0	0	0	0	0	0	0	0	1	0	0	0
35	0	0	1	0	0	0	0	0	0	0	0	0	1	1	0	0
36	0	1	0	0	0	0	0	0	0	1	1	0	0	0	0	0
37	NA	NA	NA	NA	NA	NA	0	0	0	1	0	0	1	0	0	0
38	0	0	1	0	0	0	0	0	0	1	0	0	0	1	0	0
39	NA	NA	NA	NA	NA	NA	NA	NA	NA	NA	NA	NA	NA	NA	NA	NA
40	0	1	1	0	0	0	0	0	0	1	0	0	1	0	0	0
41	0	0	1	1	0	0	0	0	0	1	0	0	1	0	0	0
42	NA	NA	NA	NA	NA	NA	0	0	1	0	0	0	1	0	0	0
43	NA	NA	NA	NA	NA	NA	0	0	0	0	0	0	1	0	0	0
44	NA	NA	NA	NA	NA	NA	0	0	0	1	0	0	1	0	0	0
45	0	1	0	0	0	0	0	0	1	0	0	0	1	0	0	0
46	0	0	1	0	1	0	0	0	1	1	0	0	0	1	0	1
47	0	0	1	1	0	0	0	0	0	1	0	0	1	0	0	0

Table S3 Continued.

Code	M31_212	M31_220	M31_223	M31_224	M31_225	M31_236	M34_157	M34_159	M34_163	M34_165	M34_167	M34_168	M34_169	M34_171	M34_175	M34_177
48	NA	NA	NA	NA	NA	NA	1	0	1	0	0	0	0	0	0	0
49	0	1	1	0	0	0	0	0	0	0	0	0	1	0	0	0
50	NA	NA	NA	NA	NA	NA	0	0	0	0	0	0	1	0	0	0
51	0	1	0	0	0	0	0	0	0	1	0	0	1	0	0	0
52	0	1	0	0	0	0	0	0	0	1	0	0	1	0	0	0
53	0	1	0	1	0	0	0	0	1	1	0	0	0	0	0	0
54	NA	NA	NA	NA	NA	NA	0	0	0	1	0	0	1	1	0	0
55	0	1	0	0	0	0	0	0	0	1	0	0	1	0	0	0
56	1	0	0	1	1	1	1	0	1	0	0	0	0	0	0	0
57	0	0	1	1	0	0	0	0	0	1	0	0	1	0	0	0
58	NA	NA	NA	NA	NA	NA	NA	NA	NA	NA	NA	NA	NA	NA	NA	NA
59	0	0	0	1	0	0	0	0	1	0	0	0	1	0	0	0
60	0	1	0	1	0	0	0	0	1	0	0	0	1	0	0	0
61	0	1	0	0	0	0	0	0	0	1	0	0	1	0	0	0
62	0	1	1	0	0	0	0	0	0	1	0	0	1	0	0	0
63	0	0	1	1	0	0	0	0	0	1	0	0	1	0	0	0
64	0	1	1	0	0	0	0	0	0	0	0	0	1	0	0	0
65	0	0	1	0	0	0	0	0	0	0	0	0	1	0	0	0
66	0	1	0	1	0	0	0	0	0	1	0	0	0	0	0	0
67	0	0	0	1	0	0	0	0	1	0	0	0	1	0	0	0
68	0	0	1	0	0	0	0	0	0	0	0	0	1	0	1	0
69	0	0	1	0	0	0	0	0	0	0	0	0	1	1	1	0
70	0	1	1	0	0	0	0	0	0	1	0	0	1	0	0	0
71	0	0	1	0	0	0	0	0	0	0	0	0	1	0	0	0
72	0	0	1	1	0	0	0	0	0	1	0	0	1	0	0	0
73	0	0	1	1	0	0	0	0	1	0	0	0	1	0	0	0
74	0	0	1	0	0	0	0	0	0	0	0	0	1	0	0	0
75	0	0	1	1	0	0	0	0	0	1	0	0	1	0	0	0
76	0	0	1	1	0	0	0	0	0	1	0	0	1	0	0	0
77	0	0	1	0	0	0	0	0	0	0	0	0	1	0	0	0
78	NA	NA	NA	NA	NA	NA	0	1	1	1	0	0	1	0	0	0
79	0	1	1	0	0	0	0	0	0	1	0	0	1	0	0	0
80	0	0	1	1	0	0	0	0	0	1	0	0	1	0	0	0
81	0	0	1	1	0	0	0	0	0	1	0	0	1	0	0	0
82	0	0	1	1	0	0	0	0	0	1	0	0	1	0	0	0
83	0	0	1	1	0	0	0	0	0	1	1	0	1	0	0	0
84	0	1	1	0	0	0	0	0	0	1	0	0	0	1	0	0
85	0	1	1	0	0	0	0	0	0	1	0	0	1	1	0	0
86	0	1	1	1	0	0	0	0	0	1	0	0	1	0	0	0
87	0	0	1	1	0	0	0	0	1	0	0	0	1	0	0	0
88	0	0	1	1	0	0	0	0	1	0	0	0	1	0	0	0
89	0	0	1	0	0	0	0	0	1	0	0	0	1	0	0	0
90	0	1	1	0	0	0	0	0	0	1	0	0	1	0	0	0
91	0	0	1	0	0	0	0	0	0	0	0	0	1	1	0	0
92	1	0	0	1	1	1	1	0	1	0	0	0	0	0	0	0
